# KREAP: an automated Galaxy platform to quantify *in vitro* re-epithelialization kinetics

**DOI:** 10.1093/gigascience/giy078

**Published:** 2018-06-28

**Authors:** Marcela M Fernandez-Gutierrez, David B H van Zessen, Peter van Baarlen, Michiel Kleerebezem, Andrew P Stubbs

**Affiliations:** 1 TI Food and Nutrition, Nieuwe Kanaal 9-A, 6709 PA, Wageningen, The Netherlands; 2 Host-Microbe Interactomics, Animal Sciences Group, Wageningen University & Research, De Elst 1, 6708 WD, Wageningen, The Netherlands; 3 Department of Bioinformatics, Erasmus University Medical Centre, Wytemaweg 80, 3015 CN, Rotterdam, The Netherlands

**Keywords:** Galaxy, scratch assay, high-throughput, cell migration, re-epithelialization, image analysis, workflow, modeling, wound healing

## Abstract

**Background:**

*In vitro* scratch assays have been widely used to study the influence of bioactive substances on the processes of cell migration and proliferation that are involved in re-epithelialization. The development of high-throughput microscopy and image analysis has enabled scratch assays to become compatible with high-throughput research. However, effective processing and in-depth analysis of such high-throughput image datasets are far from trivial and require integration of multiple image processing and data extraction software tools.

**Findings:**

We developed and implemented a kinetic re-epithelialization analysis pipeline (KREAP) in Galaxy. The KREAP toolbox incorporates freely available image analysis tools and automatically performs image segmentation and feature extraction of each image series, followed by automatic quantification of cells inside and outside the scratched area over time. The enumeration of infiltrating cells over time is modeled to extract three biologically relevant parameters that describe re-epithelialization kinetics. The output of the tools is organized, displayed, and saved in the Galaxy environment for future reference.

**Conclusions:**

The KREAP toolbox in Galaxy provides an open-source, easy-to-use, web-based platform for reproducible image processing and data analysis of high-throughput scratch assays. The KREAP toolbox could assist a broad scientific community in the discovery of compounds that are able to modulate re-epithelialization kinetics.

## Findings

### Background

Cell migration and proliferation play an essential role in a variety of physiological processes, including embryogenesis, angiogenesis, skin and intestinal renewal, and wound repair [1, 2]. Deregulation of these processes can contribute to the development and progression of multiple diseases such as osteoporosis, rheumatoid arthritis, vascular disease, and cancer [1]. Therefore, the study of the molecular mechanisms that underlie the processes of cell migration and proliferation is not only important for obtaining fundamental scientific insight but it is also essential for the development of effective therapeutic strategies that could modulate these processes when they have become dysregulated.

The *in vitro* scratch assay is a well-established and widely used method to study cell migration and proliferation [3-5]. The assay is based on the introduction of a scratch into a confluent epithelial cell monolayer to create a cell-free area. Cells migrate and proliferate into the site of injury in a process known as re-epithelialization [6]. This process is typically monitored by acquisition of images at the beginning and at one or more fixed time points during re-epithelialization. The image series obtained for a particular treatment is then compared to that of the nontreated control to determine the treatment's modulatory capacity in the healing process. The development and constant improvement of image segmentation algorithms over the past decades have enabled the transition from manual quantification of the scratch area to automated analysis that is compatible with high-throughput screenings [7-9].

CellProfiler [10] and ImageJ [11] are freely available image analysis software tools that allow scientists with limited programming skills to conduct efficient image segmentation and feature extraction of high-throughput image datasets. However, scripting and parsing of data are often necessary to optimally use the capacities of these tools, requiring programming skills that many biologists do not have. Commercial software such as FCS Express Image Cytometry (De Novo Software, CA, USA) [12] and Image-Pro Premier (Media Cybernetics, WA, USA), among others, provide alternatives to ease data analysis but require the purchase of licenses. TScratch was the first open-source application exclusively designed to perform automated analyses of scratch assays by determining the percentage of open wound area, but lacks the ability to extract real-time kinetic data [13]. More recently, CellMissy was developed as an open-source software to determine the area (µm^2^) of wound closure over time, as well as the mean collective cell migration velocity (µm/hour) for a given condition [14]. Nevertheless, the biological properties of certain cells may render these calculations challenging. For instance, poorly adherent cells (e.g. FHs-74 small intestinal cells) do not migrate collectively but rather detach and migrate individually in uneven patterns, making wound area measurements inaccurate [15]. Thus, there is still a need for an open-source platform based on single-cell recognition that could integrate different validated tools for image segmentation, visualization, and data analysis of the processes of cell migration and proliferation involved in wound repair.

We developed and implemented a kinetic re-epithelialization analysis pipeline (KREAP) in Galaxy [17] (Galaxy, RRID:SCR_006281) [16] to deliver a web browser-based application for quantitative analysis of *in vitro* scratch assays based on single-cell recognition. The user needs to download and install a virtual machine (VM) containing a fully operational KREAP Galaxy installation. Once the VM is installed, the user can upload the images from a multiwell plate experiment, together with its corresponding index file, into the VM and press the *Execute* button to automatically perform single-cell segmentation and feature extraction across all images. Enumeration of cells inside and outside the scratched area is also carried out automatically over the time series. Based on the number of cells infiltrating the scratch over time, KREAP extracts three biologically comprehensive parameters that describe the kinetics of re-epithelialization. In addition, the user's history is saved in the VM for future consultation and the results can be easily shared with other users by downloading the history of multiple experiments. Taken together, we provide a platform that enables reproducible data processing and analysis of high-throughput scratch assays—from raw images to re-epithelialization kinetics—that facilitates screenings of substances that may influence re-epithelialization.

### Implementation

The scratch assay analysis workflow was developed within our own laboratory [18] (also see Methods) and involved a multi-software approach to acquire images, perform image analysis, visualize extracted data, and model re-epithelialization kinetics based on the enumeration of cells migrating into the scratch area over time. CellProfiler [19] was used in the original workflow and implemented in the KREAP toolbox (version 2.2.0) to perform automated segmentation and feature extraction of image series. FCS Express 4 Plus (De Novo Software, CA, USA) was originally used to relate the features extracted by CellProfiler back to the raw images and to enumerate the cells infiltrating the scratched area over time [18]. Since FCS Express 4 Plus requires the purchase of a license, we developed and implemented an R script [20], now part of the KREAP toolbox, that can automatically recognize the scratch boundaries and determine the number of cells inside and outside of the scratch over time. Modeling of re-epithelialization kinetics was programmed in R and also implemented in the KREAP toolbox workflow. The workflow is provided in a fully operational Galaxy installation inside of a VM that can be retrieved from the KREAP home page [21]. The VM can be executed using the freely available Oracle VM VirtualBox [22], which is compatible with a number of host operating systems, including Linux, Windows, and Mac OS. For detailed installation instructions, visit our home page [21]. The source code is available as open source via the GitHub repository.

### Experimental setup and data acquisition

KREAP was designed to perform single-cell segmentation and therefore, nuclear labeling using live-cell compatible dyes or stably transduced cell lines expressing fluorescent nuclear markers is necessary to ensure accuracy of the image analysis pipeline. We also encourage scientists to verify the normal response of transduced or fluorescently labeled cells to specific stimuli (see Supplementary Fig. S1). Importantly, the automatic recognition of the scratch boundaries as well as the quantitative determination of the re-epithelialization kinetics implemented in KREAP require standardization of the scratch's shape and size in order to minimize variation between wells. This can be accomplished by using dedicated high-throughput scratching tools such as the HTScratcher (Peira, BE; also see Methods) or defined cell-free gap inserts like the ones supplied by ibidi (ibidi, DE). The KREAP toolbox can process horizontal scratches or cell-free areas where only one scratch edge or both edges are visible in the field of view (see Supplementary Fig. S2). Furthermore, vertical or diagonal scratches can be processed in the KREAP workflow provided that the user indicates in the index file the number of degrees needed to rotate the images (clockwise) to obtain horizontal scratches. Images of the same field of view must be acquired at fixed intervals until the scratches in the wells treated with a positive control are fully resolved.

### Analysis workflow and data handling

**Figure 1:**
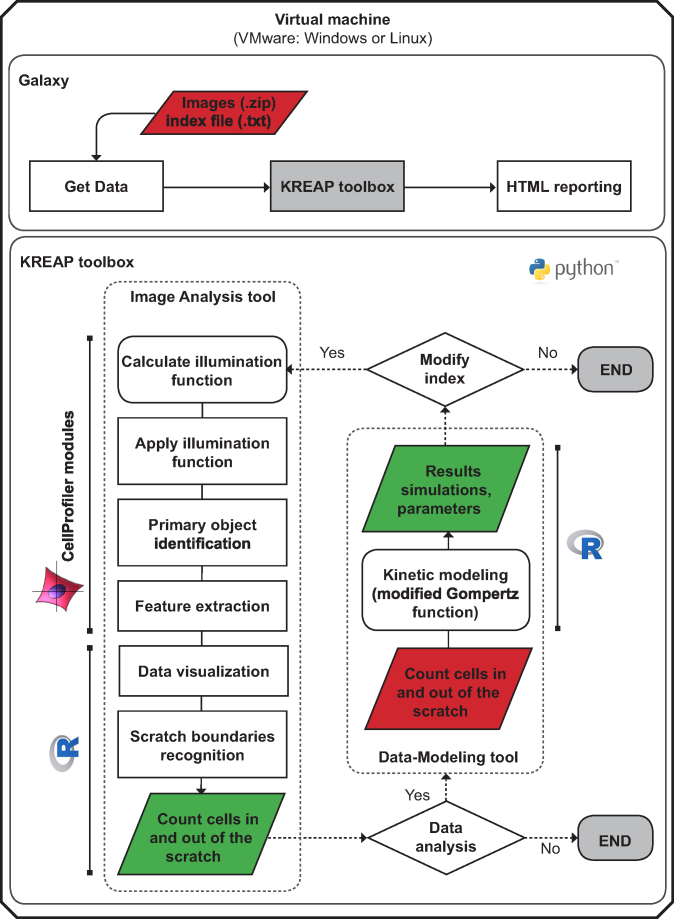
KREAP workflow. The virtual machine contains the KREAP toolbox and uses the graphical user interface provided by Galaxy, including HTML reporting. The KREAP toolbox consists of Image Analysis and Data-Modeling tools. Logos indicate the use of specialized (open-source) software or programming environments in different stages of data processing. Red parallelograms indicate input, and green parallelograms indicate output. Python was used to integrate the non-Galaxy applications into Galaxy tools.

Images (.tif) derived from a multiwell plate experiment must be converted into grayscale, organized in consecutive order, placed in folders by well, and indexed accordingly in a separate file. An exemplary index (.txt) and input files are provided at the KREAP home page [21]. The folders containing the image series of each well should be compressed into a .zip file and uploaded into the Galaxy history via the “Get data” tool together with its corresponding index file (Fig. 1). The KREAP toolbox, consisting of the “Image Analysis” and “Data-Modeling” tools, can be executed within the Galaxy platform. At the end of each processing step, the results are provided as HTML and stored in the Galaxy history for future consultation. If desired, the graphs (.png) and tables (.txt) generated by both tools can be downloaded as a compressed file (.zip) by clicking the *Download* icon in the Galaxy history. When necessary (e.g. when there is a technical error), it is possible to exclude specific wells from the analyses by clicking the link “Make new index file,” without having to upload the modified data files into the Galaxy history.

### Image analysis tool

Once the input files are uploaded into the Galaxy history, the Image Analysis tool can be executed (Fig. 1). The tool uses an image segmentation pipeline developed in the open-source software CellProfiler 2.2.0 [19]. The individual modules contained in the pipeline carry out automated extraction of cellular features in every image. An illumination function is calculated in the first segmentation module by finding the minimum pixel intensities in blocks (e.g. block size 5–20 pixels) across each image and applying a Gaussian filter as smoothing method [26]. In the second module, the calculated illumination function is applied to the raw image by subtraction, resulting in better contrast between the fluorescent nuclei and the background. Identification of primary objects is defined in the third module as objects within a specified diameter range (in pixels) depending on the cell type used. Primary objects are identified by applying a global threshold strategy in combination with the Otsu algorithm [27], which calculates a single threshold value that classifies pixels above the threshold as foreground and below the threshold as background. Because objects tend to be brighter toward the interior than toward the edges, a watershed algorithm is used to separate merged objects into individual ones [28]. The last module extracts phenotypic features (e.g. size, eccentricity, and mean intensity) from each object as well as their *x*- and *y*-coordinates within the image. For optimal image segmentation results, the user can adjust the parameters for illumination correction (i.e. block size) and object identification (i.e. minimum and maximum object diameter size) for each well directly in the index file. However, for objective comparison, it is recommended that the same parameters be used across wells seeded with the same cell type.

**Figure 2:**
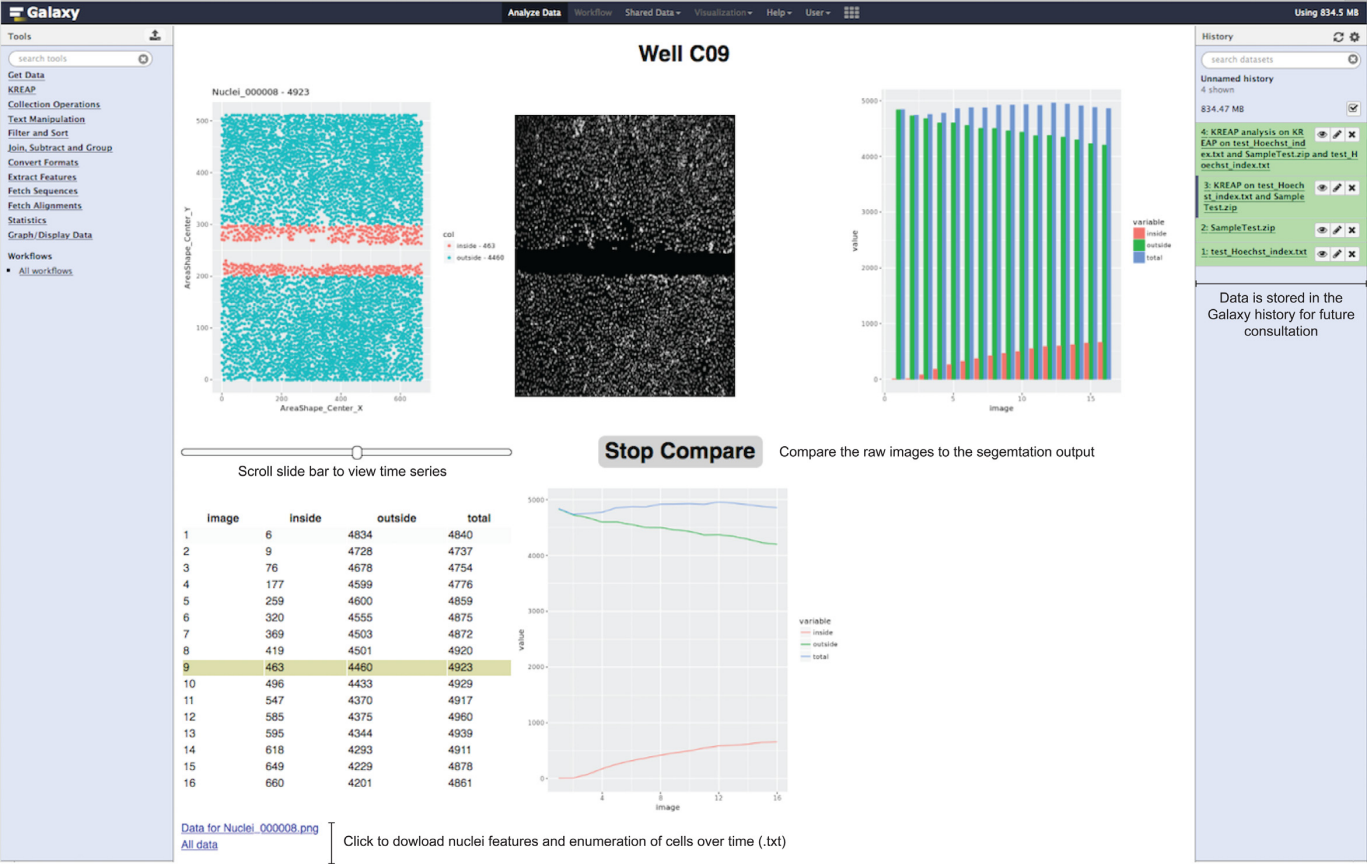
KREAP Image Analysis tool graphical output example. Image segmentation output can be easily compared with the raw images. Automatic recognition of scratch boundaries enables the enumeration of nuclei inside and outside of the scratched area over time.

The graphical interface of Galaxy provides the user with an overview of each well within the multiwell plate (Fig.2). The location of the identified primary objects is visualized in an interactive plot that uses a slider to move through images over time. A compare function is provided to visually evaluate the performance of the image segmentation pipeline by comparing its output with the raw image. Automatic identification of the scratch boundaries was accomplished using a customized R script that detects the largest cell-free area in each well by measuring the cell frequency on the *y*-axis at the beginning of the assay (see Supplementary Fig. S3 for a schematic view). To avoid underestimation of the scratch size that could result if single cells are left within the scratched area, the algorithm searches for smaller gaps above and below of the largest cell-free area. When smaller gaps are identified, these are added up to the largest cell-free area, resulting in the final identification of the scratch boundaries. The total number of cells is determined in each image over time and classified into objects inside or outside of the scratched area (Fig.2). The image segmentation results are stored in the Galaxy history and can be accessed by the user in the future. Furthermore, the cellular features extracted by CellProfiler and the enumeration of cells inside or outside of the scratched area can be easily downloaded via the links provided in the output.

### Data-Modeling tool

**Figure 3:**
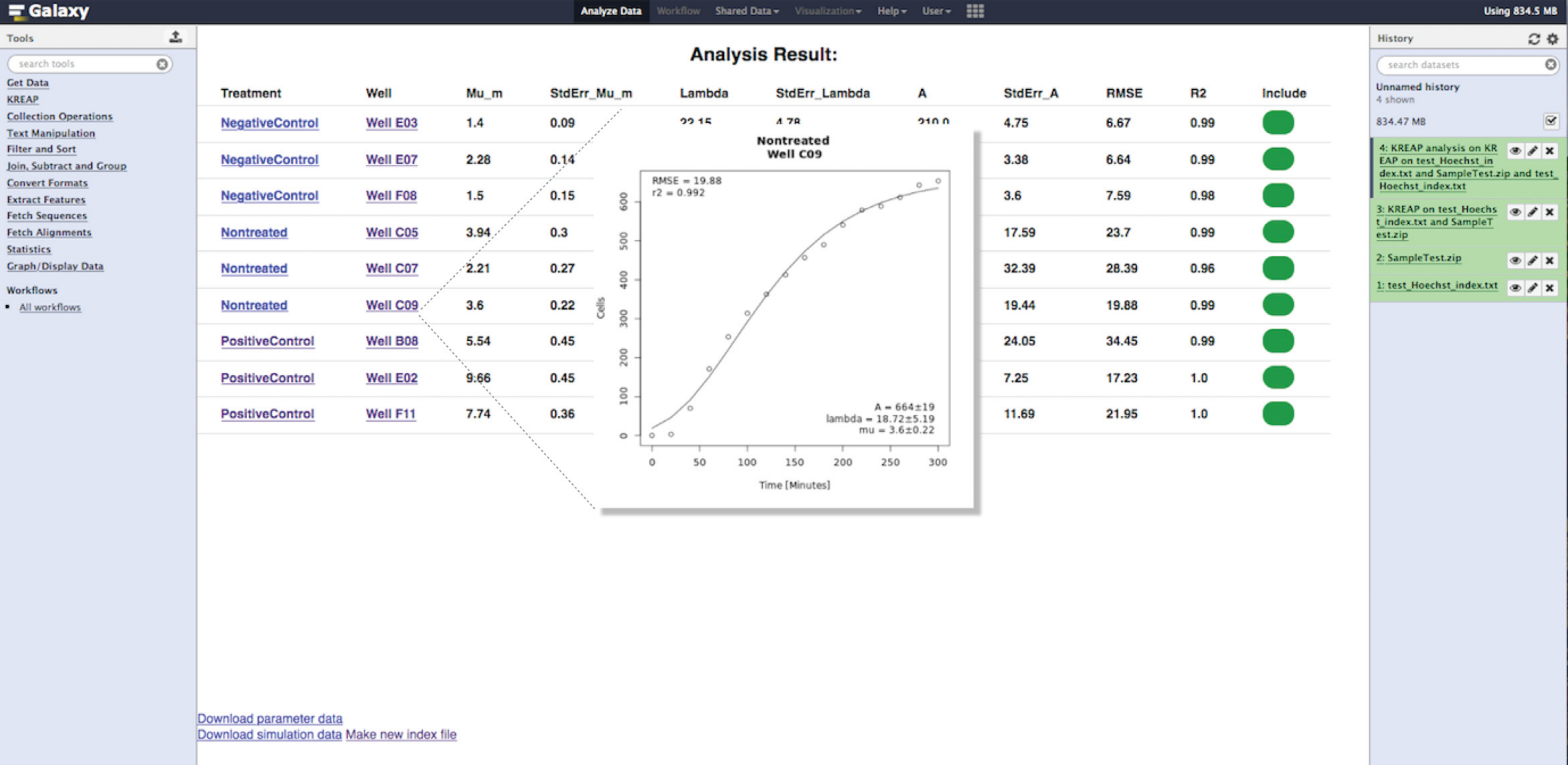
KREAP Data-Modeling tool output example. Re-epithelialization kinetics are described by the estimation of the λ, μ_m_, and A parameter values. The parameter values, simulation data, and re-epithelialization curves per replicate are provided in an HTML report and can be downloaded through the available links.

The output derived from the image analysis can be used in the Data-Modeling tool to extract three biologically relevant parameters that describe the kinetics of re-epithelialization (Fig.1). To calculate the parameter values, the time interval between images must be entered in the index file before uploading the file into the Galaxy history. The enumeration of cells infiltrating the scratched area over time consistently results in a sigmoidal curve similar to the ones obtained with bacterial growth curves that are characterized by a lag phase, an exponential phase, and a stationary phase (Fig. 3) [18]. The modified Gompertz function has been successfully used to model bacterial growth and estimate three biologically relevant parameters that mathematically describe the different phases of growth [29]. We developed and implemented an R script to fit the modified Gompertz function through the re-epithelialization measurements using a nonlinear least squares regression in combination with the Levenberg-Marquardt algorithm to reduce the sum of the squares of the errors between the modeled and measured data points in an iterative manner [18]. In this way, we were able to obtain excellent fits that were characterized by R^2^ values close to 1 and low root-mean-square error (RMSE) values. The modified Gompertz function describes the re-epithelialization kinetics for each image series through the estimation of the lag time (λ; minutes), the repair rate (μ_m_; cells minute^−1^), and the maximum number of cells within the scratched area at the plateau of the re-epithelialization curve (A; number of cells). The λ parameter represents the time required for cells to start migrating into the scratched area. For some cell lines (e.g. Ca9-22), the lag time can be very brief and the migration process may start even before image acquisition takes place [18]. In those cases, the λ parameter is estimated to be zero or may have negative values in which case, the biological relevance of this parameter to the kinetic description is negligible. Nonetheless, the calculation of the λ parameter remains essential for obtaining an accurate fit of the model. The μ_m_ parameter is an indicator of the repair rate (cells min^−1^), whereas the A parameter gives an indication of the “status” of wound closure. To this end, the A parameter is correlated to the classic measurement of scratch assays that use monolayer advancement to calculate the maximum wound closure achieved under a particular treatment after a certain period of time [18]. The parameter values obtained for each replicate condition can be used in a screening to identify substances that stimulate or attenuate wound repair when compared with the nontreated controls.

### Anticipated results

**Figure 4:**
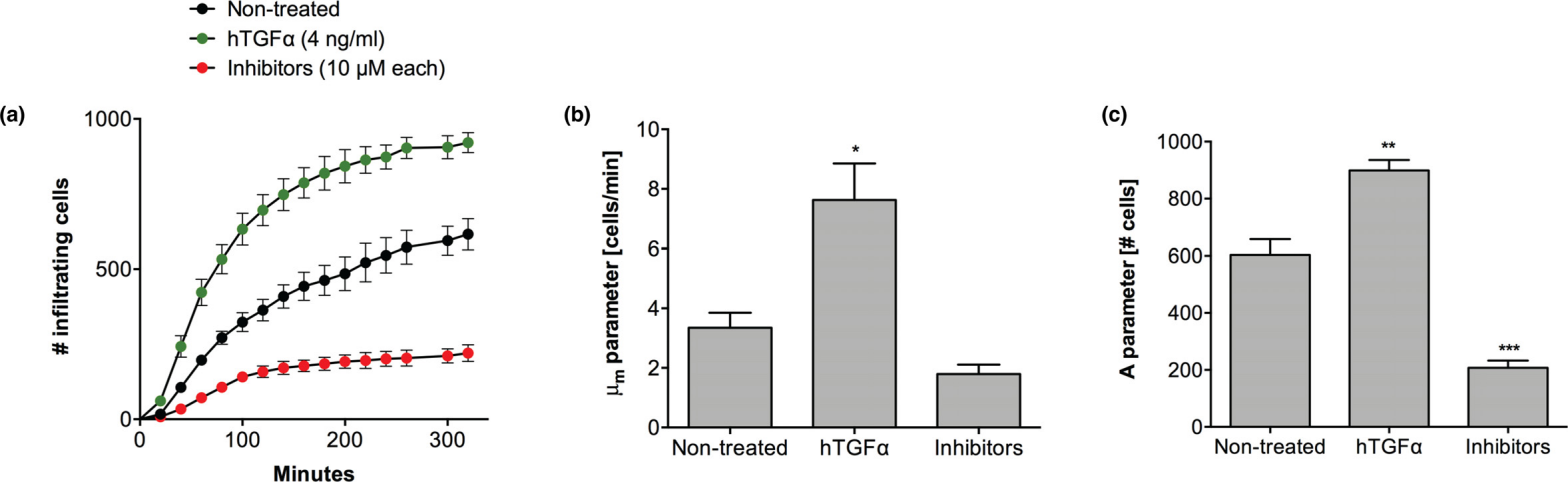
Exemplary results obtained with the KREAP toolbox. **(a)** Enumeration of single cells infiltrating the scratched area over time. Ca9-22 cells were treated with hTGFα (4 ng/ mL), a solution containing p38, and MEK1/2 inhibitors (10 µM each) or left untreated. **(b)** Comparison of the repair rate (μ_m_ parameter) obtained with the different treatments. **(c)** Comparison of the maximum numbers of infiltrating cells (A parameter) obtained with the different treatments. Significant differences from the nontreated control were assessed using a one-way analysis of variance using a Dunnett's test for multiple comparisons (n = 3; *, *P* < 0.05; **, *P* < 0.01; ***, *P* < 0.001).

As an exemplary dataset, gingival epithelial cells (Ca9-22) were seeded in 96-well plates and incubated overnight to obtain a confluent cell monolayer. During the last 20 minutes of the starvation period (i.e. incubation with fetal calf serum [FCS]-free Dulbecco's Modified Eagle's Medium [DMEM] for 2 hours), nuclei were stained with 2 μg/ mL Hoechst 33342. After 2 hours of starvation, the cell monolayers were scratched with the HTSScratcher to create an artificial wound in each well. The wells were then washed twice with phosphate-buffered saline (PBS) to remove the nuclear staining solution and detached cells. Human transforming growth factor α (hTGFα) acted as a mitogenic and mobility factor (Fig.4a) through the activation of the epidermal growth factor receptor [30]. In contrast, addition of chemical inhibitors of p38 and MEK1/2 phosphorylation led to suppression of cell migration and ERK1/2-mediated proliferation (Fig. 4a), respectively [31, 32]. Calculation of the kinetic parameters describing re-epithelialization kinetics showed that treatment with hTGFα resulted in more than a 2-fold increase in the repair rate (*P =* 0.015) when compared with the untreated cells (Fig. 4b). Likewise, stimulation with hTGFα resulted in a 1.5-fold increase in the number of cells inside the scratched area in comparison with the nontreated control (*P =* 0.004) (Fig. 4c). Inversely, treatment with the solution containing p38 and MEK1/2 inhibitors resulted in a 2- and 3-fold decrease in repair rate and in the number of infiltrating cells (*P* = 0.0008), respectively, when compared to the nontreated control during the scratch assay ( Figs.4b and 4c).

### Validation of the KREAP toolbox with a published dataset

**Figure 5:**
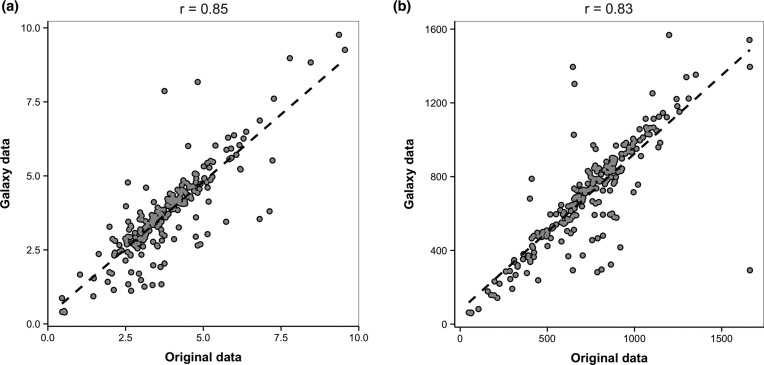
Correlation between the parameter values originated with a multi-software approach and the KREAP toolbox. **(a)** Repair rate (μ_m_ parameter, cells/ minute) and **(b)** maximum number of cells (A parameter, cells). The correlation between the parameter values was evaluated using Pearson correlation analysis (n = 214); for both cases, a positive and significant correlation was found (*P* < 0.0001).

The performance of the KREAP toolbox was evaluated in comparison with our original published workflow in which we used multiple software tools and manual determination of the scratch boundaries [18]. Using the KREAP toolbox in the Galaxy platform, we processed a dataset from that study consisting of 214 image series. Data processing was performed on a Windows 7 desktop computer with an Intel Core™ i7-3970X processor with four cores at 3.50 GHz and 4 GB of random access memory. Both KREAP and the original workflow use CellProfiler for automated image segmentation and feature extraction. However, in the original workflow, data visualization and enumeration of infiltrating cells over time was determined using the licensed FCS Express 4 Plus (De Novo Software, CA, USA) software tool. The location of the identified objects at the beginning of the assay was plotted in a scatterplot after which a rectangular gate was manually placed on the scratched area and a batch process was set up to record the number of infiltrating cells over time for each well [18]. FCS Express 4 Plus was replaced in the KREAP Image Analysis tool by an in-house developed and customized R script that automatically recognizes the boundaries of the scratch. To assess the performance of the KREAP Image Analysis tool in comparison with the manual determination of the scratched area used in our original study [18], the kinetic parameter values (i.e. μ_m_ and A) obtained with both workflows were compared using a Pearson correlation analysis. For both the μ_m_ and A parameters, the analysis identified a strong correlation between the values obtained in the previous study and those obtained with the KREAP toolbox with correlation values of 0.85 (*P* < 0.0001) and 0.83 (*P* < 0.0001), respectively (Fig. 5). These results illustrate the accuracy of the KREAP toolbox, which eliminates user's manual data-handling but also significantly reduces the time required for performing the analysis. For example, processing of a multiwell plate experiment consisting of 60 wells and 16 timepoints (960 images in total) with the original workflow would typically take around 3 to 4 hours for an experienced user to complete. In contrast, the KREAP toolbox can perform the complete analysis—from raw images to quantification of re-epithelialization kinetics—in less than 30 minutes with the additional advantage that it does not require computer programming skills.

### Identification of adverse effects on re-epithelialization

**Figure 6:**
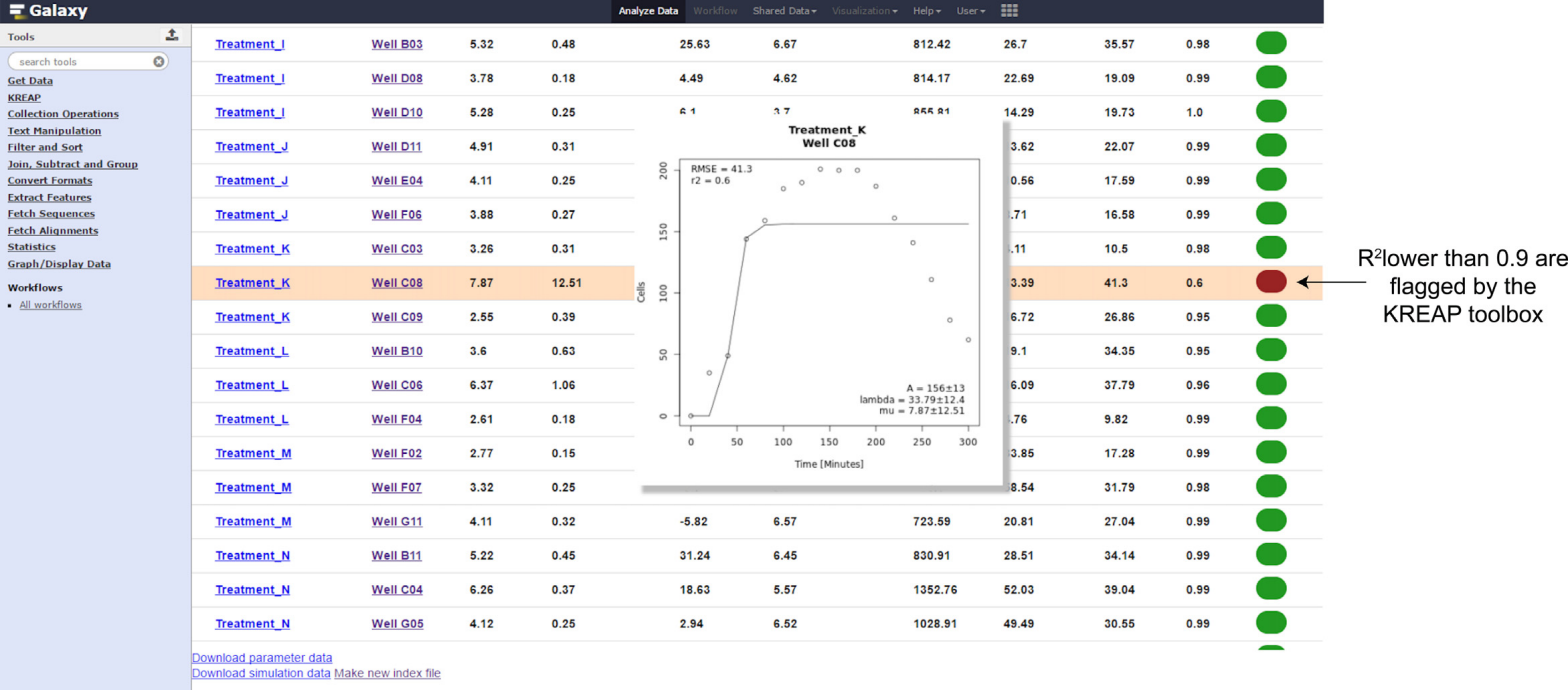
Identification of adverse effects on re-epithelialization. Adverse effects on re-epithelialization are characterized by a low R^2^ value as a result of extensive cell death after reaching the plateau of the growth curve.

Although the modified Gompertz function is used to model positive sigmoidal growth curves, identification of adverse effects on re-epithelialization kinetics is possible through the inspection of the curves generated with the measured and modeled data points. In this example (Fig.6), Ca9-22 cells were exposed to the periodontal pathogen, *Porphyromonas gingivalis*, which adversely affects re-epithelialization [18, 33, 34]. As indicated by the high repair rate value obtained (μ_m_ = 7.9 cells/ minute), the gingival cells migrated rapidly into the scratched area shortly after exposure to this bacterium. However, after reaching a plateau at 150 minutes, exposure to this bacterium led to induction of cell death and, subsequently, a decline in the number of infiltrated cells over time, resulting in a low A parameter value and an unresolved wound. Modeling of the re-epithelialization curve resulted in a poor goodness of fit (R^2^ = 0.66) that could be easily recognized by the KREAP Data-Modeling flagging system, which highlights curves with R^2^ values lower than 0.9 to be inspected by the user.

### Robustness of the KREAP toolbox and final remarks

A key aspect of high-throughput microscopy research is to convert the raw images into quantitative and biologically comprehensive data. This step typically requires multiple software tools, programming skills, or purchase of costly software to aid in the image processing and data analysis. The KREAP toolbox integrates multiple validated tools in Galaxy that enable automatic image segmentation, visualization, and data analysis of high-throughput screenings using scratch assays. The implementation of the KREAP toolbox in Galaxy also provides an open-source web-based platform that allows scientists who lack advanced programming skills to perform the complete analysis, starting with the raw images and ending with the quantified kinetics based on single-cell recognition. Moreover, the graphical user interface of Galaxy provides an easy-to-use environment that organizes, displays, and saves the results of each experiment as part of the user's history.

To further demonstrate the robustness and versatility of the KREAP toolbox, we tested it with images that were generously provided by Dr. Ng and Dr. Brugge (Harvard Medical School, Boston, MA, USA) and that were part of a collective migration study [35]. We show that the KREAP toolbox can be used to process images containing vertical scratches by indicating in the index file that the images should be rotated by 90°. Furthermore, we were able to validate the performance of the Image Analysis tool using a different cell line (MCF10A) that expresses H2B-mCherry as a nuclear marker, and with images in which only one edge of the scratch was visible in the field of view (Supplementary Fig. S2). The Data Modeling tool yielded an excellent fit (R^2^ = 0.998) with a very low RMSE (12.93), indicating a high accuracy in the estimation of the parameter values describing re-epithelialization (Supplementary Fig. S2).

Taken together, the KREAP toolbox in Galaxy provides an “end-to-end” integrated high-throughput screening platform that is useful for scientists who are interested in the discovery and/or mechanistic analysis of compounds that can modulate re-epithelialization kinetics.

## Methods

### Cell line

Gingival epithelial cells (Ca9-22) were purchased from the National Institute of Biomedical Innovation JCRB Cell Bank, Osaka (JCRB cat. no. JCRB0625, RRID:CVCL_1102). Ca9-22 cells were cultured in DMEM containing Glutamax (Gibco, Invitrogen, Paisley, UK), 10% FCS, 100 U/ mL penicillin, and 100 μg /mL streptomycin (Sigma-Aldrich, MO, USA). Cells were cultured at 37°C in a humidified atmosphere containing 5% CO_2_ and passaged when a 70% confluency was reached.

### Scratch assay and image acquisition

The experiment was carried out as described in [18]. Briefly, Ca9-22 cells were seeded in 96-well plates (BD Falcon, Corning, NY, USA) at a density of 3.5 × 10^4^ cells/well and incubated over night to obtain a confluent cell monolayer. The next day, cells were starved in FCS-free DMEM for 2 hours to decrease basal cell proliferation. During the last 20 minutes of starvation, nuclei were stained with FCS-free DMEM containing 2 μg/ mL Hoechst 33342. Following starvation, equally sized scratches (0.3 × 2 mm) were introduced in the cell monolayers with the HTSScratcher (Peira, Antwerpen, BE). After washing the cells twice with PBS, treatments (for details see [18]) were added into the wells in a randomized manner using three technical replicates. The positive control consisted of 4 ng/ mL hTGFα (R&D Systems, MN, USA). A combination of inhibitors of p38 (SB203580; Cell Signaling Technology, MA, USA) and MEK1/2 (U0126, Cell Signaling Technology) at a concentration of 10 µM each served as the negative control. FCS-free DMEM was used as nontreated control. The overall quality of each run of the 96-well based assay was assessed by calculation of the Z' factor, which establishes a dynamic range between the positive and negative control values [36]. Images were acquired using the BD Pathway 855 Bioimaging System (BD Biosciences, CA, USA) under controlled temperature and atmospheric conditions (37°C and 5% CO_2_). Fluorescent images were acquired using an excitation filter of 350 nm. The BD Pathway platform was programmed to acquire the same field of each well every 20 minutes for 5 hours using a 4× objective (40x magnification).

## Availability and requirements

- Project name: KREAP (Kinetic Re-Epithelialization Analysis Pipeline)
- Project home page: https://erasmusmc-bioinformatics.github.io/KREAP/
- Operating system: KREAP was developed in Linux and can be executed in Unix-based operating systems, Microsoft Windows or Mac OS X.
- Programming languages: Python, R programming language
- License: Freely available under the MIT open source license
- Any restriction to use as non-academic: none
- Virtual machine accessibility: via the KREAP homepage and GitHub repository.

## Supplementary Material

GIGA-D-17-00209_Original_Submission.pdf

GIGA-D-17-00209_Revision_1.pdf

GIGA-D-17-00209_Revision_2.pdf

Response_to_Reviewer_Comments_Original_Submission.pdf

Response_to_Reviewer_Comments_Revision_1.pdf

Reviewer_1_Original_Submission_Attachment_giga-science.pdf

Reviewer_1_Report_(Original_Submission) -- Assaf Zaritsky 9/24/2017 Reviewed

Reviewer_1_Report_(Revision_1) -- Assaf Zaritsky 3/6/2018 Reviewed

Reviewer_1_Revision_1_Attachment_giga-science.pdf

Reviewer_2_Report_(Original_Submission).pdf

Additional Files

## Availability of supporting data

The dataset supporting the results of this article is available at the KREAP homepage and in the *GigaScience* GigaDB repository [37].

## Additional files

**Figure S1**. Comparison of the re-epithelialization kinetics obtained with fluorescently stained and unstained cells.

**Figure S2**. Validation of the KREAP toolbox with a published image series.

**Figure S3**. Schematic view of the automatic recognition of the scratch boundaries performed by KREAP.

## Abbreviations

DMEM: Dulbecco's Modified Eagle's Medium; hTGFα: human transforming growth factor α; KREAP: kinetic re-epithelialization analysis pipeline; PBS: phosphate-buffered saline; RMSE: root-mean-square error; VM: virtual machine
