# Supplementary material for: KREAP: an automated Galaxy platform to quantify in vitro re-epithelialization kinetics
Source: Gigascience. 2018 Jun 28;7(7):giy078. doi: 10.1093/gigascience/giy078 (PMC6048990; doi:10.1093/gigascience/giy078)
Supplement: GIGA-D-17-00209_Original_Submission.pdf [file giy078_giga-d-17-00209_original_submission.pdf]

GigaScience

KREAP: An automated Galaxy Platform to Quantify Re-Epithelialization Kinetics

--Manuscript Draft--

**Manuscript Number:**	GIGA-D-17-00209
**Full Title:**	KREAP: An automated Galaxy Platform to Quantify Re-Epithelialization Kinetics
**Article Type:**	Technical Note
**Funding Information:**	
**Abstract:**	Background: In vitro scratch assays have been widely used to study the influence of bioactive substances on the processes of cell migration and proliferation that are involved in re-epithelialization. The development of high-throughput microscopy and image analysis has enabled scratch assays to become compatible with high-throughput research. However, effective processing and in-depth analysing of such high-throughput image-datasets is far from trivial and requires integration of multiple image processing and data extraction software tools.<br><br>Findings: We developed and implemented a Kinetic Re-Epithelialization Analysis Pipeline (KREAP) in Galaxy. The KREAP toolbox automatically performs image segmentation and feature extraction of each image series, followed by automatic quantification of cells inside and outside the scratched area over time. The enumeration of infiltrating cells over time is modelled to extract three biological relevant parameters that describe re-epithelialization kinetics. The output of the tools is organized, displayed, and saved in the Galaxy environment for future consultation. Importantly, a Pearson correlation analysis confirmed a strong association between the values obtained using a multi-software approach with those obtained with the KREAP toolbox.<br><br>Conclusions: The Galaxy KREAP toolbox provides an open-source easy-to-use web-based platform for reproducible image processing and data analysis of high-throughput scratch assays. The KREAP toolbox could assist a broad scientific community in the discovery of compounds that are able to modulate re-epithelialization kinetics.
**Corresponding Author:**	Andrew Stubbs<br><br>NETHERLANDS
**Corresponding Author Secondary Information:**	
**Corresponding Author's Institution:**	
**Corresponding Author's Secondary Institution:**	
**First Author:**	Marcela M. Fernandez-Gutierrez
**First Author Secondary Information:**	
**Order of Authors:**	Marcela M. Fernandez-Gutierrez
	David B.H. van Zessen
	Peter van Baarlen
	Kleerebezem Michiel
	Andrew P. Stubbs
**Order of Authors Secondary Information:**	
**Opposed Reviewers:**	
**Additional Information:**	
**Question**	**Response**

Powered by Editorial Manager® and ProduXion Manager® from Aries Systems Corporation

Are you submitting this manuscript to a special series or article collection?	No
**Experimental design and statistics**<br><br>Full details of the experimental design and statistical methods used should be given in the Methods section, as detailed in our Minimum Standards Reporting Checklist. Information essential to interpreting the data presented should be made available in the figure legends.<br><br>Have you included all the information requested in your manuscript?	Yes
**Resources**<br><br>A description of all resources used, including antibodies, cell lines, animals and software tools, with enough information to allow them to be uniquely identified, should be included in the Methods section. Authors are strongly encouraged to cite Research Resource Identifiers (RRIDs) for antibodies, model organisms and tools, where possible.<br><br>Have you included the information requested as detailed in our Minimum Standards Reporting Checklist?	Yes
**Availability of data and materials**<br><br>All datasets and code on which the conclusions of the paper rely must be either included in your submission or deposited in publicly available repositories (where available and ethically appropriate), referencing such data using a unique identifier in the references and in the "Availability of Data and Materials" section of your manuscript.<br><br>Have you have met the above requirement as detailed in our Minimum Standards Reporting Checklist?	Yes

Powered by Editorial Manager® and ProduXion Manager® from Aries Systems Corporation

Manuscript

Click here to download Manuscript KREAP-An automated Galaxy Platform.docx

KREAP: An automated Galaxy Platform to Quantify Re-Epithelialization Kinetics

Marcela M. Fernandez-Gutierrez[1,2$], David B.H. van Zessen[2$], Peter van Baarlen[2], Michiel Kleerebezem[1,2], Andrew P. Stubbs[3*]

[1]TI Food and Nutrition, Nieuwe Kanaal 9-A, 6709 PA, Wageningen, The Netherlands.

[2]Host-Microbe Interactomics, Animal Sciences Group, Wageningen University & Research, De Elst 1, 6708 WD, Wageningen, The Netherlands.

[3]Department of Bioinformatics, Erasmus University Medical Centre, Wytemaweg 80, 3015 CN, Rotterdam, The Netherlands.

$ - Both authors contributed equally

*** - Correspondence**

Andrew P. Stubbs: a.stubbs@erasmusmc.nl

Author email addresses:

Marcela M. Fernandez-Gutierrez: marcela.fernandez@wur.nl

David B.H. van Zessen: d.vanzessen@erasmusmc.nl

Peter van Baarlen: peter.vanbaarlen@wur.nl

Michiel Kleerebezem: michiel.kleerebezem@wur.nl

Abstract

Background: *In vitro* scratch assays have been widely used to study the influence of bioactive substances on the processes of cell migration and proliferation that are involved in re-epithelialization. The development of high-throughput microscopy and image analysis has enabled scratch assays to become compatible with high-throughput research. However, effective processing and in-depth analysing of such high-throughput image-datasets is far from trivial and requires integration of multiple image processing and data extraction software tools.

Findings: We developed and implemented a Kinetic Re-Epithelialization Analysis Pipeline (KREAP) in Galaxy. The KREAP toolbox automatically performs image segmentation and feature extraction of each image series, followed by automatic quantification of cells inside and outside the scratched area over time. The enumeration of infiltrating cells over time is modelled to extract three biological relevant parameters that describe re-epithelialization kinetics. The output of the tools is organized, displayed, and saved in the Galaxy environment for future consultation. Importantly, a Pearson correlation analysis confirmed a strong association between the values obtained using a multi-software approach with those obtained with the KREAP toolbox.

Conclusions: The Galaxy KREAP toolbox provides an open-source easy-to-use web-based platform for reproducible image processing and data analysis of high-throughput scratch assays. The KREAP toolbox could assist a broad scientific community in the discovery of compounds that are able to modulate re-epithelialization kinetics.

Keywords: Galaxy, scratch assay, high-throughput, cell migration, re-epithelialization, image analysis, workflow, modelling.

Findings

Background

Cell migration and proliferation play an essential role in a variety of physiological processes, including embryogenesis, angiogenesis, skin and intestinal renewal, and wound repair [1, 2]. Deregulation of these processes can contribute to the development and progression of multiple diseases such as osteoporosis, rheumatoid arthritis, vascular disease and cancer [1]. Therefore, studying the molecular mechanisms underlying the processes of cell migration and proliferation is not only important for obtaining fundamental scientific insight, but it is also essential for the development of effective therapeutic strategies that could modulate these processes.

The *in vitro* scratch assay is a well-established and widely used method to study cell migration and proliferation [3-5]. The assay is based on the introduction of a scratch into a confluent epithelial

cell monolayer to create a “wounded” area. Cells migrate and proliferate into the site of injury in a process known as re-epithelialization [6]. This process is typically monitored by acquisition of images at the beginning and at one or more fixed time points during re-epithelialization. The image series of a particular treatment are then compared to those of the non-treated control to determine the treatment’s modulatory capacity in the healing process. The development and constant improvement of image segmentation algorithms over the past decades has enabled the transition from manual quantification of the scratch area to automated analysis that is compatible with high-throughput research [7-9].

CellProfiler [10], ImageJ [11] and TScratch [12] are freely available image analysis software tools that allow scientists with limited programming skills to conduct efficient image segmentation and feature extraction. Nevertheless, data analysis of scratch assays has been traditionally limited to the quantification of percentage of wound closure during the course of the experiment [3], neglecting the kinetic information inherent of the re-epithelialization process (e.g. repair rate) and hampering the possibility of single cell migration analysis. Moreover, to optimally use the different capacities of the abovementioned software tools, scripting and parsing of data are often necessary, which require programming skills that many biologists do not have. Software such as FCS Express Image Cytometry [13] and WimScratch [14] provide alternatives to ease data analysis, however the former requires the purchase of a license and the latter requires payment per image analysed. Thus there is a need in the scientific community with an interest in studying the processes of cell migration and proliferation for an open-source workflow platform that integrates different validated tools for image segmentation, visualization and data analysis.

We developed and implemented a Kinetic Re-Epithelialization Analysis Pipeline (KREAP) in Galaxy (https://galaxyproject.org/) [15] to deliver a web browser based application for the quantitative analysis of *in vitro* scratch assays. The user only need to download a virtual machine (VM) containing a fully operational KREAP Galaxy installation, upload the images from a multi-well plate experiment into the VM, and press the *Execute* button to automatically perform single cell

segmentation and feature extraction in all images. Enumeration of cells inside and outside the scratch is also carried out automatically over the time series. The number of cells infiltrating the scratch over time is employed by KREAP to extract three biological comprehensive parameters that describe the kinetics of re-epithelialization. In addition, the user's history is saved in the VM for future consultation and the results can be easily shared with other users. Taken together, we provide a platform that enables reproducible data processing and analysis of high-throughput scratch assays: from raw images to re-epithelialization kinetics.

Implementation

The scratch assay analysis workflow was developed within our own laboratory [16] and involves a multi-software approach to acquire images, perform image analysis, visualize extracted data, and model re-epithelialization kinetics. CellProfiler 2.1.1 (http://cellprofiler.org/) was used in the original workflow and implemented in the KREAP toolbox to perform automated image segmentation and feature extraction of the image series. FCS Express 4 Plus (De Novo Software, CA, USA) was originally used to relate the features extracted by CellProfiler back to the raw images and to enumerate the cells infiltrating the scratched area over time. Since FCS Express 4 Plus requires the purchase of a license, we developed and implemented an R [17] script in the KREAP toolbox that can automatically recognize the scratch boundaries and determine the number of cells inside and outside the scratch over time. Modelling of re-epithelialization kinetics was programmed in R and also implemented into the KREAP toolbox workflow. The workflow is provided in a fully operational Galaxy installation inside of a VM that can be retrieved from the GitHub repository and run in Microsoft Windows or Linux using the freely available VMware Workstation Player (http://www.vmware.com). Alternatively, Mac OS X users can run the VM using VirtualBox (https://www.virtualbox.org/wiki/Downloads). The source code is available as open-source via the GitHub repository.

Analyses workflow and data handling

For accurate image segmentation, nucleic acids must be labelled with a fluorescent marker (e.g. Hoechst 33342, SYTO® dyes, etc.) and image acquisition should be performed with a 4X or 5X objective to obtain a complete view of the scratched area and surrounding cells [16]. Images (.tif) derived from a multi-well plate must be converted into grayscale, organized in folders by well, and indexed accordingly in a separate file (.txt). An exemplary index and input files are provided in the GitHub repository (https://erasmusmc-bioinformatics.github.io/KREAP/file_formats). The folders containing the image series of each well are compressed into a .zip file, which is uploaded into the Galaxy history via the "Get data" tool together with its corresponding index file (**Fig. 1**). The KREAP toolbox, consisting of the "Image Analysis" and "Data-Modelling" tools, can be executed within the Galaxy platform. At the end of each processing step, the results are provided as HTML and stored in the Galaxy history for future consultation. If desired, the graphs (.png) and tables (.txt) generated by both tools can be downloaded for all wells as a .zip file by clicking the *Save* icon in the Galaxy history.

Image Analysis tool

Once the input files are uploaded into the Galaxy history, the "Image Analysis" tool can be executed (**Fig. 1**). The tool uses an image segmentation pipeline developed in the open-source software CellProfiler 2.1.1 (http://cellprofiler.org/). The individual modules contained in the pipeline carry out automated extraction of cellular features in every image. An illumination function is calculated in the first segmentation module by finding the minimum pixel intensities in blocks (recommended size: 10-20 pixels) across each image and applying a Gaussian filter [18] as smoothing method. In the second module, the calculated illumination function is applied to the raw image by subtraction, resulting in better contrast between the fluorescently labelled nuclei and their background. Identification of primary objects is defined in the third module as nuclei ranging typically between 4 to 15 pixels in diameter. Identification of primary objects is performed by applying a global threshold strategy in combination with the Otsu algorithm [19] which calculates a single threshold value that classifies pixels above the threshold as foreground and

below the threshold as background. Objects tend to be brighter towards the interior than towards the edges. The difference in intensity was then used to separate merged objects into individual ones. The last module extracts phenotypic features (e.g. size, eccentricity and mean intensity) from each object as well as their x- and y-coordinates within the image. For optimal image segmentation results, the user can adjust the parameters for illumination correction (i.e., block size) and object identification (i.e., minimum and maximum object diameter size) per well directly in the index file. However, it is important to remark that for an objective comparison, it is recommendable to use the same parameters across wells seeded with the same cell type.

The graphical interface of Galaxy provides the user with an overview of each well within the multi-well plate (**Fig. 2**). The location of the identified primary objects is visualized in an interactive plot that uses a slider to move through images over time. A "compare" function is provided to visually evaluate the performance of the image segmentation pipeline by comparing its output with the raw image. Automatic identification of the scratch boundaries was programmed in R by finding the largest distance between cells and then expanding up and down looking for smaller gaps to avoid incorrect determination of the scratch boundaries. The total number of objects (i.e., nuclei or cells) is enumerated in each image over time and classified into objects inside or outside of the scratched area (**Fig. 2**). The image segmentation results are stored in the Galaxy history and can be accessed by the user in the future. Furthermore, the cellular features extracted by CellProfiler and the enumeration of objects inside or outside of the scratched area can be easily downloaded through the provided links.

Data-Modelling tool

The output derived from the "Image Analysis" can be used in the "Data-Modelling" tool to extract biologically relevant parameters that describe the kinetics of re-epithelialization (**Fig. 1**). To calculate the parameter values, the time interval between images must be entered in the index file (see https://erasmusmc-bioinformatics.github.io/KREAP/file_formats). The enumeration of

infiltrating cells into the scratched area over time consistently results in a sigmoidal curve (**Fig. 3**), which can be successfully modelled using the modified Gompertz function [20]. Thus we developed and implemented an R script that uses a nonlinear least squares regression to fit this function through the re-epithelialization measurements [16]. Furthermore, the Levenberg-Marquardt algorithm [21] was used in this script to reduce the sum of the squares of the errors between the modelled and measured data points in an iterative manner. In this way, we were able to obtain excellent and accurate fits, which were characterised by R^2 values close to 1 and low root-mean-square error (RMSE) values. The modified Gompertz function describes the re-epithelialization kinetics for each image series by three biologically relevant parameters: lag time (λ in minutes), repair rate (μ_m cells minute^{-1}), and maximum number of cells reached within the scratched area at the plateau phase of the growth curve (A). The λ parameter represents the time in minutes required for the cells to start migrating into the scratched area. For some cell lines (e.g., Ca9-22), the lag time can be very brief and the migration process may start even before image acquisition takes place according to the model outcome [16]. In those cases, the λ parameter would be estimated to be zero or even have negative values and thus, the biological contribution of this parameter to the description of re-epithelialization kinetics would be limited, but its calculation essential for obtaining a good fit of the model. Apart from the lag time or λ parameter, the μ_m and the A parameters can be used to compare the re-epithelialization kinetics of cells treated with a specific substance to that of the non-treated control. The combinatorial approach of incorporating the μ_m and the A parameters into a single performance value (μ_m*A) can be useful to identify potential stimulators and attenuators of re-epithelialization. For this calculation, the μ_m and the A values of each treatment are normalized against the corresponding average values of the non-treated control [16].

Validation with published sub-data set

To validate the performance of KREAP in comparison with the original workflow that uses a multi-software approach, we re-analysed a sub-data set of one of our previous studies using the KREAP

toolbox in the Galaxy platform. Data processing was performed on a Windows desktop with an Intel® Core™ i7-3970X processor with 4 cores at 3.50 GHz and 4 GB of RAM. Both KREAP and the original workflow make use of CellProfiler for automated image segmentation and feature extraction. Data visualization and enumeration of infiltrating cells over time was determined previously using the commercially available FCS Express 4 Plus (De Novo Software, CA, USA) software tool, which we now replaced by a freely available R script [16]. The location of the identified objects at the beginning of the assay was plotted in a scatterplot after which a gate was manually placed on the scratched area and a batch process was setup to record the number of infiltrating cells over time for each well [16]. Processing of a multi-well plate experiment consisting of 60 wells and 16 time points (960 images in total) would typically take around 3 to 4 hours for an experienced user to complete with this protocol. In addition, programming skills are required to extract the parameter values that described re-epithelialization kinetics. In contrast, the KREAP toolbox can automatically perform the complete analysis from raw images to quantification of re-epithelialization kinetics in less than 30 minutes. Furthermore, a Pearson correlation analysis confirmed the association between the values obtained in the previous study with those obtained with the KREAP toolbox for both the μ_m and A parameters with R^2 values of 0.87 and 0.83 respectively (**Fig. 4**).

Identification of detrimental effects on re-epithelialization and troubleshooting

The modified Gompertz function models growth (i.e., positive sigmoidal curves), but identification of detrimental effects on re-epithelialization kinetics is still possible through inspection of the curves generated with the measured and modelled data points. In the first example (**Fig. 5a**), cells had migrated into the scratched area until the plateau was reached, but this was followed by a reduction in the number of infiltrating cells over time as a result of cell death, leading to a low R^2 value. The KREAP "Data-Modelling" tool flags R^2 values lower than 0.9 to be inspected by the user. In the second example (**Fig. 5b**), cell migration was strongly inhibited by the treatment and therefore, only very few cells migrated into the scratched area. These

curves cannot be modelled by the modified Gompertz function and therefore, the parameter values describing re-epithelialization kinetics will not be retrieved. The KREAP "Data-Modelling" tool flags the wells in which the parameter values could not be retrieved and the user can decide to exclude the treatment from the analysis by clicking the icon in the *Include* column and have a new index file automatically created through the provided link (**Fig. 5c**). Finally, if the assay is too short for a particular treatment to reach the plateau phase of the growth curve (**Fig. 5d**), the estimation of the parameter values would be overestimated, resulting in outliers. In this case, the user is advised to extend the timeframe of the assay.

Conclusions

A key aspect of high-throughput microscopy research is to convert the raw images into quantitative and biologically comprehensive data. This step often requires multiple software tools, programming skills or purchase of costly software to aid in the image processing and data analysis. The KREAP toolbox integrates multiple validated tools for image segmentation, visualization and data analysis of high-throughput scratch assays in Galaxy. Our KREAP toolbox in Galaxy provides an open-source web-based platform that enables scientists that lack sophisticated programming skills to perform the complete analysis starting with the raw images and ending with the quantified kinetics. Furthermore, the graphical user interface of Galaxy provides an easy-to-use environment that organizes, displays, and saves the results of every experiment as part of the user history. This is the first open-source application to provide an "end to end" integrated analytical high-throughput screening platform that is useful for scientists who are interested in the discovery and mechanistic analysis of compounds that can modulate re-epithelialization kinetics.

Availability and Requirements

- Project name: KREAP (Kinetic Re-Epithelialization Analysis Pipeline)
- Project home page: https://erasmusmc-bioinformatics.github.io/KREAP/

- Operating system: Unix-based Operating Systems
- Programming languages: Python, R programming language
- License: Free
- Any restriction to use as non-academic: none
- Virtual machine accessibility: via the GitHub repository

Availability of supporting data

The source code and supporting data for the presented analyses are publicly available in the project home page.

Competing interests

The authors declare there that they have no competing interests.

Authors' contributions

MMFG, DHBvZ, PvB, MK and AS conceived the study and contributed to writing the first draft of the manuscript. MMFG performed the scratch assays and carried out the validation analyses. DHBvZ and AS developed and implemented the analyses workflow. All authors contributed to editing the final manuscript. All authors read and approved the final manuscript.

References

1. Ridley AJ, Schwartz MA, Burridge K, Firtel RA, Ginsberg MH, Borisy G, et al. Cell Migration: Integrating Signals from Front to Back. Science. 2003;302(5651):1704-9. doi: 10.1126/science.1092053.
2. Friedl P, Gilmour D. Collective cell migration in morphogenesis, regeneration and cancer. Nat Rev Mol Cell Biol. 2009;10(7):445-57.
3. Liang CC, Park AY, Guan JL. In vitro scratch assay: a convenient and inexpensive method for analysis of cell migration in vitro. Nature protocols. 2007;2(2):329-33. doi: 10.1038/nprot.2007.30. PubMed PMID: 17406593.
4. Oudhoff MJ, Van Den Keijbus PAM, Kroeze KL, Nazmi K, Gibbs S, Bolscher JGM, et al. Histatins enhance wound closure with oral and non-oral cells. J Dent Res. 2009;88(9):846-50. doi: 10.1177/0022034509342951.
5. Mohammedsaeed W, Cruickshank S, McBain AJ, O'Neill CA. Lactobacillus rhamnosus GG Lysate Increases Re-Epithelialization of Keratinocyte Scratch Assays by Promoting Migration. Scientific Reports. 2015;5:16147. doi: 10.1038/srep16147. http://www.nature.com/articles/srep16147 - supplementary-information.
6. Schäfer M, Werner S. Transcriptional Control of Wound Repair. Annual Review of Cell and Developmental Biology. 2007;23(1):69-92. doi: 10.1146/annurev.cellbio.23.090506.123609.
7. Yarrow JC, Perlman ZE, Westwood NJ, Mitchison TJ. A high-throughput cell migration assay using scratch wound healing, a comparison of image-based readout methods. BMC biotechnology. 2004;4:21. doi: 10.1186/1472-6750-4-21. PubMed PMID: 15357872; PubMed Central PMCID: PMC521074.
8. Zordan MD, Mill CP, Riese DJ, 2nd, Leary JF. A high throughput, interactive imaging, bright-field wound healing assay. Cytometry Part A : the journal of the International Society for Analytical Cytology. 2011;79(3):227-32. Epub 2011/11/03. doi: 10.1002/cyto.a.21029. PubMed PMID: 22045642; PubMed Central PMCID: PMC3306835.

9. Simpson KJ, Selfors LM, Bui J, Reynolds A, Leake D, Khvorova A, et al. Identification of genes that regulate epithelial cell migration using an siRNA screening approach. Nat Cell Biol. 2008;10(9):1027-38. doi: http://www.nature.com/ncb/journal/v10/n9/suppinfo/ncb1762_S1.html.
10. Lamprecht MR, Sabatini DM, Carpenter AE. CellProfiler™: Free, versatile software for automated biological image analysis. BioTechniques. 2007;42(1):71-5. doi: 10.2144/000112257.
11. Schindelin J, Rueden CT, Hiner MC, Eliceiri KW. The ImageJ ecosystem: An open platform for biomedical image analysis. Molecular Reproduction and Development. 2015;82(7-8):518-29. doi: 10.1002/mrd.22489.
12. Geback T, Schulz MM, Koumoutsakos P, Detmar M. TScratch: a novel and simple software tool for automated analysis of monolayer wound healing assays. BioTechniques. 2009;46(4):265-74. Epub 2009/05/20. doi: 10.2144/000113083. PubMed PMID: 19450233.
13. Software DN. A Flow Cytometry Analysis Environment for Image Cytometry Data. Available from: https://www.denovosoftware.com/site/Image-Overview.shtml. 6 ed.
14. Wimasis. WimScratch: Wound Healing Assay Image Analysis Solution. Availabe from: https://www.wimasis.com/en/products/9/WimScratch. 4.0 ed2016.
15. Goecks J, Nekrutenko A, Taylor J. Galaxy: a comprehensive approach for supporting accessible, reproducible, and transparent computational research in the life sciences. Genome Biology. 2010;11(8):R86-R. doi: 10.1186/gb-2010-11-8-r86. PubMed PMID: PMC2945788.
16. Fernandez-Gutierrez MM, Roosjen PPJ, Ultee E, Agelink M, Vervoort J, Keijser B, et al. Streptococcus salivarius MS-oral-D6 promotes gingival re-epithelialization in vitro through a secreted serine protease. Manuscript submitted for publication.
17. R Development Core Team. R: A Language and Environment for Statistical Computing. Vienna, Austria: R Foundation for Statistical Computing; 2011.
18. Lindblad J, Bengtsson E, editors. A comparison of methods for estimation of intensity nonuniformities in 2D and 3D microscope images of fluorescence stained cells. Proceedings of the 12th Scandinavian Conference on Image Analysis (SCIA); 2001.
19. Otsu N. A Threshold Selection Method from Gray-Level Histograms. IEEE Transactions on Systems, Man, and Cybernetics. 1979;9(1):62-6. doi: 10.1109/TSMC.1979.4310076.
20. Zwietering MH, Jongenburger I, Rombouts FM, van 't Riet K. Modeling of the Bacterial Growth Curve. Applied and Environmental Microbiology. 1990;56(6):1875-81.
21. Elzhov TV, Mullen KM, Spiess A-N, Bolker B. minpack.lm: R Interface to the Levenberg-Marquardt Nonlinear Least-squares Algorithm Found in MINPACK, Plus Support for Bounds. R package version 1.2-1. https://cran.r-project.org/package=minpack.lm. 2016.

Figure legends

Figure 1. KREAP workflow. The virtual machine contains the KREAP toolbox and uses the graphical user interface (GUI) provided by Galaxy, including HTML reporting. The KREAP toolbox consists of the "Image Analysis" and "Data-Modelling" tools. Logos indicate the use of specialized (open source) software or programming environments in different stages of the data processing. Red parallelograms indicate input and green parallelograms indicate output. Python was used to integrate the non-Galaxy applications into Galaxy tools.

Figure 2. KREAP "Image Analysis" graphical output example. Image segmentation output can be easily compared with the raw image. Automatic recognition of scratch boundaries enables the enumeration of nuclei inside and outside of the scratched area over time.

Figure 3. KREAP "Data-Modelling" tool output example. Re-epithelialization kinetics described by the λ, μ_m and A parameters. The parameter values, simulation data and re-epithelialization curves per replicate are provided in an HTML report and are also available for download through the available links.

Figure 4. Association between the parameter values originated with multi-software approach and the KREAP toolbox. (a) Repair rate (μ_m parameter, cells minute^{-1}) and (b) Maximum number of cells (A parameter, cells). The association of the parameter values was evaluated by Pearson correlation analysis (n = 215) and for both cases a positive and significant correlation was found ($P < 0.0001$).

Figure 5. Identification of detrimental effects and outliers using KREAP. (a) Detrimental effect on re-epithelialization characterized by a low R^2 value as a result of extensive cell death after reaching the plateau of the growth curve. (b) Strong inhibitory effect results in migration of very few cells into the scratched area and as a result the parameter values that describe re-epithelialization cannot be retrieved. (c) The user can decide to exclude certain wells of the analysis by pressing the icon in the *Include* column. (d) Overestimation of the parameter values can result from growth curves that do not reached the plateau phase in the timeframe of the assay.

Figure-1

Click here to download Figure Figure-1.pdf

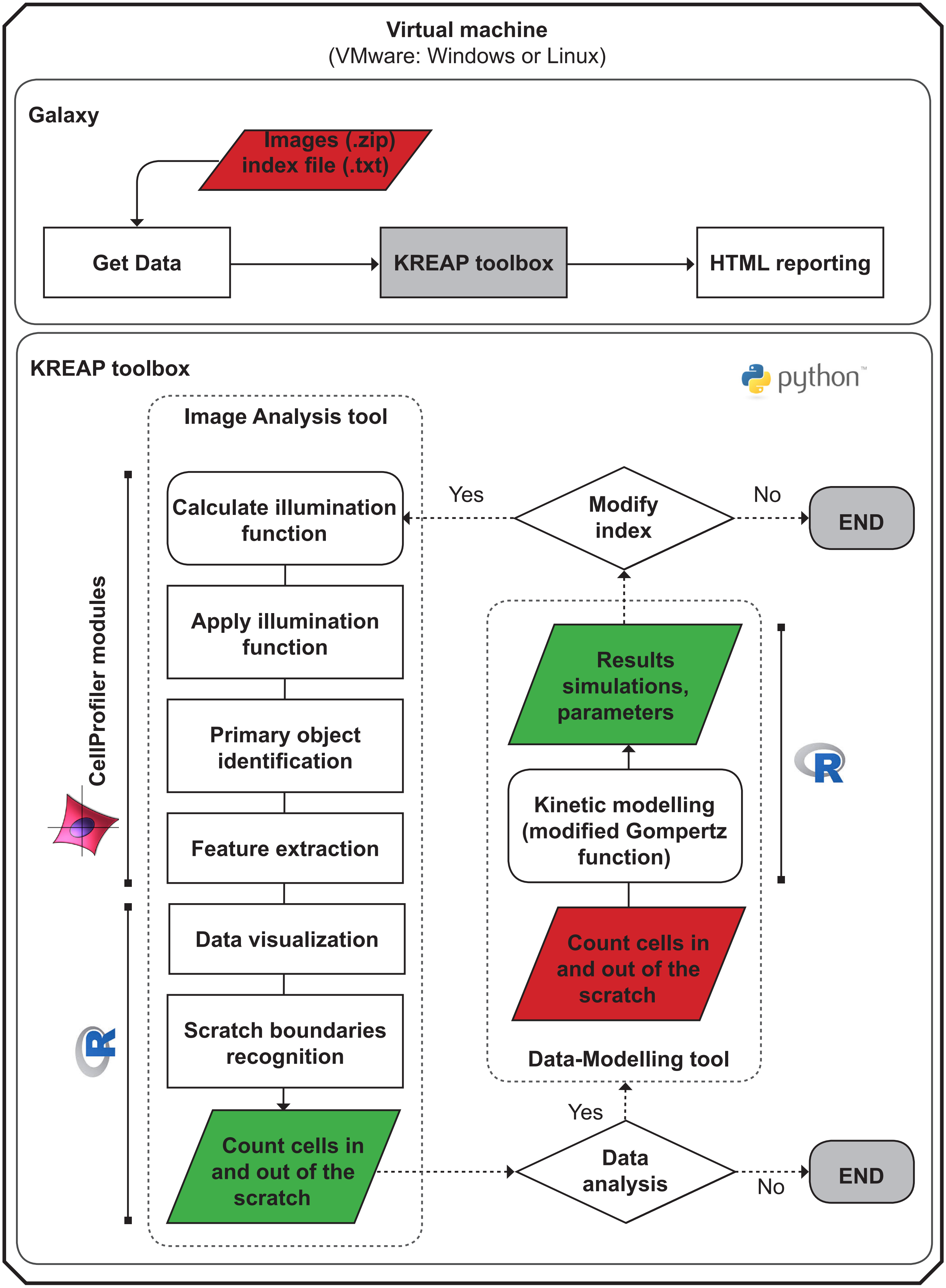

Figure-2

Click here to download Figure Figure-2.pdf

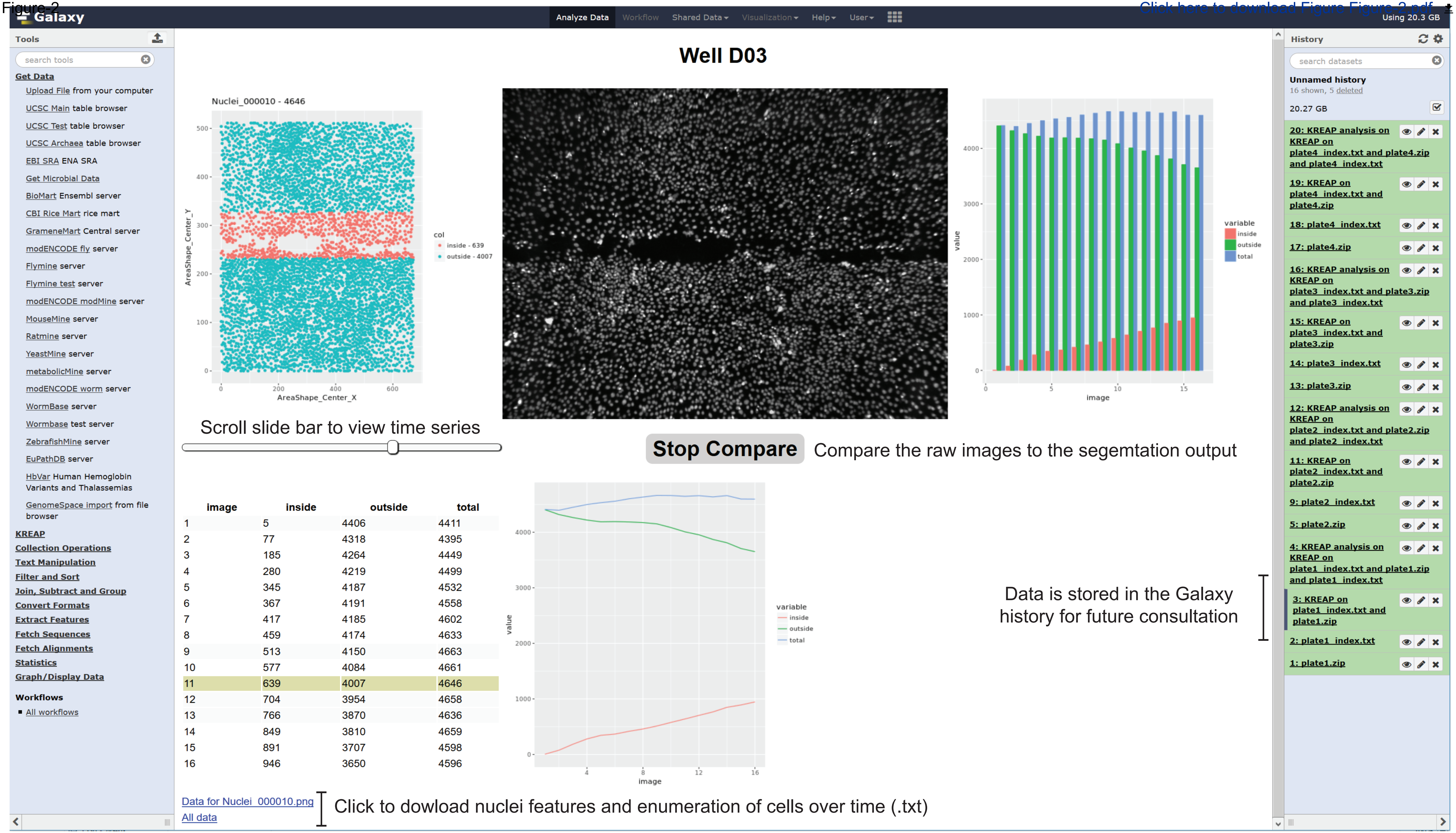

Figure-3

Click here to download Figure Figure-3.pdf

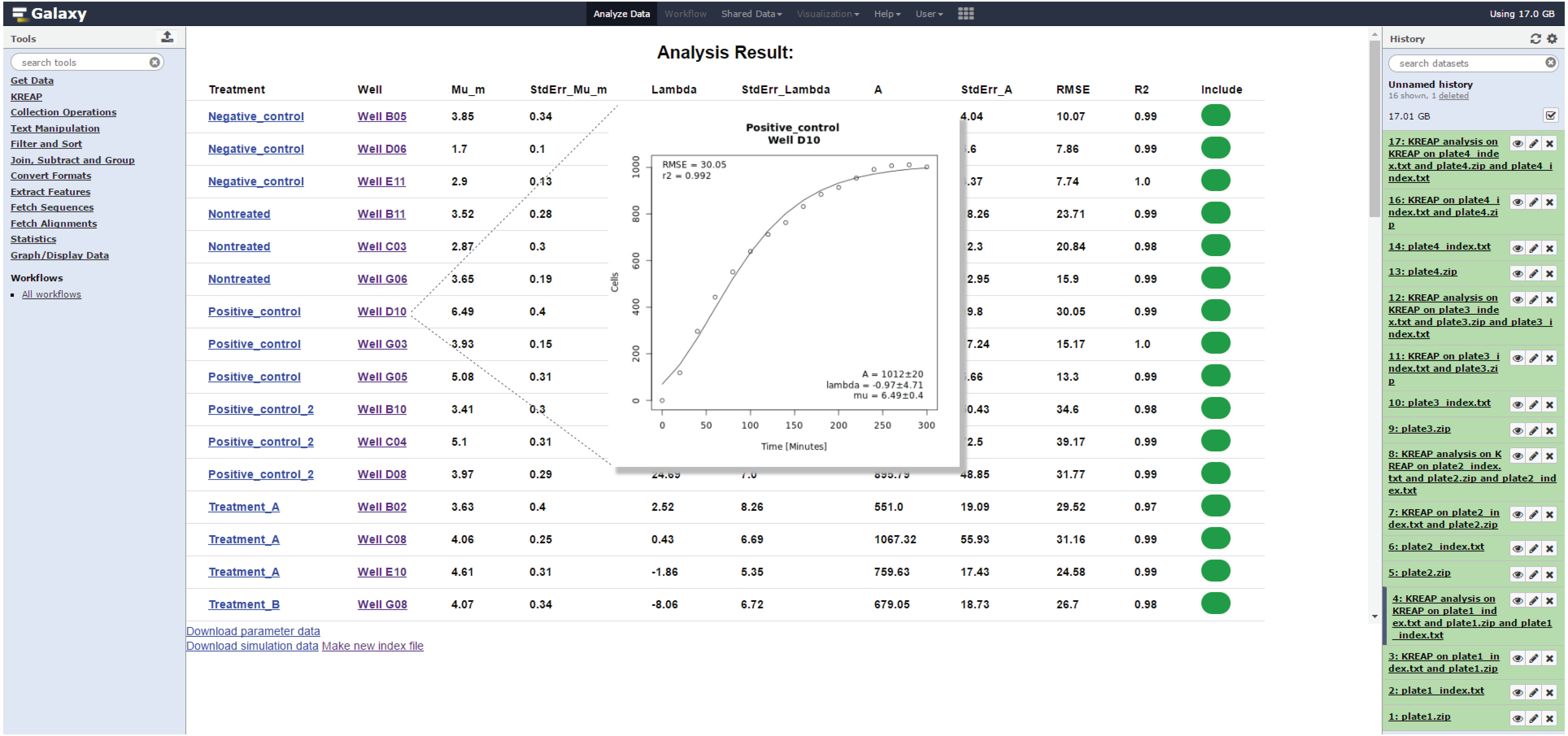

Figure-4

Click here to download Figure Figure-4.pdf

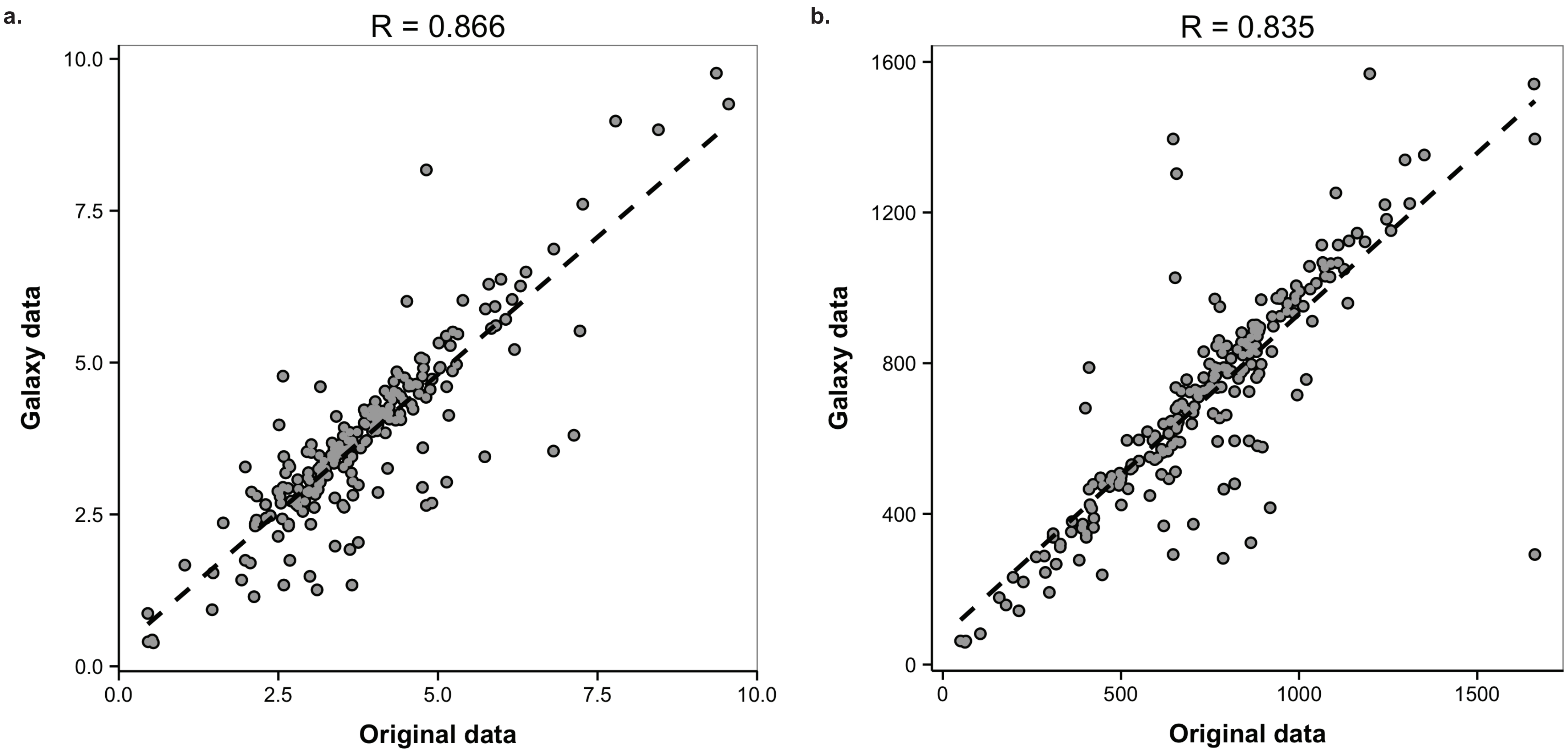

Figure-5

Click here to download Figure Figure-5.pdf

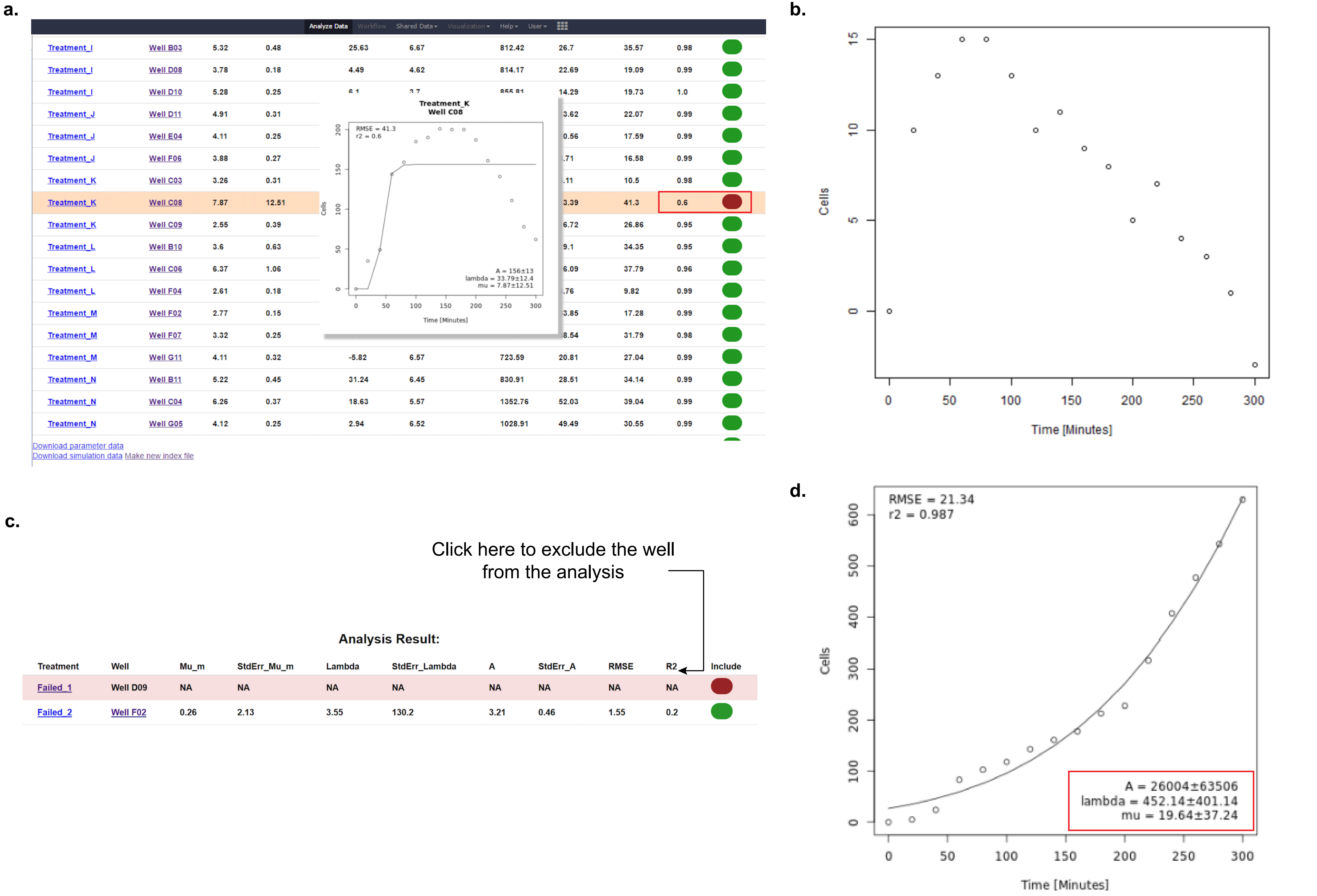

Click here to access/download
Supplementary Material
Additional manuscript.pdf

Cover Letter Click here to download Personal Cover CoverLetter.pdf

Erasmus MC
Universitair Medisch Centrum Rotterdam

Clinical Bioinformatics
Department of Pathology
Andrew Stubbs, PhD

Nicole Nogoy, PhD
Editor
Gigascience,

Direct dial +31-10-704.4776
Room number Ee 15-76
E-mail a.stubbs@erasmusmc.nl
Date August 17th, 2017

Dear Editor,

We wish to submit our manuscript entitled "**KREAP: An automated Galaxy Platform to Quantify Re-epithelialization Kinetics**" by Fernandez-Gutierrez *et al.* as technical note manuscript to Gigascience.

Our aim was to develop an open source application to support the analysis of high-throughput microscopy and image analysis data from *in vitro* scratch assays commonly used to study the influence of bioactive substances on the processes of cell migration and proliferation. Data analysis of scratch assays has been limited to the quantification of percentage of wound closure during the course of the experiment, neglecting the kinetic information inherent of the re-epithelialization process. Additionally, to optimally use the different capacities of the current open-source software tools, scripting and parsing of data are often necessary, which require programming skills that many biologists do not have.

To address these challenges we developed and implemented a **K**inetic **R**e-**E**pithelialization **A**nalysis **P**ipeline (KREAP) in Galaxy which provides an "end to end" solution image processing, quantitation and visual reporting including parameters that describe re-epithelialization kinetics.

We demonstrate the utility of KREAP using a dataset from a previous study (provided as an "Additional manuscript" for reviewing purposes only) and confirm that the results obtained with KREAP correlate with kinetic parameter values obtained using a multi-software approach that requires manual intervention.

In summary KREAP is an open-source easy-to-use web-based platform for reproducible image processing and data analysis of high-throughput scratch assays available for use as a virtual machine in both large and small-scale laboratories. KREAP can be downloaded from https://erasmusmc-bioinformatics.github.io/KREAP/.

Finally, we confirm that the content of the manuscript has not been published or submitted for publication elsewhere. Furthermore, we confirmed that all the authors approved the manuscript for submission and declare that there are no competing interests.

We appreciate you considering our article for publication in Gigascience and look forward to your reply.

Yours sincerely,

Andrew Stubbs, PhD

Assistant Professor of Bioinformatics
