# Supplementary material for: KREAP: an automated Galaxy platform to quantify in vitro re-epithelialization kinetics
Source: Gigascience. 2018 Jun 28;7(7):giy078. doi: 10.1093/gigascience/giy078 (PMC6048990; doi:10.1093/gigascience/giy078)
Supplement: GIGA-D-17-00209_Revision_1.pdf [file giy078_giga-d-17-00209_revision_1.pdf]

GigaScience

KREAP: An automated Galaxy Platform to Quantify in vitro Re-Epithelialization Kinetics

--Manuscript Draft--

**Manuscript Number:**	GIGA-D-17-00209R1
**Full Title:**	KREAP: An automated Galaxy Platform to Quantify in vitro Re-Epithelialization Kinetics
**Article Type:**	Technical Note
**Funding Information:**	
**Abstract:**	Background: In vitro scratch assays have been widely used to study the influence of bioactive substances on the processes of cell migration and proliferation that are involved in re-epithelialization. The development of high-throughput microscopy and image analysis has enabled scratch assays to become compatible with high-throughput research. However, effective processing and in-depth analysing of such high-throughput image-datasets is far from trivial and requires integration of multiple image processing and data extraction software tools.<br><br>Findings: We developed and implemented a Kinetic Re-Epithelialization Analysis Pipeline (KREAP) in Galaxy. The KREAP toolbox incorporates a freely available image analysis tools and automatically performs image segmentation and feature extraction of each image series, followed by automatic quantification of cells inside and outside the scratched area over time. The enumeration of infiltrating cells over time is modelled to extract three biologically relevant parameters that describe re-epithelialization kinetics. The output of the tools is organized, displayed, and saved in the Galaxy environment for future consultation. Importantly, a Pearson correlation analysis confirmed that the results obtained using separate software tools and manual determination of the scratch boundaries were comparable to the results obtained with the fully automated KREAP toolbox.<br><br>Conclusions: The Galaxy KREAP toolbox provides an open-source, easy-to-use, web-based platform for reproducible image processing and data analysis of high-throughput scratch assays. The KREAP toolbox could assist a broad scientific community in the discovery of compounds that are able to modulate re-epithelialization kinetics.<br><br>Keywords: Galaxy, scratch assay, high-throughput, cell migration, re-epithelialization, image analysis, workflow, modelling.
**Corresponding Author:**	Andrew Stubbs<br><br>NETHERLANDS
**Corresponding Author Secondary Information:**	
**Corresponding Author's Institution:**	
**Corresponding Author's Secondary Institution:**	
**First Author:**	Marcela M. Fernandez-Gutierrez
**First Author Secondary Information:**	
**Order of Authors:**	Marcela M. Fernandez-Gutierrez
	David B.H. van Zessen
	Peter van Baarlen
	Kleerebezem Michiel
	Andrew P. Stubbs
**Order of Authors Secondary Information:**	
**Response to Reviewers:**	Dear Scott Edmunds,

Powered by Editorial Manager® and ProduXion Manager® from Aries Systems Corporation

Below the point-point response to the referees on the technical note GIGA-D-17-00209 entitled "KREAP: An automated Galaxy Platform to Quantify Re-Epithelialization Kinetics". Furthermore, we thank the reviewers for their valuable time and useful contribution.

Answers to Reviewer #1

Critical concerns:
1.KREAP is limited to live imaging of nuclei stained with fluorescent markers. Vast majority of wound healing experiments are based on phase-contrast or other imaging modalities that do not require nuclei staining with the reasoning of simplifying the experiments and less inference with the cell model. The only reason I can see to require nuclei staining in this type of experiments is to enable single cell analysis. However, the parameters λ, μm and A could be calculated using the area measurements rather than cell counts.

Author response: Although image analysis could be performed with high quality phase-contrast images (see Additional file 1), we highly recommend the use of fluorescent labels for the reasons that we mention below. In summary, the use of fluorescent dyes increases the contrast between the cell or cellular organelle of interest and the background, thus improving accuracy of counting (or tracking) cells and/or organelles. Additionally, the use of specific fluorescent dyes allows the determination of viable, dead and apoptotic cell numbers as well as the number of proliferating cells.

(1)Nowadays, the use fluorescent microscopy in combination with live-compatible and fixable dyes is becoming a standard technique in most laboratories. For example, Vitorino and Meyer (2018), "Modular control of endothelial sheet migration", used 10 g/ml wheat germ agglutinin conjugated to AlexaFluor594 for 10 minutes before scraping the cells. This dye is used to label cell membranes of living cells and therefore, get a clear delimitation of the cell boundaries for automated image analysis.
(2)The use of live-compatible dyes involves a simple and fast staining step in which you add the labelling solution into each well and wait from 10-30 minutes. After the incubation time, the scratches can be introduced into the stained cell monolayers. Importantly, we have tested the influence of Hoechst 33342 and other live-compatible dyes such as CellTracker™ Red CMTPX in comparison with non-labelled cells and we showed that the fluorescent dyes do not interfere with the normal physiology of the cells (see Additional File 1).
(3)When evaluating the effect of certain bacteria on re-epithelialization, it is highly recommended to use florescent dyes because many bacteria tend to co-aggregate, interfering with the accuracy of the image analysis if carried out using bright-field images (see Fig. 4a, lines 201-205). Hence, applying fluorescent dyes to label specific cell organelles, such as the nuclei or the cell membrane, removes such artefacts and improves the accuracy of the results.
(4)Fluorescent dyes in scratch assays are not only used for single cell tracking, but also to detect apoptotic cells or live/dead cells. For example, Nyegaard et al. (2016), "An optimized method for accurate quantification of cell migration using human small intestine cells", used SYTO-24 and propidium iodide to determine live versus dead cell counts (also see lines 76-78 for more references). This provides the opportunity to accurately determine total cell numbers and numbers of live and dead cells within the same microscopy view in one go.
(5)KREAP image analysis was designed to identify single cells and thereby, quantify the number of cells infiltrating into the scratched area over time. Fluorescent stains contribute greatly to accurately identify single cells, and nuclei are excellent markers for cells that do not contain segmented or multi-lobed nuclei such as epithelial cells.

In principle, the modified Gompertz function used by KREAP to model re-epithelialization kinetics could be used with area coverage measurements. However, we argue that single cell counting in combination with an automatic determination of the scratch boundaries is a more accurate method to quantify re-epithelialization kinetics. As shown by Nyegaard et al. (2016), "An optimized method for accurate quantification of cell migration using human small intestine cells (Fig. 2)", poorly adherent cell lines (e.g. FHs-74 int cells) detach and migrate in an uneven pattern making final wound closure determination more challenging.

Powered by Editorial Manager® and ProduXion Manager® from Aries Systems Corporation

2.The authors have not convinced me that, KREAP measures that are based on nuclei staining are more informative in describing wound healing kinetics compared to other available measures designed for phase-contrast imaging

Author response: The image analysis performed by KREAP can in principle be also conducted with bright-field images when using high-quality, high contrast (see Additional File 1). However, the use of fluorescent dyes is highly recommended (see point #1 above) to improve the accuracy of the results obtained by removing possible artefacts present in the raw bright-field images and provide additional possibilities such as determining the ratios of viable/dead cells in the same field / sample.

3.The authors have not convinced me that, KREAP implementation is robust and can be easily applied to a variety of data-sets. These are crucial in arguing the usability and importance of KREAP.

Author response: In the revised version of the manuscript we included a section with anticipated results (lines 199-218) with an exemplary dataset. In addition, the KREAP toolbox was used to re-analysed a sub-dataset (total of 215 image series) from a previous study in which we used a fundamentally different analysis pipeline that involved the manual determination of the scratch boundaries. A Pearson correlation analysis confirmed the association between the results obtained with KREAP and those obtained in the previous study (lines 219-244, Fig. 5).

4.In the past decade, quite a few measures and tools were proposed to quantify temporal (or spatio-temporal) dynamics in wound healing experiments and to manage high-content data. The authors entirely missed all this body of work. It is essential to acknowledge the relevant literature and discuss the benefits and limitations of the experimental setting and measures proposed.

Author response: In the revised version of the manuscript, we expanded the background information on current tools available for high-throughput image analysis and in specific, high-throughput scratch assays and provide an overview of their features (lines 61-76). We also recognize that other studies such as the ones carried out by Vitorino and Meyer (2008) and Zaritsky et al. (2017) have provided spatio-temporal measurements of high-throughput scratch assays. However, these studies used commercial software or in-house developed analysis pipelines that are not freely available for other scientists.

We want to stress here that the added value of the KREAP procedure is that: (1) it provides an open-source, high-throughput analysis platform; (2) it provides a fully automated data analysis workflow in Galaxy that provides a user friendly interface, including comprehensive graphical outputs, experimental history and data storage; and (3) it is designed to recognize single cells and applies a nonlinear least squares regression to invert the modified Gompertz function and thereby, estimate the values of three independent biologically relevant parameters that describe different phases of the re-epithelialization process (see point #6).

5.Is there an advantage using single nuclei data over phase-contrast? Can the authors demonstrate that additional information is encoded in the single nuclei information that cannot be quantified by segmenting cellular-vs-background at a pixel-level? I would argue that the λ, μm and A can be calculated without the single nuclei information directly from the phase-contrast segmentation. I find this concern critical in arguing a more complex experimental setting. Perhaps the information encoded in the nuclei shape, that can be measure with this approach can provide this added information? This can be tested on the data from ref. 16.

Author response: In principle, automated cell recognition can be executed on high-quality, phase-contrast images or fluorescently stained cells. However, the accuracy of the image segmentation is higher when using fluorescently labelled cell organelles, and it offers additional possibilities to explore the same sample, for instance by quantifying ratios of live and dead cells. As explained in point #1, the addition of live-compatible fluorescent dyes does not require a complex experimental setup and in most laboratories is considered a standard technique. Furthermore, the enumeration of

single cells over time results in a sigmodial curve that can be modelled by inverting the modified Gompertz function (see point #6).

6.Relevance of the extracted parameters. What information / insight is gained by using λ, μm and A? I would expect μm to be highly correlative to the wound healing rate. It is not clear to me what biological insight can be gained from A (the authors mention that the   is only important for accurate estimation of μm). Can the authors correlate the different parameters to one another and to the overall wound healing rate? Can it be demonstrated that A has a biological interpretation that is independent of μm? Can you find modular experimental conditions for these two measures?

Author response: The modified Gompertz function was originally developed to model bacterial growth curves and is described by Zwietering et. al. (1990), "Modeling of the Bacterial Growth Curve". These characteristic sigmodial growth curves consist of a lag phase, an exponential phase, and a stationary phase. As we described in ref. 19 of the revised manuscript, when we plot the number of epithelial cells that migrate into the scratch area over time, we consistently obtained sigmodial growth curves. As explained in lines 178-192 of the revised manuscript, λ represents the lag time or the amount of time (in minutes) before the cells start migrating into the scratched area. The μm parameter is an estimation of the repair rate (cells/min-1), and the A parameter is the calculation of the maximum number of cells that entered into the scratched area. As we showed in ref. 19 (Fig. 2), the A parameter correlates to the maximum surface area (i.e. pixels) covered with cells (r = 0.68, P = 1.36 x 10-6). Hence, these parameters describe different aspects of the re-epithelialization kinetics and should therefore, not be correlated to each other. These are biologically relevant parameters reflect the initial cellular responses to a bioactive substance and subsequent activation/inhibition of cell proliferation. These are insights that are relevant to clinical research, infectious diseases, cancer and toxicology, to name a few.

We are not completely sure what the reviewer means with "finding modular experimental conditions", but in Fig. 6a we provide an example in which addition of a particular treatment initially induced rapid cell migration (represented by a high m value), but during the course of the experiment, migration of cells into the scratched area ceased, resulting in a low A parameter value.

7.Applicability to diverse data-sets. To demonstrate robustness of the method and broad usability, I would like to see the authors demonstrate their software usability on an additional dataset, preferably collected by another lab. If no such dataset is easily available, then doesn't it question the need for such software?

Author response: A search on PubMed using the key words "scratch AND assay" resulted in a total of 3,575 search items, which exemplifies the usability of such a tool for the scientific community. However, due to the size of image datasets, publication reporting on scratch assays do not provide access to the raw images that could be used to test the usability of KREAP. Nonetheless the performance of KREAP was validated with a total of 215 image series obtained in a previous study (see point #3).

Essential corrections:

8.Data availability. It was not clear to me whether the full dataset presented in the author's previous manuscript (ref. 16) is available. In ref. 16 the authors state that "The datasets generated during the current study are available from the corresponding author on reasonable request". In the current Technical Note, I could not find a clear statement whether the full dataset is publicly available (and citable), and where it can be found.

Author response: The dataset in ref. 16 (ref. 19 of the revised manuscript) is available upon request, which is related to the large size of these datasets (approximately 15 GB). This is probably the reason why other publications reporting on scratch assays do not provide access to raw data or are limited to one image series. Analogously, in the present manuscript we provide exemplary image dataset as stated in lines 317-319: "The dataset supporting the results of this article is available at the KREAP homepage (Kinetic Re-Epithelialization Analysis Pipeline)". If GigaScience is willing to support storage and availability of the complete datasets as supplementary data, we are happy

Powered by Editorial Manager® and ProduXion Manager® from Aries Systems Corporation

to upload the files.

9.The project webpage https://erasmusmc-bioinformatics.github.io/KREAP/ is not linked to a GitHub page. I could not find even with direct search in GitHub homepage for KREAP. Also, could not find the source code.

Author response: The authors regret the confusion. The supporting data is available in the KREAP project homepage. Also, the code will be made available when the paper is accepted and until then the authors will provide the reviewers and anonymous account and password to login to access the code for KREAP. The source code is available for reviewers using the reviewer login details supplied by the authors at: https://github.com/ErasmusMC-Bioinformatics/KREAP (username: erasmusmc-review; password: erasmusmcreview123). This login will be replaced by open access if the paper is accepted for publication.

10.Methods are not described in clear and sufficient manner.

a.The description of the λ, μm and A assumes that readers are familiar with the Gompertz function. Brief explicit description of the Gompertz function will make it more clear.

Author response: We expanded the explanation of the modified Gompertz function in lines 168-192 to have a more clear overview of each of the parameters estimated by the model as well as their biological explanation.

b.# 139-141: "Automatic identification of the scratch boundaries was programmed in R by finding the largest distance between cells and then expanding up and down looking for smaller gaps to avoid incorrect determination of the scratch boundaries". These steps are very vague, please be more explicit and explain systematically how this step is performed (I did not find sufficient explanation in ref. 16).

Author response: In the revised version of the manuscript we change this statement to make it less vague and more explicit (lines 157-159).

c.From briefly reading ref. 16 I did not find direct and clear description regarding how the screening scores were calculated from the multiple replicates per condition and negative/positive controls.

Author response: Ref. 16 (ref. 19 of the revised manuscript) reports on much more than the initial screening scores and describes how negative and positive scores were identified. However, in the current manuscript we provide a section with anticipated results and their interpretation (Fig. 4, lines 199-218) as well as the methods used to execute the experiment (lines 281-307).

11.I find hard to understand how to interpret the "performance value" measure, μm*A. Why is it a meaningful measure? What does it teach us about the wound healing kinetics more than m alone?

Author response: The μm and A represent independent biological relevant parameters. The combination of them in a single value (μm*A) provides a simple overall assessment of re-epithelialization performance under a certain treatment. See also answer to point #6 and lines 192-198.

Minor suggestions:

12.Availability and requirements:

d."Operating system": Unix-based Operating Systems" - I think this is supposed to imply on what OS can KREAP be executed on, which is not limited to Unix.

Author response: The authors regret the confusion and clarified this point in the revised version of the manuscript by stating that the source code/algorithms were developed in Linux, whereas the VM can be executed using Microsoft Windows, Linux or Mac OS X (lines 311-312).

Powered by Editorial Manager® and ProduXion Manager® from Aries Systems Corporation

e.License: replace "free" with formal license, see here,
https://opensource.org/license/category

Author response: We thank the reviewer for his remark. KREAP is freely available under the MIT open-source license as stated in line 314.

13.GigaScience focus on high-content datasets and analyses. Since the analysed dataset includes 60 movies (each with 16 timepoints), it can be marginally considered as "large scale" in the field. This would be another (not critical) argument for including another dataset in this Technical Note.

Author response: We regret we were not sufficiently clear in the previous version of the manuscript. As explained in the revised manuscript, the 60 image-series served as an example to indicate the computational time needed (lines 238-244) to obtain the quantitative results using KREAP. For validation of the KREAP performance, we re-analysed a total of 215 image-series, thereby, including additional datasets (also see point #3) from a previous study.

14.The dataset used in this Technical Note is not well described. Please describe the experiment, controls, and statistics. I am aware that these were (at least partially) described in ref. 16, but (I think that) these reporting standards are expected in GigaScience. Personally, I could not easily figure out how the controls were used together with the replicates to score hits in ref. 16.

Author response: In our opinion, the present manuscript does not intend to repeat the analyses as they were presented in ref. 16 (ref. 19 of the revised manuscript), but to provide the tools needed to quantify re-epithelialization kinetics. In the current manuscript we provide a section with anticipated results and their interpretation (Fig. 4, lines 199-218) as well as the methods used to execute the experiment (lines 289-307). We would like to emphasise here that the KREAP tool provides result feedback to enable recognition of potential problem samples or image-series that require the user's attention.

15.I do not see the point of Fig. 4 (#91-94). This is a trivial validation that the implementation replicates what was measured in ref. 16, especially since it is a paper by the same group. I would recommend excluding it or moving it to be a Supporting figure.

Author response: The workflows use fundamentally different procedures to determine the wound boundaries (lines 220-223). The original workflow used in ref. 16 (ref. 19 of the revised manuscript) depends on the manual definition of a rectangular wound for each well at the beginning of the measurements. KREAP automatically defines the wound boundaries by actual recognition of cells at the boundaries which is more accurate and less subjective than manual handling. Therefore, we consider Fig. 5 in the revised manuscript highly relevant for the verification of the accuracy of the KREAP tool and propose to maintain the figure in the revised version of the manuscript.

16.I also think that Figure 5 would be better suited as Supporting figure. I am in favour of focusing on the main aspects and leaving less important details for SI.

Author response: In our opinion, it is very important to point users of KREAP to potential issues in data analysis and interpretation that they might not be familiar with. As an additional service to users, we implemented automated flagging of such issues to ensure that these are not missed by the user. Figure 5 (Fig. 6 in the revised manuscript) illustrates examples of the troubleshooting output, which we prefer to maintain as a figure in the main manuscript.

Answers to Reviewer #2

1.I would make a discretionary revision recommendation that instead of shipping the .vmdk virtual drive it would be beneficial to utilize the .ova (Open Virtual Appliance) format for simpler, more standard distribution. A docker version would probably be well-received, too, as it's rapidly becoming the standard way to ship services like this.

Powered by Editorial Manager® and ProduXion Manager® from Aries Systems Corporation

	Author response: We have added a converted OVA to download. We agree with the reviewer that a docker would probably be well received. We will create a docker if there is sufficient demand from the research community to access KREAP using this format.<br><br>2.It would be nice for these tools to ultimately be added to the Galaxy Tool Shed for non-VM distribution, though it looks like the dependencies might be cumbersome to deal with and I understand choices made here.<br><br>Author response: The dependencies were indeed the reason why we did not add the tools to the Tool Shed. We considered adding the tools to the Tool Shed and let the server maintainer install the dependencies, but it would have been a very "non Galaxy" thing to do.<br><br>3.It'd be more convenient to add an additional tool in Galaxy that can consume a collection to generate this file, or perhaps modify the initial image analysis tool itself to support collections, in addition to the pre-bundled zip format.<br><br>Author response: We looked at collections, but we decided against using them because we would not be able to add the metadata (i.e. time, pixel_block_size, etc) KREAP needs without making the tool interface much more complicated. The ZIP approach allowed us to limit this complexity to an Excel sheet and organizing and zipping a directory, things most scientists are already familiar with.
**Additional Information:**	
**Question**	**Response**
Are you submitting this manuscript to a special series or article collection?	No
**Experimental design and statistics**<br><br>Full details of the experimental design and statistical methods used should be given in the Methods section, as detailed in our Minimum Standards Reporting Checklist. Information essential to interpreting the data presented should be made available in the figure legends.<br><br>Have you included all the information requested in your manuscript?	Yes
**Resources**<br><br>A description of all resources used, including antibodies, cell lines, animals and software tools, with enough information to allow them to be uniquely identified, should be included in the Methods section. Authors are strongly encouraged to cite Research Resource Identifiers (RRIDs) for antibodies, model organisms and tools, where possible.<br><br>Have you included the information requested as detailed in our Minimum Standards Reporting Checklist?	Yes

**Availability of data and materials**<br><br>All datasets and code on which the conclusions of the paper rely must be either included in your submission or deposited in publicly available repositories (where available and ethically appropriate), referencing such data using a unique identifier in the references and in the "Availability of Data and Materials" section of your manuscript.<br><br>Have you have met the above requirement as detailed in our Minimum Standards Reporting Checklist?	Yes

Powered by Editorial Manager® and ProduXion Manager® from Aries Systems Corporation

Manuscript

Click here to download Manuscript KREAP-An automated Galaxy Platform_revised.docx

KREAP: An automated Galaxy Platform to Quantify *in vitro* Re-Epithelialization Kinetics

Marcela M. Fernandez-Gutierrez[1,2$], David B.H. van Zessen[3$], Peter van Baarlen[2], Michiel Kleerebezem[1,2], Andrew P. Stubbs[3*]

[1]TI Food and Nutrition, Nieuwe Kanaal 9-A, 6709 PA, Wageningen, The Netherlands.

[2]Host-Microbe Interactomics, Animal Sciences Group, Wageningen University & Research, De Elst 1, 6708 WD, Wageningen, The Netherlands.

[3]Department of Bioinformatics, Erasmus University Medical Centre, Wytemaweg 80, 3015 CN, Rotterdam, The Netherlands.

$ - Both authors contributed equally

*** - Correspondence**

Andrew P. Stubbs: a.stubbs@erasmusmc.nl

Author email addresses:

Marcela M. Fernandez-Gutierrez: marcela.fernandez@wur.nl

David B.H. van Zessen: d.vanzessen@erasmusmc.nl

Peter van Baarlen: peter.vanbaarlen@wur.nl

Michiel Kleerebezem: michiel.kleerebezem@wur.nl

Abstract

Background: *In vitro* scratch assays have been widely used to study the influence of bioactive substances on the processes of cell migration and proliferation that are involved in re-epithelialization. The development of high-throughput microscopy and image analysis has enabled scratch assays to become compatible with high-throughput research. However, effective processing and in-depth analysing of such high-throughput image-datasets is far from trivial and requires integration of multiple image processing and data extraction software tools.

Findings: We developed and implemented a Kinetic Re-Epithelialization Analysis Pipeline (KREAP) in Galaxy. The KREAP toolbox incorporates a freely available image analysis tools and automatically performs image segmentation and feature extraction of each image series, followed by automatic quantification of cells inside and outside the scratched area over time. The enumeration of infiltrating cells over time is modelled to extract three biologically relevant parameters that describe re-epithelialization kinetics. The output of the tools is organized, displayed, and saved in the Galaxy environment for future consultation. Importantly, a Pearson correlation analysis confirmed that the results obtained using separate software tools and manual determination of the scratch boundaries were comparable to the results obtained with the fully automated KREAP toolbox.

Conclusions: The Galaxy KREAP toolbox provides an open-source, easy-to-use, web-based platform for reproducible image processing and data analysis of high-throughput scratch assays. The KREAP toolbox could assist a broad scientific community in the discovery of compounds that are able to modulate re-epithelialization kinetics.

Keywords: Galaxy, scratch assay, high-throughput, cell migration, re-epithelialization, image analysis, workflow, modelling.

Findings

Background

Cell migration and proliferation play an essential role in a variety of physiological processes, including embryogenesis, angiogenesis, skin and intestinal renewal, and wound repair [1, 2]. Deregulation of these processes can contribute to the development and progression of multiple diseases such as osteoporosis, rheumatoid arthritis, vascular disease and cancer [1]. Therefore, studying the molecular mechanisms underlying the processes of cell migration and proliferation is not only important for obtaining fundamental scientific insight, but it is also essential for the development of effective therapeutic strategies that could modulate these processes when they

have become dysregulated.

The *in vitro* scratch assay is a well-established and widely used method to study cell migration and proliferation [3-5]. The assay is based on the introduction of a scratch into a confluent epithelial cell monolayer to create a "wounded" area. Cells migrate and proliferate into the site of injury in a process known as re-epithelialization [6]. This process is typically monitored by acquisition of images at the beginning and at one or more fixed time points during re-epithelialization. The image series of a particular treatment are then compared to those of the non-treated control to determine the treatment's modulatory capacity in the healing process. The development and constant improvement of image segmentation algorithms over the past decades has enabled the transition from manual quantification of the scratch area to automated analysis that is compatible with high-throughput research [7-9].

CellProfiler [10] and ImageJ [11] are freely available image analysis software tools that allow scientists with limited programming skills to conduct efficient image segmentation and feature extraction of high-throughput image datasets. However, to optimally use their capacities, scripting and parsing of data are often necessary, which require programming skills that many biologists do not have. Commercial software such as FCS Express Image Cytometry (De Novo Software, CA, USA) [12] and Image-Pro Premier (Media Cybernetics, WA, USA), among others, provide alternatives to ease data analysis, but require the purchase of costly licenses. TScratch is an open-source application that was exclusively designed to perform automated scratch assay analyses by measuring the area occupied by cells in image-datasets, but lacks the ability to extract real-time kinetic data [13]. More recently, CellMissy [14] and AVeMap [15] were developed to provide an open-source tool to automate image analysis and provide a determination of the re-epithelialization velocity ($\mu m^2/h$) based on the area occupied by a cell population in image series. Nevertheless, the biological properties of certain cells may render these calculations challenging. For instance, poorly adherent cells (e.g. FHs-74 small intestinal cells) do not migrate collectively, but detach and migrate individually in uneven patterns, making wound area measurements

inaccurate [16]. In addition, this type of measurements hampers the possibility of single-cell analysis, which could include the determination of cell trajectories as well as the enumeration of viable, apoptotic and dead cell ratios [16, 17]. Thus, there is an increasing need for an open-source platform based on single-cell recognition, which could integrate different validated tools for image segmentation, visualization, and data analysis of the processes of cell migration and proliferation involved in wound repair, providing a higher resolution to the current scratch assay analysis.

We developed and implemented a Kinetic Re-Epithelialization Analysis Pipeline (KREAP) in Galaxy (https://galaxyproject.org/) [18] to deliver a web browser-based application for quantitative analysis of *in vitro* scratch assays based on single-cell recognition. The user needs to download and install a virtual machine (VM) containing a fully operational KREAP Galaxy installation. Once the VM is installed, the user uploads the images from a multi-well plate experiment into the VM, together with its corresponding index file, and press the *Execute* button to automatically perform single-cell segmentation and feature extraction in all the images. Enumeration of cells inside and outside the scratch is also carried out automatically over the time series. Based on the number of cells infiltrating the scratch over time, KREAP extracts three biological comprehensive parameters that describe the kinetics of re-epithelialization. In addition, the user's history is saved in the VM for future consultation and the results can be easily shared with other users by downloading the history of multiple experiments. Taken together, we provide a platform that enables reproducible data processing and analysis of high-throughput scratch assays: from raw images to re-epithelialization kinetics.

Implementation

The scratch assay analysis workflow was developed within our own laboratory [19] (also see Methods) and involved a multi-software approach to acquire images, perform image analysis, visualize extracted data, and model re-epithelialization kinetics based on the enumeration of cells migrating into the scratch area over time. CellProfiler (http://cellprofiler.org/) was used in the

original workflow and implemented in the KREAP toolbox (version 2.2.0) to perform automated image segmentation and feature extraction of image series. FCS Express 4 Plus (De Novo Software, CA, USA) was originally used to relate the features extracted by CellProfiler back to the raw images and to enumerate the cells infiltrating the scratched area over time. Since FCS Express 4 Plus requires the purchase of a license, we developed and implemented an R [20] script in the KREAP toolbox that can automatically recognize the scratch boundaries and determine the number of cells inside and outside the scratch over time. Modelling of re-epithelialization kinetics was programmed in R and also implemented into the KREAP toolbox workflow. The workflow is provided in a fully operational Galaxy installation inside of a VM that can be retrieved from the KREAP homepage (https://erasmusmc-bioinformatics.github.io/KREAP/). The VM can be executed in Mac OS X using the freely available VirtualBox (https://www.virtualbox.org/wiki/Downloads). Microsoft Windows and/or Linux users can execute the VM using VirtualBox or VMware Workstation Player (http://www.vmware.com). The source code is available as open-source via the GitHub repository.

Analyses workflow, data acquisition and handling

KREAP was designed to perform single-cell segmentation and therefore, labelling of cell organelles (e.g. nuclei, cytoplasm, cell membranes) with live-cell compatible dyes is recommended to increase the accuracy of the image analysis. We also encourage scientists to verify that the fluorescent dyes and concentrations chosen for their experiments, do not interfere with the normal response of the cells to particular stimuli (see **Additional file 1**). Image acquisition should be carried out with a low magnification objective (e.g. 4x objective; 40x total magnification) to obtain a complete view of the scratched area and surrounding cells [19]. Images (.tif) derived from a multi-well plate must be converted into grayscale, organized in folders by well, and indexed accordingly in a separate file (.txt). An exemplary index and input files are provided at the KREAP homepage (https://erasmusmc-bioinformatics.github.io/KREAP/file_formats). The folders containing the image series of each well are then compressed into a .zip file, which is uploaded into the Galaxy history via the "Get data" tool together with its corresponding index file (**Fig. 1**). The KREAP

toolbox, consisting of the "Image Analysis" and "Data-Modelling" tools, can be executed within the Galaxy platform. At the end of each processing step, the results are provided as HTML and stored in the Galaxy history for future consultation. If desired, the graphs (.png) and tables (.txt) generated by both tools can be downloaded for all wells as a .zip file by clicking the *Save* icon in the Galaxy history.

Image Analysis tool

Once the input files are uploaded into the Galaxy history, the "Image Analysis" tool can be executed (**Fig. 1**). The tool uses an image segmentation pipeline developed in the open-source software CellProfiler 2.2.0 (http://cellprofiler.org/). The individual modules contained in the pipeline carry out automated extraction of cellular features in every image. An illumination function is calculated in the first segmentation module by finding the minimum pixel intensities in blocks (e.g. block size 10-20 pixels) across each image and applying a Gaussian filter [21] as smoothing method. In the second module, the calculated illumination function is applied to the raw image by subtraction, resulting in better contrast between the cells and the background. Identification of primary objects is defined in the third module as objects within a specified diameter range (in pixels), depending on the cell type used. Identification of primary objects is performed by applying a global threshold strategy in combination with the Otsu algorithm [22] which calculates a single threshold value that classifies pixels above the threshold as foreground and below the threshold as background. Because objects tend to be brighter towards the interior than towards the edges, the difference in intensity is used to separate merged objects into individual ones. The last module extracts phenotypic features (e.g. size, eccentricity and mean intensity) from each object as well as their x- and y-coordinates within the image. For optimal image segmentation results, the user can adjust the parameters for illumination correction (i.e. block size) and object identification (i.e. minimum and maximum object diameter size) for each well directly in the index file. However, it is important to remark that for an objective comparison, it is recommendable to use the same parameters across wells seeded with the same cell type.

The graphical interface of Galaxy provides the user with an overview of each well within the multi-well plate (**Fig. 2**). The location of the identified primary objects is visualized in an interactive plot that uses a slider to move through images over time. A compare function is provided to visually evaluate the performance of the image segmentation pipeline by comparing its output with the raw image. Automatic identification of the scratch boundaries was optimised using a customized R script. The script finds the largest empty area by measuring the frequency of cells on the Y axis at the beginning of the assay. The total number of cells is determined in each image over time and classified into objects inside or outside of the scratched area (**Fig. 2**). The image segmentation results are stored in the Galaxy history and can be accessed by the user in the future. Furthermore, the cellular features extracted by CellProfiler and the enumeration of cells inside or outside of the scratched area can be easily downloaded via the links provided in the output.

Data-Modelling tool

The output derived from the image analysis can be used in the "Data-Modelling" tool to extract three biologically relevant parameters that describe the kinetics of re-epithelialization (**Fig. 1**). To calculate the parameter values, the time interval between images must be entered in the index file (see https://erasmusmc-bioinformatics.github.io/KREAP/file_formats). The enumeration of cells infiltrating the scratched area over time consistently results in a sigmoidal curve (**Fig. 3**) [19], similar to the ones obtained with bacterial growth curves that are characterized by having a lag phase, an exponential phase, and a stationary phase. The modified Gompertz function has been successfully used to model bacterial growth to estimate three biologically relevant parameters (λ, μ_m, and A) that mathematically describe the different phases of growth [23]. We developed and implemented an R script that uses a nonlinear least squares regression to fit the modified Gompertz function through the re-epithelialization measurements [19]. Furthermore, we applied the Levenberg-Marquardt algorithm [24] to reduce the sum of the squares of the errors between the modelled and measured data points in an iterative manner. In this way, we were able to obtain excellent fits that were characterised by R^2 values close to 1 and low root-mean-square error (RMSE) values. The

modified Gompertz function describes the re-epithelialization kinetics for each image series through the estimation of the lag time (λ; in minutes), the repair rate (μ_m; cells/minute), and the maximum number of cells within the scratched area at the plateau of the re-epithelialization curve (A; in cells). The λ parameter represents the time in minutes before cell migration into the scratched area starts. For some cell lines (e.g. Ca9-22), the lag time can be very brief and the migration process may start even before image acquisition takes place according to the model outcome [19]. In those cases, the λ parameter would be estimated to be zero or have negative values and thus, the biological contribution of this parameter to the description of re-epithelialization kinetics would be limited, but its calculation remains essential for obtaining a good fit of the model. Apart from the λ parameter, the values obtained for the repair rate (μ_m parameter) and the maximum number of infiltrating cells (A parameter) for each replicate condition can be used to compare the average response of cells treated with a specific substance to that of the non-treated control. Importantly, we have shown that the A parameter is correlated to the maximum surface area in pixels covered by cells (r = 0.68, P = 1.36 x 10-6) [19]. The overall effect of a particular substance on re-epithelialization kinetics can be assessed by calculating the performance value (μ_m*A), which incorporates the μ_m and the A parameters into a single value. For this calculation, the μ_m and the A values of each replicate are normalized against the corresponding average value of the non-treated control [19]. This performance value can be used in screenings as a simple way to identify lead substances that could potentially stimulate or attenuate wound repair in comparison with the non-treated control.

Anticipated results

As an exemplary dataset, gingival epithelial cells (Ca9-22) were seeded in 96-well plates and incubated overnight to obtain a confluent cell monolayer. During the 2 hours of starvation (i.e. incubation with FCS-free DMEM), nuclei were stained with 2 μg/ml Hoechst 33342 for 20 minutes. This is particularly important when testing the influence of certain substances that could aggregate (e.g. bacteria) and therefore, interfere with accurate recognition of single cells when using bright-

field microscopy (**Fig. 4a**). After the 2 hours of starvation were completed, the cell monolayers were scratched to create an artificial wound in each well. The wells were then washed twice with phosphate buffered saline (PBS) to remove the nuclear staining-solution and detached cells. Human transforming growth factor α (hTGFα) acted as a mitogenic and mobility factor (**Fig. 4b)** through the activation of the epidermal growth factor receptor (EGFR) [25]. In contrast, addition of chemical inhibitors of p38 and MEK1/2 phosphorylation led to suppression of cell migration and ERK1/2-mediated proliferation (**Fig. 4b)**, respectively [26, 27]. Calculation of the kinetic parameters describing re-epithelialization kinetics showed that TGFα-stimulated cells had a significantly higher repair rate (*P* = 0.015) when compared with the untreated cells (**Fig. 4c**). Likewise, stimulation with TGFα resulted in significantly higher numbers of cells inside the scratched area in comparison with the non-treated control (*P* = 0.004) (**Fig. 4d**). Inversely, treatment with the solution containing p38 and MEK1/2 inhibitors resulted in a lower repair rate and significantly reduced numbers of infiltrating cells (*P* = 0.0008) when compared to the non-treated control during the 5 hours scratch assay (**Fig. 4c** and **4d**).

Validation of the KREAP tool with published sub-dataset

The performance of KREAP was evaluated in comparison with the workflow used in a previous study in which we employed subsequent steps that involved the use of multiple software tools and the manual determination of the scratch boundaries [19]. We re-analysed a sub-dataset from that study (215 image series) using the KREAP toolbox in the Galaxy platform. Data processing was performed on a desktop with an Intel® Core™ i7-3970X processor with 4 cores at 3.50 GHz and 4 GB of RAM using Windows 7. Both KREAP and the original workflow use CellProfiler for automated image segmentation and feature extraction. However, in the original workflow, data visualization and enumeration of infiltrating cells over time was determined using the commercially available FCS Express 4 Plus (De Novo Software, CA, USA) software tool. The location of the identified objects at the beginning of the assay was plotted in a scatterplot after which a rectangular gate was manually placed on the scratched area and a batch process was setup to record the number of

infiltrating cells over time for each well [19]. FCS Express 4 Plus was replaced in the KREAP Image Analysis tool by a customized R script that automatically recognizes the boundaries of the scratch. To assess the performance of the KREAP Image Analysis tool in comparison with the manual determination of the scratched area used in our original study [19], the kinetic parameter values (i.e. μ_m and A parameters) obtained for the sub-dataset were compared by a Pearson correlation analysis. Importantly, the analysis confirmed the association between the values obtained in the previous study with those obtained with the KREAP toolbox for both the μ_m and A parameters with correlation values of 0.87 ($P < 0.0001$) and 0.83 ($P < 0.0001$), respectively (**Fig. 5**). These results eliminate the need for manual data-handling by the user and significantly reduces the time required for performing the analysis. For example, processing of a multi-well plate experiment consisting of 60 wells and 16 timepoints (960 images in total) with the original workflow would typically take around 3 to 4 hours for an experienced user to complete. In contrast, the KREAP toolbox can perform the complete analysis –from raw images to quantification of re-epithelialization kinetics– in less than 30 minutes without the need for programming skills.

Identification of detrimental effects on re-epithelialization and troubleshooting

The modified Gompertz function is used to model growth (i.e., positive sigmoidal curves), but identification of detrimental effects on re-epithelialization kinetics is still possible through inspection of the curves generated with the measured and modelled data points. In the first example (**Fig. 6a**), cells had migrated into the scratched area until a plateau was reached, but this was followed by a reduction in the number of infiltrating cells over time as a result of cell death, leading to a low R^2 value. The KREAP Data-Modelling tool flags R^2 values lower than 0.9 to be inspected by the user. In the second example (**Fig. 6b**), cell migration was strongly inhibited by the treatment and consequently, only very few cells migrated into the scratched area over the course of the experiment. Because the resulting curve did not have a sigmodial shape, the modified Gompertz function was unable to model the curve and thus, the parameter values describing re-epithelialization kinetics were not be retrieved. The KREAP Data-Modelling tool flags the wells in

which the parameter values are not be retrieved. The user can then decide to exclude the treatment from the analysis by clicking the icon in the *Include* column and have a new index file automatically created through the provided link (**Fig. 6c**). In experiments with enough technical replicates, this function can also be used to remove outliers from the final analysis. Finally, if the assay is too short for a particular treatment to reach the plateau phase of the growth curve (**Fig. 6d**), the estimation of the parameter values could be overestimated, resulting in outliers. In this case, the user is advised to extend the timeframe of the assay until the plateau phase is reached.

Conclusions

A key aspect of high-throughput microscopy research is to convert the raw images into quantitative and biologically comprehensive data. This step often requires multiple software tools, programming skills or purchase of costly software to aid in the image processing and data analysis. The KREAP toolbox integrates multiple validated tools for image segmentation, visualization and data analysis of high-throughput scratch assays in Galaxy. Furthermore, the implementation of the KREAP toolbox in Galaxy provides an open-source web-based platform that enables scientists that lack sophisticated programming skills to perform the complete analysis starting with the raw images and ending with the quantified kinetics based on single-cell recognition. This approach provides an accurate enumeration of cells inside and outside of the scratched area during the course of the experiment as well as the opportunity to performed refined experiments, which intend to track single cells, determine cell proliferation and/or viable/dead cell rations. In addition, the graphical user interface of Galaxy provides an easy-to-use environment that organizes, displays, and saves the results of every experiment as part of the user history. The KREAP toolbox in Galaxy provides an “end to end” integrated analytical high-throughput screening platform that is useful for scientists who are interested in the discovery and mechanistic analysis of compounds that can modulate re-epithelialization kinetics.

Methods

Cell line

Gingival epithelial cells (Ca9-22) were purchased from the National Institute of Biomedical Innovation JCRB Cell Bank (Osaka, JCRB0625). Ca9-22 cells were cultured in Dulbecco's Modified Eagle Medium (DMEM) containing Glutamax (Gibco, Invitrogen, Paisley, UK), 10% fetal calf serum (FCS), 100 U/ml penicillin and 100 μg/ml streptomycin (Sigma-Aldrich, MO, USA). Cells were cultured at 37°C in a humidified atmosphere containing 5% CO_2 and passaged when a 70% confluency was reached.

Scratch assay and image acquisition

The experiment was carried out as described in [19]. Briefly, Ca9-22 cells were seeded in 96-well plated (BD Falcon™, Corning, NY, USA) at a density of 3.5×10^4 cells/well and incubated over night to obtain a confluent cell monolayer. The next day, cells were starved in FCS-free DMEM for 2 hours to decrease the basal cell proliferation. During the last 20 minutes of starvation, nuclei were stained with FCS-free DMEM containing 2 μg/ml Hoechst 33342. Following the starvation, equally sized scratches (0.3 x 2 mm) were introduced in the cell monolayers with the HTSScratcher (Peira, Antwerpen, BE). After washing the cells twice with phosphate buffered saline (PBS), the treatments (for details see [19]) were added into the wells in a randomized manner using three technical replicates. The positive control consisted of 4 ng/ml human transforming growth factor (TGFα; R&D Systems, MN, USA); a combination of inhibitors of p38 (SB203580; Cell Signaling Technology, MA, USA) and MEK1/2 (U0126, Cell Signaling Technology) at a concentration of 10 μM each, served as negative control. FCS-free DMEM was used as non-treated control. The overall quality of each run of the 96-well based assay was assessed by calculation of the Z' factor, which establishes a dynamic range between the positive and negative control values [28]. Images were acquired using the BD Pathway 855 Bioimaging System (BD Biosciences, CA, USA) under controlled temperature and atmosphere (37°C and 5% CO_2). Fluorescent images were acquired using an

excitation filter of 350 nm. The BD Pathway platform was programmed to acquire the same field of each well every 20 minutes for 5 hours using a 4x objective.

Availability and Requirements

- Project name: KREAP (Kinetic Re-Epithelialization Analysis Pipeline)
- Project home page: https://erasmusmc-bioinformatics.github.io/KREAP/
- Operating system: KREAP was developed in Linux and can be executed in Unix-based operating systems, Microsoft Windows or Mac OS X.
- Programming languages: Python, R programming language
- License: Freely available under the MIT open source license
- Any restriction to use as non-academic: none
- Virtual machine accessibility: via the KREAP homepage and GitHub repository

Availability of supporting data

The dataset supporting the results of this article is available at the KREAP homepage (Kinetic Re-Epithelialization Analysis Pipeline).

Competing interests

The authors declare there that they have no competing interests.

Authors' contributions

MMFG, DHBvZ, PvB, MK and AS conceived the study and contributed to writing the first draft of the manuscript. MMFG performed the scratch assays and carried out the validation analyses. DHBvZ and AS developed and implemented the analyses workflow. All authors contributed to editing the final manuscript. All authors read and approved the final manuscript.

References

1. Ridley AJ, Schwartz MA, Burridge K, Firtel RA, Ginsberg MH, Borisy G, et al. Cell Migration: Integrating Signals from Front to Back. Science. 2003;302(5651):1704-9. doi: 10.1126/science.1092053.
2. Friedl P, Gilmour D. Collective cell migration in morphogenesis, regeneration and cancer. Nat Rev Mol Cell Biol. 2009;10(7):445-57.

3. Liang CC, Park AY, Guan JL. In vitro scratch assay: a convenient and inexpensive method for analysis of cell migration in vitro. Nature protocols. 2007;2(2):329-33. doi: 10.1038/nprot.2007.30. PubMed PMID: 17406593.
4. Oudhoff MJ, Van Den Keijbus PAM, Kroeze KL, Nazmi K, Gibbs S, Bolscher JGM, et al. Histatins enhance wound closure with oral and non-oral cells. J Dent Res. 2009;88(9):846-50. doi: 10.1177/0022034509342951.
5. Mohammedsaeed W, Cruickshank S, McBain AJ, O'Neill CA. Lactobacillus rhamnosus GG Lysate Increases Re-Epithelialization of Keratinocyte Scratch Assays by Promoting Migration. Scientific Reports. 2015;5:16147. doi: 10.1038/srep16147.http://www.nature.com/articles/srep16147#supplementary-information.
6. Schäfer M, Werner S. Transcriptional Control of Wound Repair. Annual Review of Cell and Developmental Biology. 2007;23(1):69-92. doi: 10.1146/annurev.cellbio.23.090506.123609.
7. Yarrow JC, Perlman ZE, Westwood NJ, Mitchison TJ. A high-throughput cell migration assay using scratch wound healing, a comparison of image-based readout methods. BMC biotechnology. 2004;4:21. doi: 10.1186/1472-6750-4-21. PubMed PMID: 15357872; PubMed Central PMCID: PMC521074.
8. Zordan MD, Mill CP, Riese DJ, 2nd, Leary JF. A high throughput, interactive imaging, bright-field wound healing assay. Cytometry Part A : the journal of the International Society for Analytical Cytology. 2011;79(3):227-32. Epub 2011/11/03. doi: 10.1002/cyto.a.21029. PubMed PMID: 22045642; PubMed Central PMCID: PMC3306835.
9. Simpson KJ, Selfors LM, Bui J, Reynolds A, Leake D, Khvorova A, et al. Identification of genes that regulate epithelial cell migration using an siRNA screening approach. Nat Cell Biol. 2008;10(9):1027-38. doi: http://www.nature.com/ncb/journal/v10/n9/suppinfo/ncb1762_S1.html.
10. Lamprecht MR, Sabatini DM, Carpenter AE. CellProfiler™: Free, versatile software for automated biological image analysis. BioTechniques. 2007;42(1):71-5. doi: 10.2144/000112257.
11. Schindelin J, Rueden CT, Hiner MC, Eliceiri KW. The ImageJ ecosystem: An open platform for biomedical image analysis. Molecular Reproduction and Development. 2015;82(7-8):518-29. doi: 10.1002/mrd.22489.
12. Software DN. A Flow Cytometry Analysis Environment for Image Cytometry Data. Available from: https://www.denovosoftware.com/site/Image-Overview.shtml. 6 ed.
13. Geback T, Schulz MM, Koumoutsakos P, Detmar M. TScratch: a novel and simple software tool for automated analysis of monolayer wound healing assays. BioTechniques. 2009;46(4):265-74. Epub 2009/05/20. doi: 10.2144/000113083. PubMed PMID: 19450233.
14. Masuzzo P, Hulstaert N, Huyck L, Ampe C, Van Troys M, Martens L. CellMissy: a tool for management, storage and analysis of cell migration data produced in wound healing-like assays. Bioinformatics. 2013;29(20):2661-3. doi: 10.1093/bioinformatics/btt437. PubMed PMID: PMC3789541.
15. Deforet M, Parrini MC, Petitjean L, Biondini M, Buguin A, Camonis J, et al. Automated velocity mapping of migrating cell populations (AVeMap). Nature Methods. 2012;9:1081. doi: 10.1038/nmeth.2209. https://www.nature.com/articles/nmeth.2209#supplementary-information.
16. Nyegaard S, Christensen B, Rasmussen JT. An optimized method for accurate quantification of cell migration using human small intestine cells. Metabolic Engineering Communications. 2016;3:76-83. doi: https://doi.org/10.1016/j.meteno.2016.03.002.
17. Huang TC, Lee JF, Chen JY. Pardaxin, an antimicrobial peptide, triggers caspase-dependent and ROS-mediated apoptosis in HT-1080 cells. Marine drugs. 2011;9(10):1995-2009. Epub 2011/11/11. doi: 10.3390/md9101995. PubMed PMID: 22073006; PubMed Central PMCID: PMCPMC3210615.
18. Goecks J, Nekrutenko A, Taylor J. Galaxy: a comprehensive approach for supporting accessible, reproducible, and transparent computational research in the life sciences. Genome Biology. 2010;11(8):R86-R. doi: 10.1186/gb-2010-11-8-r86. PubMed PMID: PMC2945788.
19. Fernandez-Gutierrez MM, Roosjen PPJ, Ultee E, Agelink M, Vervoort JJM, Keijser B, et al. Streptococcus salivarius MS-oral-D6 promotes gingival re-epithelialization in vitro through a secreted serine protease. Scientific Reports. 2017;7(1):11100. doi: 10.1038/s41598-017-11446-z.
20. R Development Core Team. R: A Language and Environment for Statistical Computing. Vienna, Austria: R Foundation for Statistical Computing; 2011.
21. Lindblad J, Bengtsson E, editors. A comparison of methods for estimation of intensity nonuniformities in 2D and 3D microscope images of fluorescence stained cells. Proceedings of the 12th Scandinavian Conference on Image Analysis (SCIA); 2001.
22. Otsu N. A Threshold Selection Method from Gray-Level Histograms. IEEE Transactions on Systems, Man, and Cybernetics. 1979;9(1):62-6. doi: 10.1109/TSMC.1979.4310076.
23. Zwietering MH, Jongenburger I, Rombouts FM, van 't Riet K. Modeling of the Bacterial Growth Curve. Applied and Environmental Microbiology. 1990;56(6):1875-81.
24. Elzhov TV, Mullen KM, Spiess A-N, Bolker B. minpack.lm: R Interface to the Levenberg-Marquardt Nonlinear Least-squares Algorithm Found in MINPACK, Plus Support for Bounds. R package version 1.2-1. https://CRAN.R-project.org/package=minpack.lm. 2016.
25. Ebner R, Derynck R. Epidermal growth factor and transforming growth factor-alpha: differential intracellular routing and processing of ligand-receptor complexes. Cell Regulation. 1991;2(8):599-612. PubMed PMID: PMC361851.
26. Huang C, Jacobson K, Schaller MD. MAP kinases and cell migration. Journal of Cell Science. 2004;117(20):4619-28. doi: 10.1242/jcs.01481.

27. Mebratu Y, Tesfaigzi Y. How ERK1/2 activation controls cell proliferation and cell death: Is subcellular localization the answer? Cell Cycle. 2009;8(8):1168-75. doi: 10.4161/cc.8.8.8147.
28. Zhang J-H, Chung TDY, Oldenburg KR. A Simple Statistical Parameter for Use in Evaluation and Validation of High Throughput Screening Assays. Journal of Biomolecular Screening. 1999;4(2):67-73. doi: 10.1177/108705719900400206.

Figure legends

Figure 1. KREAP workflow. The virtual machine contains the KREAP toolbox and uses the graphical user interface (GUI) provided by Galaxy, including HTML reporting. The KREAP toolbox consists of the Image Analysis and Data-Modelling tools. Logos indicate the use of specialized (open source) software or programming environments in different stages of the data processing. Red parallelograms indicate input and green parallelograms indicate output. Python was used to integrate the non-Galaxy applications into Galaxy tools.

Figure 2. KREAP "Image Analysis" graphical output example. Image segmentation output can be easily compared with the raw image. Automatic recognition of scratch boundaries enables the enumeration of nuclei inside and outside of the scratched area over time.

Figure 3. KREAP Data-Modelling tool output example. Re-epithelialization kinetics described by the λ, μ_m and A parameters. The parameter values, simulation data and re-epithelialization curves per replicate are provided in an HTML report and are also available for download through the available links.

Figure 4. Exemplary results obtained with the KREAP toolbox. (a) Ca9-22 cells were exposed to a bacterial preparation (MOI 250) to test for its modulatory effect on re-epithelialization. Nuclei were labelled with 2 μg/ml Hoechst 33342 after which bright-field and fluorescent images were acquired. Single-cell recognition was carried out on the fluorescent images, which provides an accurate enumeration of the cells (c) Comparison of the repair rate (μ_m parameter) obtained with cells treated with TGFα (4 ng/ml), a solution containing p38 and MEK1/2 inhibitors (10 μM each) and left untreated. (d) Comparison of the maximum numbers of infiltrating cells (A parameter) obtained with the different treatments). Significant differences from the non-treated control were assessed by a one-way ANOVA using a Dunnett's test for multiple comparisons (n = 3; *, $P < 0.05$; **, $P < 0.01$; ***, $P < 0.001$).

Figure 5. Association between the parameter values originated with a multi-software approach and the KREAP toolbox. (a) Repair rate (μ_m parameter, cells minute^{-1}) and (b) Maximum number of cells (A parameter, cells). The association of the parameter values was evaluated by Pearson correlation analysis (n = 215) and for both cases a positive and significant correlation was found ($P < 0.0001$).

Figure 6. Identification of detrimental effects and outliers using KREAP. (a) Detrimental effect on re-epithelialization characterized by a low R^2 value as a result of extensive cell death after reaching the plateau of the growth curve. (b) Strong inhibitory effect results in migration of very few cells into the scratched area and as a result the parameter values that describe re-epithelialization cannot be retrieved. (c) The user can decide to exclude certain wells of the analysis by pressing the icon in the *Include* column. (d) Overestimation of the parameter values can result from growth curves that do not reached the plateau phase in the timeframe of the assay.

Click here to download Figure Figure 1.pdf

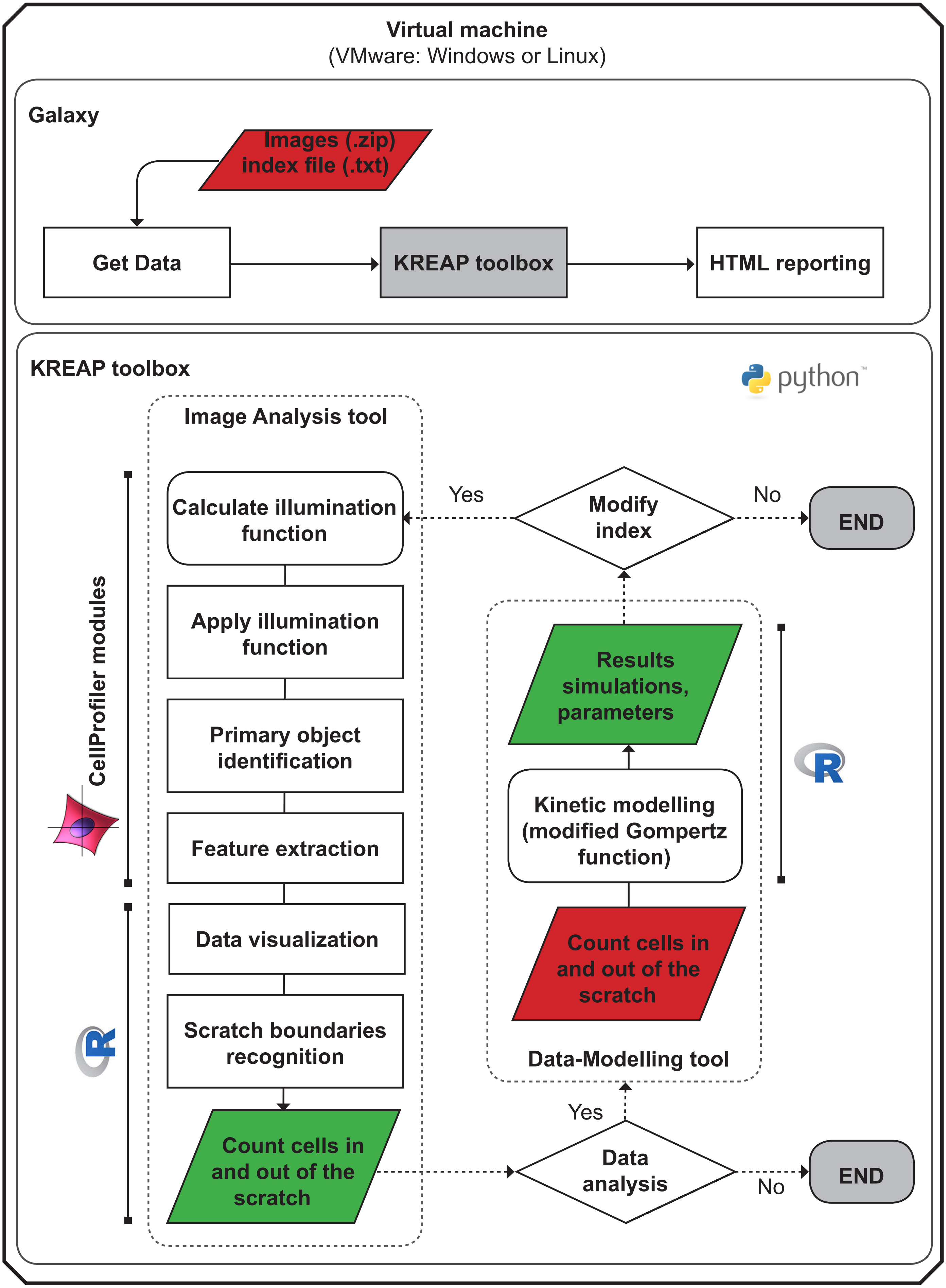

Figure

Click here to download Figure Figure 2.pdf

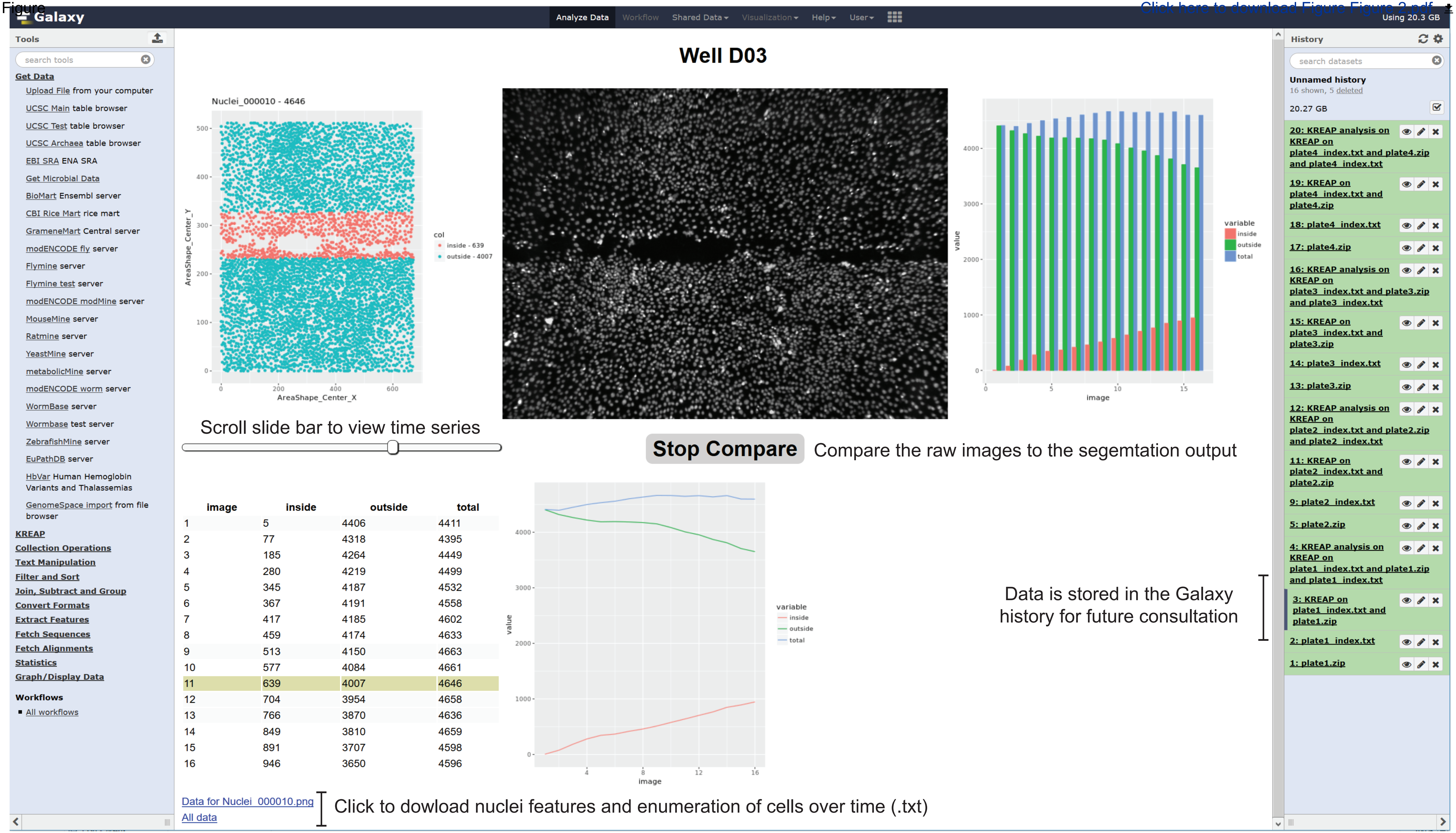

Figure

Click here to download Figure Figure 3.pdf

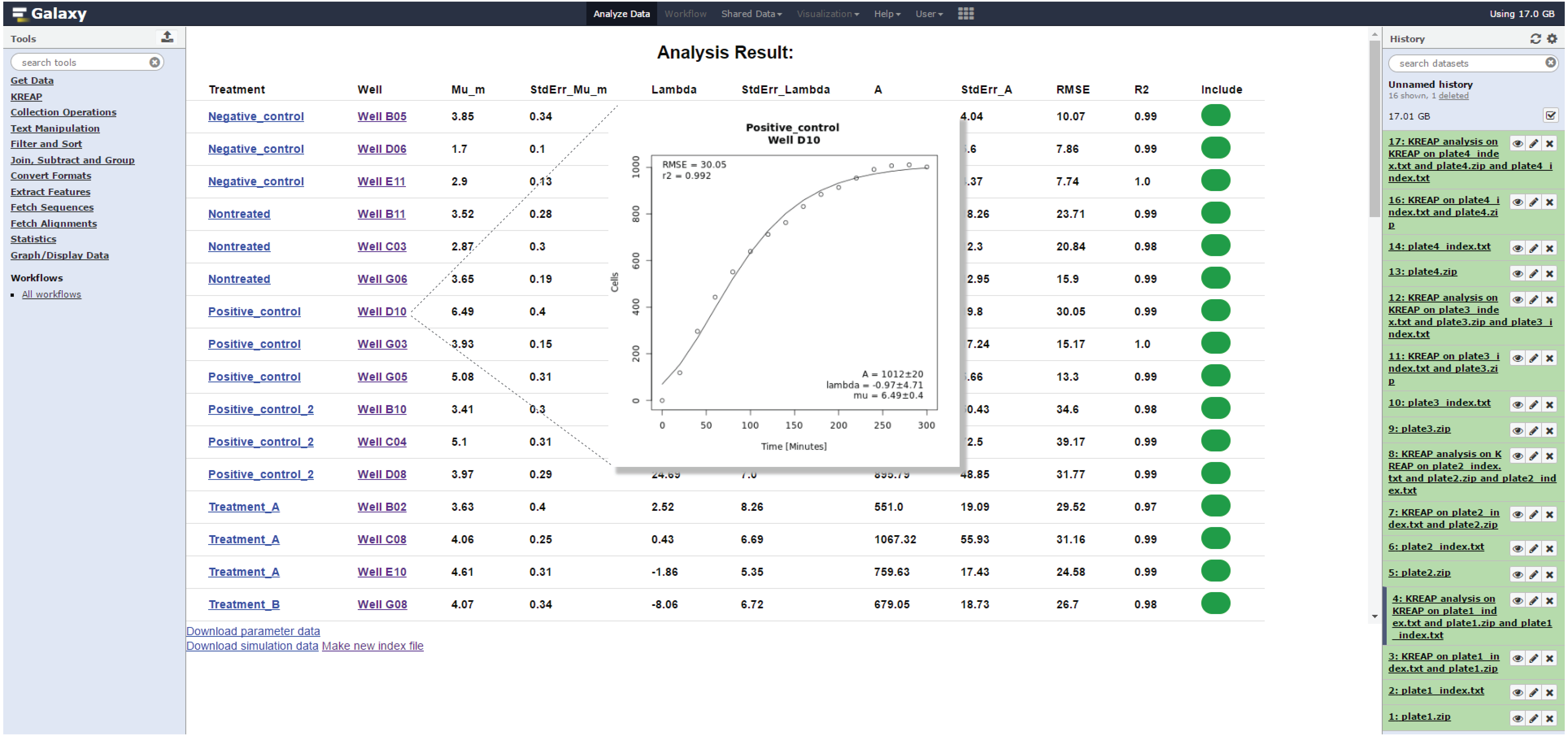

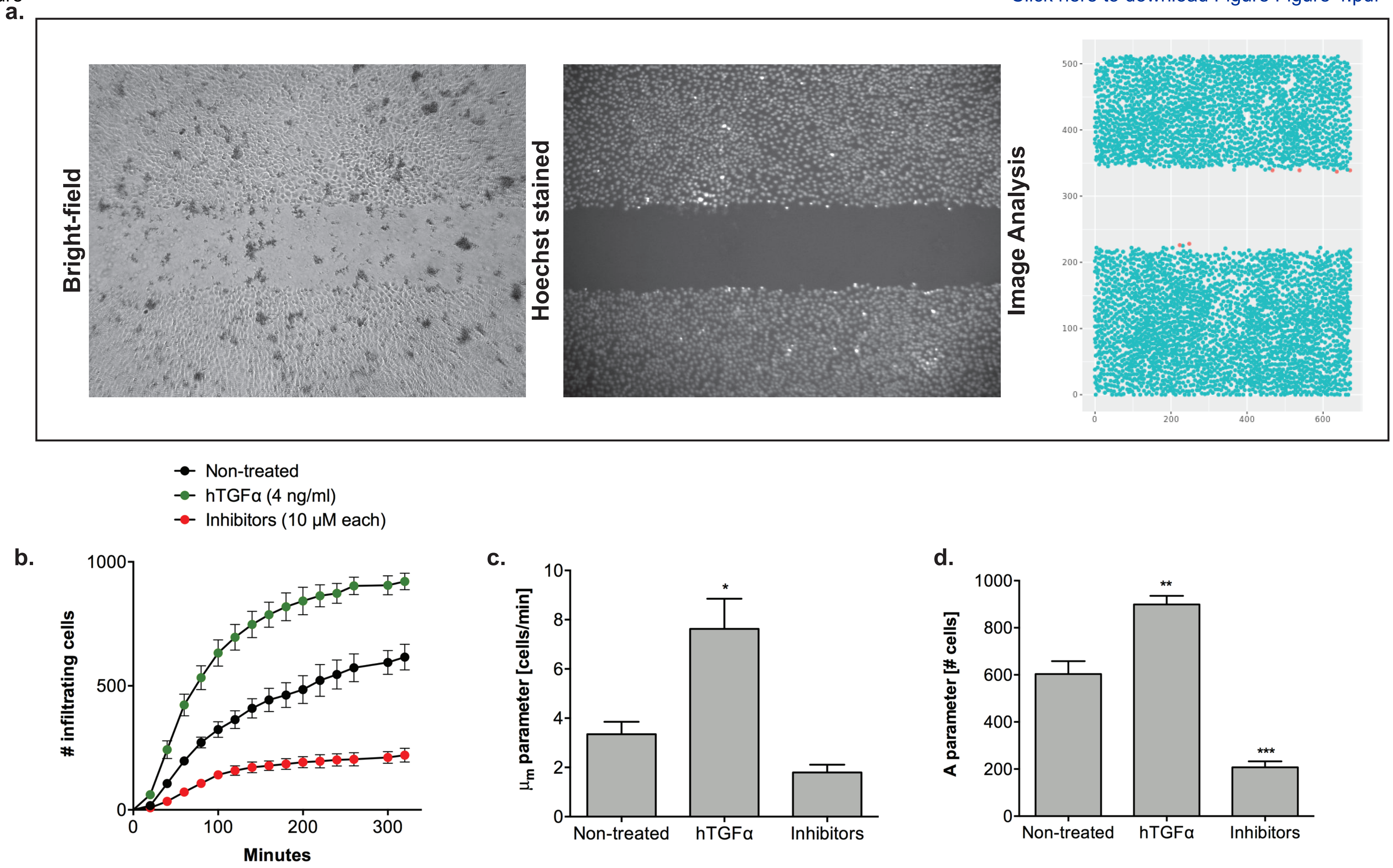
a.
Bright-field
Hoechst stained
Image Analysis
Non-treated
hTGFα (4 ng/ml)
Inhibitors (10 μM each)
b.
infiltrating cells
Minutes
c.
μm parameter [cells/min]
Non-treated
hTGFα
Inhibitors
d.
A parameter [# cells]
Non-treated
hTGFα
Inhibitors

Click here to download Figure Figure 5.pdf

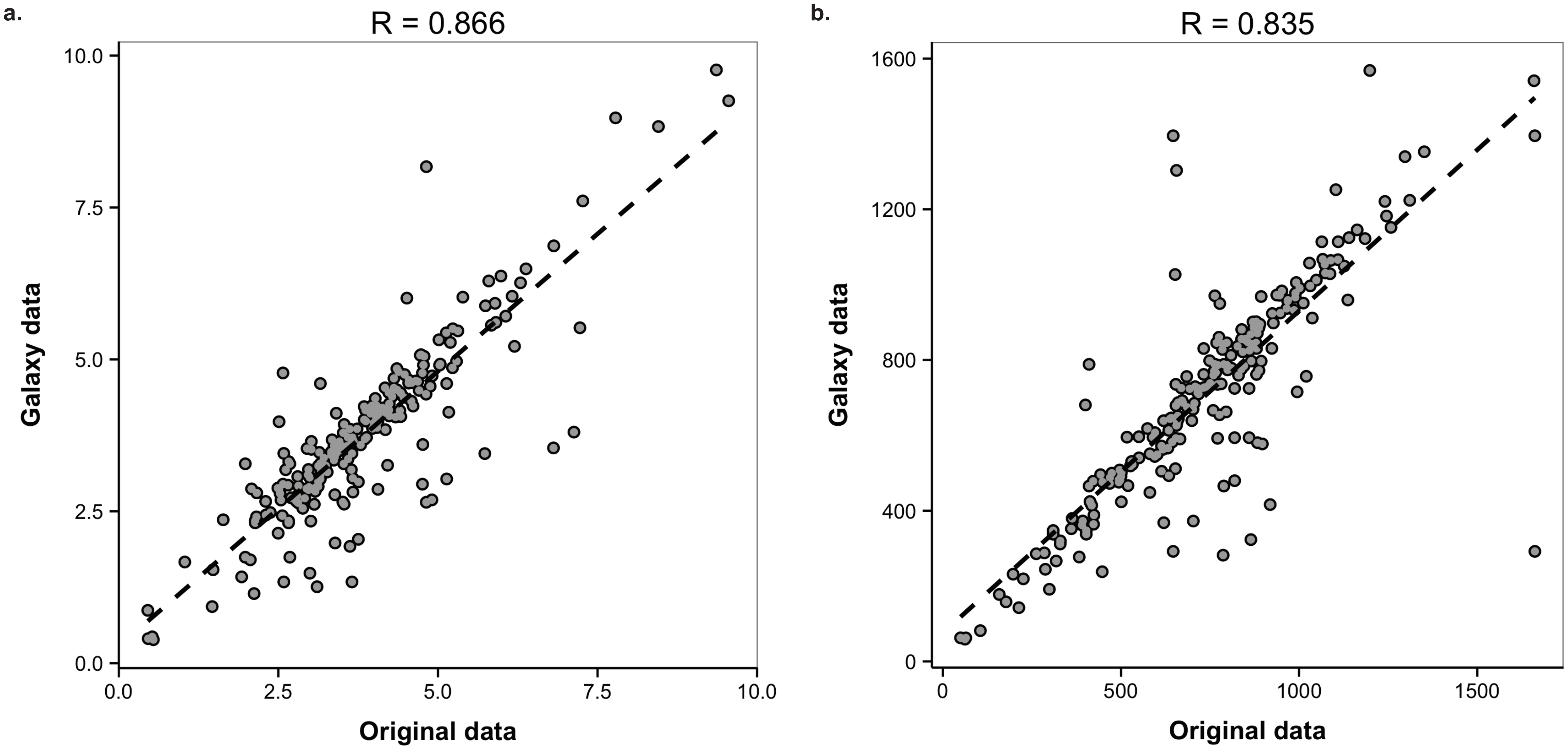

Figure

Click here to download Figure Figure 6.pdf

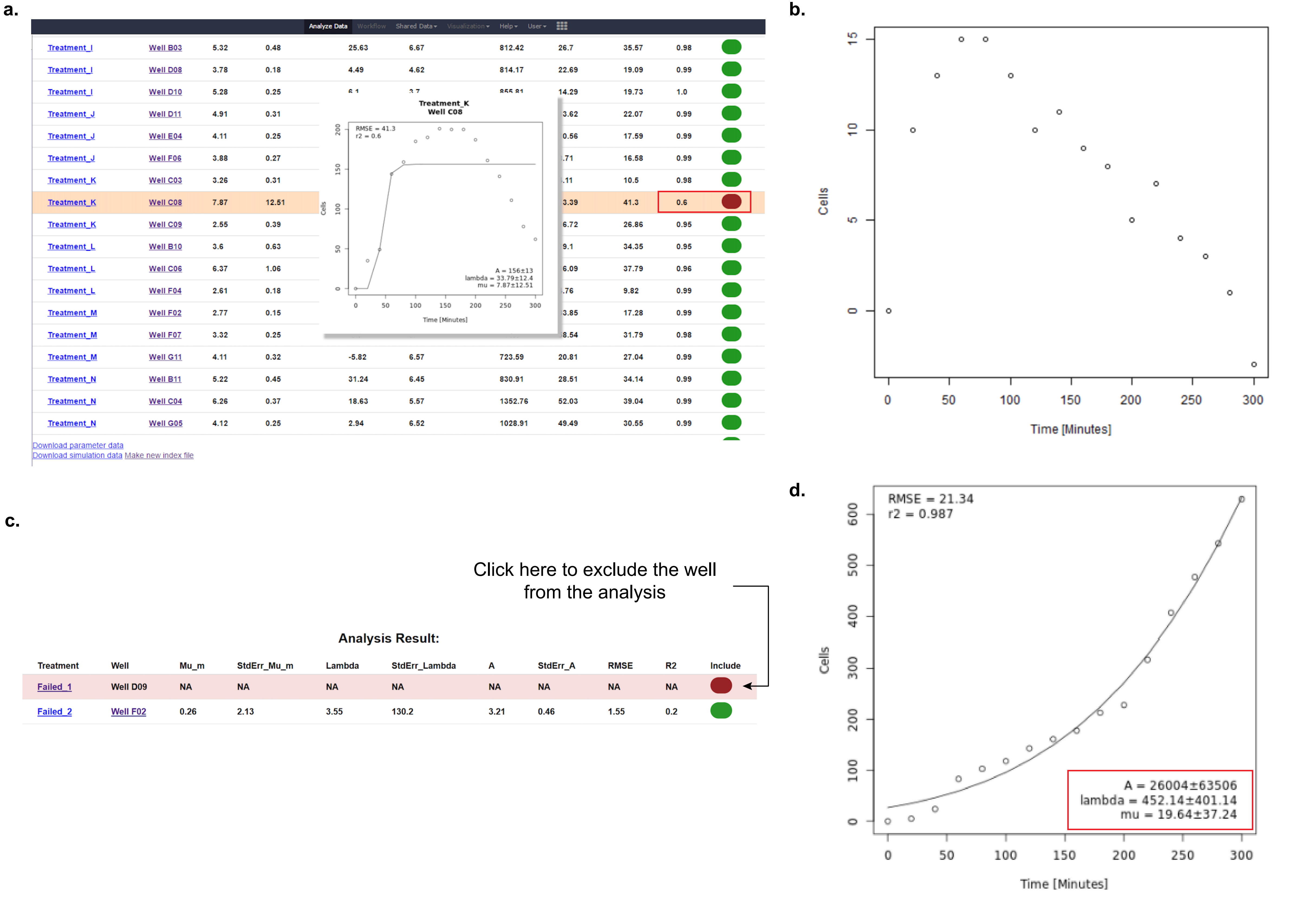

Additional File 1

Click here to access/download
Supplementary Material
Additional File 1.pdf

Additional Manuscript

Click here to access/download
Supplementary Material
Additional Manuscript.pdf

Click here to download Personal Cover
Cover_letter_GiagaScience.pdf

Erasmus MC
Universitair Medisch Centrum Rotterdam

Clinical Bioinformatics
Department of Pathology
Andrew Stubbs, PhD

Scott Edmunds
Editor
Gigascience,

Direct dial +31-10-704.4776
Room number Ee 15-76
E-mail a.stubbs@erasmusmc.nl
Date February 27th, 2018

Dear Scott,

We wish to re-submit our manuscript entitled "**KREAP: An automated Galaxy Platform to Quantify *in vitro* Re-epithelialization Kinetics**" by Fernandez-Gutierrez *et al.* as technical note manuscript to Gigascience.

Our aim was to develop an open source application to support the analysis of high-throughput microscopy and image analysis data from *in vitro* scratch assays commonly used to study the influence of bioactive substances on the processes of cell migration and proliferation. To address these challenges we developed and implemented a **K**inetic **R**e-**E**pithelialization **A**nalysis **P**ipeline (KREAP) in Galaxy which provides an "end to end" solution image processing, quantitation and visual reporting including parameters that describe re-epithelialization kinetics.

We have altered our manuscript in accordance with the reviewer's comments and an associated document to address these comments point by point. We hope that both the revised manuscript and the associated discussion of reviewer's comments are sufficient to ensure that our article accepted for publication.

We appreciate the time that you and the reviewers have taken critically evaluate our research and to improve our representation of KEAP to the scientific community

We also provide anonymous login details to access the KREAP code at https://github.com/ErasmusMC-Bioinformatics/KREAP (username: erasmusmc-review; password: erasmusmcreview123).

Yours sincerely,

Andrew Stubbs, PhD

Assistant Professor of Bioinformatics
