# Supplementary material for: KREAP: an automated Galaxy platform to quantify in vitro re-epithelialization kinetics
Source: Gigascience. 2018 Jun 28;7(7):giy078. doi: 10.1093/gigascience/giy078 (PMC6048990; doi:10.1093/gigascience/giy078)
Supplement: GIGA-D-17-00209_Revision_2.pdf [file giy078_giga-d-17-00209_revision_2.pdf]

GigaScience

KREAP: An automated Galaxy Platform to Quantify in vitro Re-Epithelialization Kinetics

--Manuscript Draft--

**Manuscript Number:**	GIGA-D-17-00209R2
**Full Title:**	KREAP: An automated Galaxy Platform to Quantify in vitro Re-Epithelialization Kinetics
**Article Type:**	Technical Note
**Funding Information:**	
**Abstract:**	Background: In vitro scratch assays have been widely used to study the influence of bioactive substances on the processes of cell migration and proliferation that are involved in re-epithelialization. The development of high-throughput microscopy and image analysis has enabled scratch assays to become compatible with high-throughput research. However, effective processing and in-depth analysis of such high-throughput image-datasets is far from trivial and requires integration of multiple image processing and data extraction software tools.<br><br>Findings: We developed and implemented a Kinetic Re-Epithelialization Analysis Pipeline (KREAP) in Galaxy. The KREAP toolbox incorporates freely available image analysis tools and automatically performs image segmentation and feature extraction of each image series, followed by automatic quantification of cells inside and outside the scratched area over time. The enumeration of infiltrating cells over time is modelled to extract three biologically relevant parameters that describe re-epithelialization kinetics. The output of the tools is organized, displayed, and saved in the Galaxy environment for future consultation.<br><br>Conclusions: The Galaxy KREAP toolbox provides an open-source, easy-to-use, web-based platform for reproducible image processing and data analysis of high-throughput scratch assays. The KREAP toolbox could assist a broad scientific community in the discovery of compounds that are able to modulate re-epithelialization kinetics.<br><br>Keywords: Galaxy, scratch assay, high-throughput, cell migration, re-epithelialization, image analysis, workflow, modelling.
**Corresponding Author:**	Andrew Stubbs<br><br>NETHERLANDS
**Corresponding Author Secondary Information:**	
**Corresponding Author's Institution:**	
**Corresponding Author's Secondary Institution:**	
**First Author:**	Marcela M. Fernandez-Gutierrez
**First Author Secondary Information:**	
**Order of Authors:**	Marcela M. Fernandez-Gutierrez
	David B.H. van Zessen
	Peter van Baarlen
	Kleerebezem Michiel
	Andrew P. Stubbs
**Order of Authors Secondary Information:**	
**Response to Reviewers:**	Dear Scott Edmunds,<br><br>Below we present our point-to-point response to the referee on the technical note GIGA-D-17-00209 entitled "KREAP: An automated Galaxy Platform to Quantify in vitro

Powered by Editorial Manager® and ProduXion Manager® from Aries Systems Corporation

Re-Epithelialization Kinetics". Once again, we thank the reviewers for their time and critical input.

Major issues:
1.Limitation to live imaging of nuclei stained with fluorescent markers.
1.1.I advise the authors to focus on this point (that the major benefit of counting single nuclei is for the case of cells with weaker cell-cell adhesion that detach from the bulk and migrate individually) and down-play the arguments on bacteria or viability, which are quite esoteric and not relevant for the vast majority of potential users.

Author response: As recommended by the reviewer, the argument on bacteria or cell viability is now removed from the revised manuscript. However, we believe that there is increased interest on exploring the influence of bacteria on wound repair and therefore, the application of the method described in our manuscript (using fluorescent markers) could benefit not only the users using cell lines with weak cell-cell adhesions, but also users with an interest on performing high-throughput screenings with viable bacteria or other substances that may aggregate. To illustrate this point, we provide four examples of studies in which scratch assays were used to test the influence of bacteria on wound repair:
i.Mohammedsaeed, W., Cruickshank, S., McBain, A. J. & O'Neill, C. A. Lactobacillus rhamnosus GG Lysate Increases Re-Epithelialization of Keratinocyte Scratch Assays by Promoting Migration. Scientific Reports 5, 16147 (2015).
ii.Fernandez-Gutierrez, M.M.; Roosjen, P.P.J.; Ultee, E.; Agelink, M.; Vervoort, J.J.M.; Keijser, B.; Wells, J.M.; Kleerebezem, M. Streptococcus salivarius MS-oral-D6 promotes gingival re-epithelialization in vitro through a secreted protein. Scientific Reports 7(1):11100, (2017).
iii.Laheij, A. M., de Soet, J. J., Veerman, E. C., Bolscher, J. G. & van Loveren, C. The influence of oral bacteria on epithelial cell migration in vitro. Mediators of inflammation 2013, 154532, doi:10.1155/2013/154532 (2013).
iv.Bhattacharya, R. et al. Effect of bacteria on the wound healing behavior of oral epithelial cells. PLoS ONE 9, doi:10.1371/journal.pone.0089475 (2014).

1.2I was not eluding to counting/detecting single cells in phase contrast images, which is terribly hard. Rather, I was proposing to follow the advancing monolayer edge over time, which is the standard measure for scratch experiments, or even using it to calculate the same parameters λ, μm and A. The authors can demonstrate the superiority of their approach over extracting the exact same parameters from the monolayer advancement rate with the experiment I proposed above (using less cohesive cells).

Author response: This point was addressed in the previous rebuttal where we explained the reasons why we think that the use of live-compatible fluorescent labels in combination with single-cell identification can provide a more accurate determination of wound healing kinetics than the classical measurement of following the monolayer edges over time. We agree with the reviewer that testing the performance of KREAP with a less cohesive cell line would have strengthen our manuscript but unfortunately, we do not have such a cell line in-house.

1.3Demonstrating KREAP robustness. (…) I was arguing that a second data-sets could be retrieved from another lab's previous publication to verify the applicability to diverse datasets. And that the lack of access to such data (through contacting a senior author of such a paper) imply that researchers are not staining their cells when performing scratch assays. I still think that this could be an important (but not necessary) addition to this manuscript.

Author response: In the revised manuscript, we proved the usability of our pipeline with an external image-series (lines 276-285, Supplementary Fig. S2). We demonstrate that the KREAP toolbox can be used to process images with a vertical scratch provided that you indicate in the index file the degrees needed to rotate the images to obtain a horizontal scratch. Furthermore, we were also able to validate the performance of our segmentation pipeline with a different cell line (MCF10A) expressing H2B-mCherry as a nuclear marker. Finally, we could validate the performance of the automatic identification of the scratch boundaries even when only one side of the scratch is visible obtaining excellent fits of the modified Gompertz model. The image-series were

provided by Mei Rosa Ng, the main author of the publication entitled: “Substrate stiffness regulates cadherin-dependent collective migration through myosin-II contractibility” published on 2012 in the Journal of Cell Biology.

2Measurements.

2.1The definition of λ, μm and A is now clear, but their biological interpretation is not clear to me. I do not find the following authors’ response helpful: “As we showed in ref. 19 (Fig. 2), the A parameter correlates with the maximum surface area (i.e. pixels) covered with cells (r = 0.68, P = 1.36 x 10^-6). Hence these parameters describe different aspects of the re-epithelialization kinetics and should therefore, not be correlated to each other”. This simply provides an example that A is correlated with the wound healing rate and nothing on different biological interpretation of these measures.

Author response: We apologize if we were not clear enough in our previous response. What we meant was that the λ, μm and A parameters describe different phases of the growth curve and therefore, we do not see how correlating the parameters to one another will provide more biological insight (it may actually confuse the interpretation; see also our answer under point 2.3). The μm parameter is an indicator of the wound healing rate, whereas the A parameter gives an indication of the ‘status’ of the wound (i.e. wound closure. To this end, the A parameter is correlated to the classical measurement of scratch assays that employ monolayer advancement (see point 1.2) which enables the calculation of the maximum wound closure achieved under a particular treatment after certain period of time. To test this, we normalized the A parameter values to that of the non-treated control and performed a Spearman correlation (n = 42) with the values obtained from calculating the relative wound closure as follows: 1 − [(# pixels in the scratch at time x)/(# pixels in the scratch at time 0) * 100] (also expressed relative to the non-treated control). The results showed a highly significant positive correlation between the two measurements (r = 0.68, P = 1.36 x 10^-6), providing evidence that the A parameter provides a ‘snapshot’ of the status of wound closure. This point was also clarified in the revised version of the manuscript in lines 193-205.

2.2I am not convinced that these parameters encode the “initial cellular responses to a bioactive substance” (see below) and the “subsequent activation/inhibition of cell proliferation” (much of the area covered in theses assays is usually explained by cell spreading rather than proliferation)

Author response: We agree with the reviewer that the area covered in a scratch assay can be explained by several processes including cell spreading, but area coverage is also influenced by enhancement of proliferation by the treatment. Depending on the agent, the treatment may accelerate proliferation (e.g. treatment with TGF-alpha or another growth factor) or inhibit proliferation (e.g. treatment with pathogenic Porphyromonas gingivalis or chemical inhibitors of proliferation). Since it is not always possible to exclude either spreading or proliferation a priori (and this may depend on the cell line used), we feel we should keep both explanatory mechanisms included.

2.3The authors’ response that in Fig. 6a we provide an example in which addition of a particular treatment initially induced rapid cell migration (represented by the high μm value), but during the course of the experiment, migration of cells into the scratched area ceased, resulting in a low A parameter value” is not demonstration as in the text this effect relates to experimental failure that led to cell death.

Author response: We would like to clarify that the values obtained with the treatment in Fig. 6a are not due to experimental failure. On the contrary, this was a biologically relevant result providing evidence that this particular bacterial preparation causes an initial stimulatory response in the cells which was reflected by a high migration rate (μm parameter). Over time, cell migration ceased, leading to an unresolved wound and therefore, a low A value. This is an example in which the m and A parameters are negatively correlated. Hence, a high μm value does not necessary imply a high A value.

2.4I would argue that the μm could be seen as a more accurate measure than the standard “wound healing rate” (how fast the free area is covered by cells) because it

corrects for the lag and stationary phase. For large scratches, I expect A to be highly correlated with μm (at least until the cell density is dramatically reduced). For a given condition, I expect the stationary phase to start earlier for smaller scratches, as the density increases in the "wound" area. Does A encode any additional biological-relevant parameter beyond μm and the wound size?

Author response: As explained before in points 2.1 and 2.3, the A parameter provides a snapshot of the status of the wound and therefore is a separate measurement from the μm parameter. Application of the modified Gompertz model requires standardization of the wound size to minimize its variation (see Ref. 17 of the revised manuscript for more information on this). We included this remark in our revised manuscript on lines # 116-123. The wound size could be optimized using dedicated scratching tools such as the HTScratcher (Peira, BE) or defined cell-free gap inserts (ibidi, DE), among others. This eliminates variation in the wound size as a possible confounding factor.

2.5Can the authors explain whether λ has a biological interpretation in the setting of a scratch assay? Could an example be provided were λ is systematically altered without affecting the other parameters? I am concerned that λ might encode a technical parameter of the experimental setting (perhaps "batch effect").

Author response: As explained in our manuscript (lines # 196-201) and in our previous study (Ref. 17 of the revised manuscript) the λ parameter (expressed in minutes) was consistently characterized by small to negative values, indicating that the migration process was initiated before the imaging acquisition started. Therefore, we concluded that in the cell lines tested, the λ parameter has a limited biological meaning, but its calculation is essential to obtain an accurate approximation of the μm and A parameters.

2.6The authors should (1) better explain the biological interpretation of the parameters they extract; (2) provide data to support these explanations, this includes correlations between the different parameters and/or different experimental conditions that alter one parameter independently of the other (modularity). If the authors intend to argue that the μm and A encode" subsequent activation/inhibition of cell proliferation", this has to be shown by verifying how much of the increased area covered by the cells is explained by spreading and how much by proliferation. This is a critical point in my review.

Author response: (1) We hope that we provided the reviewer with a clearer explanation of the biological interpretation of the parameters we extract in points 2.1, 2.4, and 2.5 of this rebuttal. (2) Please refer to point 2.3 for an example on modularity. We regret the misunderstanding in the previous rebuttal, but we do not intend to argue that the μm and A parameters encode for subsequent activation/inhibition of cell proliferation, rather we interpret these parameters as useful to describe re-epithelialization kinetics, which encompasses the processes of cell migration (cell spreading) and/or proliferation.

2.7My request to discuss pros and cons compared to alternative approaches was in the context of comparing the extracted measurements and not in the context of open/commercial software. I still believe it is necessary and important to put these measures in context with other available solutions in a discussion highlighting the cons and pros of the measures that extract via KREAP.

Author response: As mentioned in our manuscript (lines # 58-68) there are different solutions for quantification of wound-healing assays. However, KREAP is specifically designed for high-throughput screenings and has the advantage over the other solutions that it is an open-source platform in Galaxy that provides quantitative re-epithelialization measurements using a novel modelling approach based on the quantification of single-cell infiltration.

Other corrections and suggestions:
1.Data availability. GigaDB was built for the purpose of making GigaScience publication data publicly available.

Powered by Editorial Manager® and ProduXion Manager® from Aries Systems Corporation

Author response: As we outlined before, we will address this point once our manuscript is accepted for publication. At the moment, our data is available in our homepage and in the Github repository.

2.Line # 78: Scratch is an assay for migration, quantifying cell death and migration together is esoteric and KREAP (as currently implement) do not provide the means to quantify it.

Author response: We agree with the reviewer and decided to remove that statement from the revised manuscript.

3.Line # 146: “the difference in intensity is used to separate merged objects into individual ones” – how? Please provide more details (it sounds like a watershed algorithm?)

Author response: As indicated by the reviewer, merged objects are separated by a watershed algorithm. This was also specified in the revised version of the manuscript (lines # 155-156).

4.Line # 157 -159: Automatic identification of the scratch boundaries. The description “finds the largest empty area by measuring the frequency of cells” is not sufficiently detailed. How exactly is the area determined? More importantly, this implementation assumes a very specific setting of the scratch assay – a vertical cell free area in the middle of the field of view. This limitation (e.g., the software is not suitable for circular scratches; images of horizontal scratches must be rotated; would it work for scratches were the other side is not in the field of view?) must be mentioned in the text and in the software user guides!

Author response: We apologize to the reviewer for not providing a clear explanation. Here we include a diagram (also added as Supplementary Fig. S3 and explained in the revised manuscript lines 167-173) to make this point clear.

The software first searches for the largest cell-free area (main scratch), indicated by the blue arrow. Then, it searches for smaller gaps up and down of the main scratch (represented in red, yellow, and purple). These smaller gaps are then added up to the main scratch resulting in the identification of the scratch boundaries (represented with the green arrow).

In the revised version of KREAP, we also address the second point of the reviewer: we provide an option to rotate (clockwise) images directly in the index file by indicating how many degrees would be required to obtain a horizontal cell-free area. In this way, KREAP is able to process vertical and horizontal scratches (See Supplementary Fig. S2). In addition, we show that KREAP is suitable to process images with scratches in which only one side of the scratch is in the field of view (see Supplementary Fig. S2). We have not tested if KREAP is suitable for circular scratches. These points are now mentioned in the main text of the revised manuscript (lines 123-127) and are updated in the documentation of the software and in our homepage (https://erasmusmc-bioinformatics.github.io/KREAP/).

5.Line # 172: the parameters , m and A are mentioned but defined only later at line # 179. I would suggest to present them first at the location they are defined, otherwise it could be cryptic and confusing.

Author response: We appreciate that the reviewer pointed this out and we adjusted our manuscript accordingly to make it clearer.

6.Lines # 190-192: “Importantly, we have shown that the A parameter is correlated to the maximum surface area in pixels covered by cells”. Why is this important? Isn't it trivial? I suggest to exclude this text.

Author response: As suggested by the reviewer, we decided to leave this statement out of the revised manuscript.

7.Line # 193: Would be insightful to explicitly provide the physical/biological

interpretation of the ‘performance value’ (µm*A), which is not obvious to me – is it a standard measure in a different assay (then please provide a reference or at least an explanation)? Also, this measure is not used throughout the manuscript – why mention it then?

Author response: We agreed with the reviewer that as we do not refer to this measurement anywhere in our manuscript, we decided to leave it out of the revised version.

8.Line # 203-205: I do not see the point in this.

Author response: We removed lines 203-205 from the main text as well as panel (a) in Fig. 4.

9.Line # 213-217: Please report the fold change in addition to the p-value.

Author response: As indicated by the reviewer, we now provide the fold change in addition to the p-value (lines # 219-226).

10.Line # 219: Please describe the sub-dataset. How large is it? What was the criteria to select experiments from the full dataset?

Author response: We apologize for the confusion, but we actually used a complete dataset from the published study that consisted of a total of 214 image-series. This point was also adjusted in the revised manuscript in line # 227 and line # 230-231.

11.Line # 245-247: “detrimental effects” are not defined in the main text (should be independent of the figure legend).

Author response: In the revised manuscript, we defined the identification of adverse effects in the main text as well as in the legend (lines 253-264).

12.Line # 257-259: Please provide guidelines on how to determine if a given experiment is faulty (dead cells) or a strong effect caused by a perturbation? Especially when the software is not providing an image of the cells. Is there a way to decide based on the curves?

Author response: The software provides the raw images, segmentation output, and re-epithelialization curves (when successfully modelled) of each well. In addition, we implemented a “flagging” system in the software that helps the user to identify strong inhibitory or detrimental effects caused by a particular treatment. These flags or warnings are described in lines 262-264.

13.Legend on Figure 4: no reference to panel (b)

Author response: We appreciate the reviewer for pointing this out. We adjusted this point accordingly in the revised manuscript.

14.The “analysis image (right) does not fit in size to the other two and cannot be compared.

Author response: The raw images are re-scaled in the revised version of KREAP to match the segmentation output.

15.Figure 1: The “modify index” is not explained in the figure legend nor the main text. Is it just switching to the next well?

Author response: The modify index can be used to exclude a specific well from the analysis (e.g. when there is a technical error). This function is explained in the revised manuscript in lines 139-141.

16.Figure 2: It is hard to compare the input and output due to resizing – can this be fixed?

Powered by Editorial Manager® and ProduXion Manager® from Aries Systems Corporation

	Author response: The raw images are re-scaled in the revised version of KREAP to match the segmentation output.<br><br>17.Figure 3 legend: make the first sentence bold to fit the other legends.<br><br>Author response: We thank the reviewer for this remark and changed the first sentence to bold to match the other legends.<br><br>18.The link to the github repository is broken (I could not get to the point of entering the username and password)<br><br>Author response: We apologise for the confusion, but the link of the Github repository is working. You need to provide the username (erasmusmc-review) and the password (erasmusmcreview123) on the top of the page (see red square below).<br><br>19.Methods should include detailed description of the algorithmic pipeline. Some of the main text could be transferred (and elaborated) in the Methods.<br><br>Author response: The algorithms can be found in the Github.
**Additional Information:**	
**Question**	**Response**
Are you submitting this manuscript to a special series or article collection?	No
**Experimental design and statistics**<br><br>Full details of the experimental design and statistical methods used should be given in the Methods section, as detailed in our Minimum Standards Reporting Checklist. Information essential to interpreting the data presented should be made available in the figure legends.<br><br>Have you included all the information requested in your manuscript?	Yes
**Resources**<br><br>A description of all resources used, including antibodies, cell lines, animals and software tools, with enough information to allow them to be uniquely identified, should be included in the Methods section. Authors are strongly encouraged to cite Research Resource Identifiers (RRIDs) for antibodies, model organisms and tools, where possible.<br><br>Have you included the information requested as detailed in our Minimum Standards Reporting Checklist?	Yes
**Availability of data and materials**	Yes

All datasets and code on which the conclusions of the paper rely must be either included in your submission or deposited in publicly available repositories (where available and ethically appropriate), referencing such data using a unique identifier in the references and in the "Availability of Data and Materials" section of your manuscript.<br><br>Have you have met the above requirement as detailed in our Minimum Standards Reporting Checklist?	

Powered by Editorial Manager® and ProduXion Manager® from Aries Systems Corporation

Manuscript

Click here to view linked References

Click here to access/download;Manuscript;KREAP-An automated Galaxy Platform_revised_v2.docx

KREAP: An automated Galaxy Platform to Quantify *in vitro* Re-Epithelialization Kinetics

Marcela M. Fernandez-Gutierrez[1,2$], David B.H. van Zessen[3$], Peter van Baarlen[2], Michiel Kleerebezem[1,2], Andrew P. Stubbs[3*]

[1]TI Food and Nutrition, Nieuwe Kanaal 9-A, 6709 PA, Wageningen, The Netherlands.

[2]Host-Microbe Interactomics, Animal Sciences Group, Wageningen University & Research, De Elst 1, 6708 WD, Wageningen, The Netherlands.

[3]Department of Bioinformatics, Erasmus University Medical Centre, Wytemaweg 80, 3015 CN, Rotterdam, The Netherlands.

$ - Both authors contributed equally

*** - Correspondence**

Andrew P. Stubbs: a.stubbs@erasmusmc.nl, ORCID: 0000-0001-9817-9982

Author email addresses:

Marcela M. Fernandez-Gutierrez: marcela.fernandez@wur.nl, ORCID: 0000-0001-5314-9659

David B.H. van Zessen: d.vanzessen@erasmusmc.nl

Peter van Baarlen: peter.vanbaarlen@wur.nl, ORCID: 0000-0003-3530-5472

Michiel Kleerebezem: michiel.kleerebezem@wur.nl, ORCID: 0000-0001-8552-2235

Abstract

Background: *In vitro* scratch assays have been widely used to study the influence of bioactive substances on the processes of cell migration and proliferation that are involved in re-epithelialization. The development of high-throughput microscopy and image analysis has enabled scratch assays to become compatible with high-throughput research. However, effective processing and in-depth analysis of such high-throughput image-datasets is far from trivial and requires integration of multiple image processing and data extraction software tools.

Findings: We developed and implemented a Kinetic Re-Epithelialization Analysis Pipeline (KREAP) in Galaxy. The KREAP toolbox incorporates freely available image analysis tools and automatically performs image segmentation and feature extraction of each image series, followed by automatic quantification of cells inside and outside the scratched area over time. The enumeration of infiltrating cells over time is modelled to extract three biologically relevant parameters that describe re-epithelialization kinetics. The output of the tools is organized, displayed, and saved in the Galaxy environment for future consultation.

Conclusions: The Galaxy KREAP toolbox provides an open-source, easy-to-use, web-based platform for reproducible image processing and data analysis of high-throughput scratch assays. The KREAP toolbox could assist a broad scientific community in the discovery of compounds that are able to modulate re-epithelialization kinetics.

Keywords: Galaxy, scratch assay, high-throughput, cell migration, re-epithelialization, image analysis, workflow, modelling.

Findings

Background

Cell migration and proliferation play an essential role in a variety of physiological processes, including embryogenesis, angiogenesis, skin and intestinal renewal, and wound repair [1, 2]. Deregulation of these processes can contribute to the development and progression of multiple diseases such as osteoporosis, rheumatoid arthritis, vascular disease and cancer [1]. Therefore, studying the molecular mechanisms underlying the processes of cell migration and proliferation is not only important for obtaining fundamental scientific insight, but it is also essential for the development of effective therapeutic strategies that could modulate these processes when they have become dysregulated.

The *in vitro* scratch assay is a well-established and widely used method to study cell migration and proliferation [3-5]. The assay is based on the introduction of a scratch into a confluent epithelial

cell monolayer to create a cell-free area. Cells migrate and proliferate into the site of injury in a process known as re-epithelialization [6]. This process is typically monitored by acquisition of images at the beginning and at one or more fixed time points during re-epithelialization. The image series obtained for a particular treatment is then compared to that of the non-treated control to determine the treatment's modulatory capacity in the healing process. The development and constant improvement of image segmentation algorithms over the past decades have enabled the transition from manual quantification of the scratch area to automated analysis which is compatible with high-throughput screenings [7-9].

CellProfiler [10] and ImageJ [11] are freely available image analysis software tools that allow scientists with limited programming skills to conduct efficient image segmentation and feature extraction of high-throughput image datasets. However, scripting and parsing of data are often necessary to optimally use the capacities of these tools, requiring programming skills that many biologists do not have. Commercial software such as FCS Express Image Cytometry (De Novo Software, CA, USA) [12], Image-Pro Premier (Media Cybernetics, WA, USA), among others, provide alternatives to ease data analysis, but require the purchase of licenses. TScratch was the first open-source application exclusively designed to perform automated analyses of scratch assay's analyses by determining the percentage of open wound area after introducing a scratch in a cell monolayer, but lacks the ability to extract real-time kinetic data [13]. More recently, CellMissy was developed as an open-source software to determine the area (μm^2) of wound closure over time as well as the mean collective cell migration velocity (μm/h) for a given condition [14]. Nevertheless, the biological properties of certain cells may render these calculations challenging. For instance, poorly adherent cells (e.g. FHs-74 small intestinal cells) do not migrate collectively, but detach and migrate individually in uneven patterns, making wound area measurements inaccurate [15]. Thus, there is still a need for an open-source platform based on single-cell recognition, which could integrate different validated tools for image segmentation,

visualization, and data analysis of the processes of cell migration and proliferation involved in wound repair.

We developed and implemented a Kinetic Re-Epithelialization Analysis Pipeline (KREAP) in Galaxy (https://galaxyproject.org/) (Galaxy , RRID:SCR_006281)[16] to deliver a web browser-based application for quantitative analysis of *in vitro* scratch assays based on single-cell recognition. The user needs to download and install a virtual machine (VM) containing a fully operational KREAP Galaxy installation. Once the VM is installed, the user uploads the images from a multi-well plate experiment into the VM, together with its corresponding index file, and presses the *Execute* button to automatically perform single-cell segmentation and feature extraction across all images. Enumeration of cells inside and outside the scratched area is also carried out automatically over the time series. Based on the number of cells infiltrating the scratch over time, KREAP extracts three biological comprehensive parameters that describe the kinetics of re-epithelialization. In addition, the user's history is saved in the VM for future consultation and the results can be easily shared with other users by downloading the history of multiple experiments. Taken together, we provide a platform that enables reproducible data processing and analysis of high-throughput scratch assays –from raw images to re-epithelialization kinetics– that facilitate screenings of substances that may influence re-epithelialization.

Implementation

The scratch assay analysis workflow was developed within our own laboratory [17] (also see Methods) and involved a multi-software approach to acquire images, perform image analysis, visualize extracted data, and model re-epithelialization kinetics based on the enumeration of cells migrating into the scratch area over time. CellProfiler (http://cellprofiler.org/) was used in the original workflow and implemented in the KREAP toolbox (version 2.2.0) to perform automated image segmentation and feature extraction of image series. FCS Express 4 Plus (De Novo Software, CA, USA) was originally used to relate the features extracted by CellProfiler back to the

raw images and to enumerate the cells infiltrating the scratched area over time [17]. Since FCS Express 4 Plus requires the purchase of a license, we developed and implemented an R [18] script, now part of the KREAP toolbox, that can automatically recognize the scratch boundaries and determine the number of cells inside and outside the scratch over time. Modelling of re-epithelialization kinetics was programmed in R and also implemented into the KREAP toolbox workflow. The workflow is provided in a fully operational Galaxy installation inside of a VM that can be retrieved from the KREAP homepage (https://erasmusmc-bioinformatics.github.io/KREAP/). The VM can be executed using the freely available Oracle VM VirtualBox (https://www.virtualbox.org/wiki/Downloads), which is compatible with a number of host operating systems, including: Linux, Windows and Mac OS. For detailed installation instructions, visit our homepage (https://erasmusmc-bioinformatics.github.io/KREAP/). The source code is available as open-source via the GitHub repository.

Experimental setup and data acquisition

KREAP was designed to perform single-cell segmentation and therefore, nuclear labelling using live-cell compatible dyes or alternatively, using stably transduced cell lines expressing fluorescent nuclear markers is highly recommended to ensure accuracy of the image analysis pipeline. We also encourage scientists to verify the normal response of transduced or fluorescently labelled cells to specific stimuli (see **Supplementary Fig. S1**). Importantly, the automatic recognition of the scratch boundaries as well as the quantitative determination of the re-epithelialization kinetics implemented in KREAP, require standardization of the scratch's shape and size, to minimize variation between wells. This can be accomplished by using dedicated high-throughput scratching tools such as the HTScratcher (Peira, BE; also see Methods) or defined cell-free gap inserts like the ones supplied by ibidi (ibidi, DE). The KREAP toolbox can process horizontal scratches or cell-free areas where only one scratch edge or both edges are visible in the field of view (see **Supplementary Fig. S2**). Furthermore, vertical or diagonal scratches can be processed in the KREAP workflow provided that the user indicates in the index file the number of degrees needed

to rotate the images (clockwise) to obtain horizontal scratches. Images of the same field of view must be acquired at fixed intervals until the scratches in the wells treated with a positive control are fully resolved.

Analysis workflow and data handling

Images (.tif) derived from a multi-well plate experiment must be converted into grayscale, organized in consecutive order, placed in folders by well, and indexed accordingly in a separate file. An exemplary index (.txt) and input files are provided at the KREAP homepage (https://erasmusmcbioinformatics.github.io/KREAP/file_formats). The folders containing the image series of each well should be compressed into a .zip file and uploaded into the Galaxy history via the "Get data" tool together with its corresponding index file (**Fig. 1**). The KREAP toolbox, consisting of the "Image Analysis" and "Data-Modelling" tools, can be executed within the Galaxy platform. At the end of each processing step, the results are provided as HTML and stored in the Galaxy history for future consultation. If desired, the graphs (.png) and tables (.txt) generated by both tools can be downloaded as a compressed file (.zip) by clicking the *Download* icon in the Galaxy history. When necessary (e.g. when there is a technical error), it is possible to exclude specific wells from the analyses by clicking the link "Make new index file", without having to upload the modified datafiles into the Galaxy history.

Image Analysis tool

Once the input files are uploaded into the Galaxy history, the "Image Analysis" tool can be executed (**Fig. 1**). The tool uses an image segmentation pipeline developed in the open-source software CellProfiler 2.2.0 (http://cellprofiler.org/). The individual modules contained in the pipeline carry out automated extraction of cellular features in every image. An illumination function is calculated in the first segmentation module by finding the minimum pixel intensities in blocks (e.g. block size 5-20 pixels) across each image and applying a Gaussian filter as smoothing method [19]. In the second module, the calculated illumination function is applied to the raw

image by subtraction, resulting in better contrast between the fluorescent nuclei and the background. Identification of primary objects is defined in the third module as objects within a specified diameter range (in pixels), depending on the cell type used. Identification of primary objects is performed by applying a global threshold strategy in combination with the Otsu algorithm [20] which calculates a single threshold value that classifies pixels above the threshold as foreground and below the threshold as background. Because objects tend to be brighter towards the interior than towards the edges, a watershed algorithm is used to separate merged objects into individual ones [21]. The last module extracts phenotypic features (e.g. size, eccentricity and mean intensity) from each object as well as their x- and y-coordinates within the image. For optimal image segmentation results, the user can adjust the parameters for illumination correction (i.e. block size) and object identification (i.e. minimum and maximum object diameter size) for each well directly in the index file. However, for objective comparison, it is recommendable to use the same parameters across wells seeded with the same cell type.

The graphical interface of Galaxy provides the user with an overview of each well within the multi-well plate (**Fig. 2**). The location of the identified primary objects is visualized in an interactive plot that uses a slider to move through images over time. A compare function is provided to visually evaluate the performance of the image segmentation pipeline by comparing its output with the raw image. Automatic identification of the scratch boundaries was accomplished using a customized R script that detects the largest cell-free area in each well by measuring the cell frequency on the Y axis at the beginning of the assay (see **Supplementary Fig. S3** for a schematic view). To avoid underestimation of the scratch size that could result if single cells are left within the scratched area, the algorithm searches for smaller gaps up- and downwards of the largest cell-free area. When smaller gaps are identified, these are added up to the largest cell-free area, resulting in the final identification of the scratch boundaries. The total number of cells is determined in each image over time and classified into objects inside or outside of the scratched area (**Fig. 2**). The image segmentation results are stored in the Galaxy history and can be accessed

by the user in the future. Furthermore, the cellular features extracted by CellProfiler and the enumeration of cells inside or outside of the scratched area can be easily downloaded via the links provided in the output.

Data-Modelling tool

The output derived from the image analysis can be used in the "Data-Modelling" tool to extract three biologically relevant parameters that describe the kinetics of re-epithelialization (**Fig. 1**). To calculate the parameter values, the time interval between images must be entered in the index file before uploading the file into the Galaxy history. The enumeration of cells infiltrating the scratched area over time consistently results in a sigmoidal curve similar to the ones obtained with bacterial growth curves that are characterized by a lag phase, an exponential phase, and a stationary phase (**Fig. 3**) [17]. The modified Gompertz function has been successfully used to model bacterial growth to estimate three biologically relevant parameters that mathematically describe the different phases of growth [22]. We developed and implemented an R script to fit the modified Gompertz function through the re-epithelialization measurements using a nonlinear least squares regression in combination with the Levenberg-Marquardt algorithm to reduce the sum of the squares of the errors between the modelled and measured data points in an iterative manner [17]. In this way, we were able to obtain excellent fits that were characterised by R^2 values close to 1 and low root-mean-square error (RMSE) values. The modified Gompertz function describes the re-epithelialization kinetics for each image series through the estimation of the lag time (λ; minutes), the repair rate (μ_m; cells/minute), and the maximum number of cells within the scratched area at the plateau of the re-epithelialization curve (A; number of cells). The λ parameter represents the time required for cells to start migrating into the scratched area. For some cell lines (e.g. Ca9-22), the lag time can be very brief and the migration process may start even before image acquisition takes place [17]. In those cases, the λ parameter is estimated to be zero or may have negative values in which case, the biological relevance of this parameter to the kinetic description is negligible. Nonetheless, the calculation of the λ parameter remains essential

for obtaining an accurate fit of the model. The μ_m parameter is an indicator of repair rate (cells/min), whereas the A parameter gives an indication of the 'status' of wound closure. To this end, the A parameter is correlated to the classical measurement of scratch assays that employ monolayer advancement to calculate the maximum wound closure achieved under a particular treatment after certain period of time [17]. The parameter values obtained for each replicate condition can be used in a screening to identify substances that stimulate or attenuate wound repair when compared with the non-treated controls.

Anticipated results

As an exemplary dataset, gingival epithelial cells (Ca9-22) were seeded in 96-well plates and incubated overnight to obtain a confluent cell monolayer. During the last 20 minutes of the starvation period (i.e. incubation with FCS-free DMEM for 2 hours), nuclei were stained with 2 μg/ml Hoechst 33342. After 2 hours of starvation, the cell monolayers were scratched with the HTSScratcher to create an artificial wound in each well. The wells were then washed twice with phosphate buffered saline (PBS) to remove the nuclear staining-solution and detached cells. Human transforming growth factor α (hTGFα) acted as a mitogenic and mobility factor (**Fig. 4a)** through the activation of the epidermal growth factor receptor (EGFR) [23]. In contrast, addition of chemical inhibitors of p38 and MEK1/2 phosphorylation led to suppression of cell migration and ERK1/2-mediated proliferation (**Fig. 4a)**, respectively [24, 25]. Calculation of the kinetic parameters describing re-epithelialization kinetics showed that treatment with TGFα resulted in more than a 2-fold increase in the repair rate (P = 0.015) when compared with the untreated cells (**Fig. 4b**). Likewise, stimulation with TGFα resulted in a 1.5-fold increase in the numbers of cells inside the scratched area in comparison with the non-treated control (P = 0.004) (**Fig. 4c**). Inversely, treatment with the solution containing p38 and MEK1/2 inhibitors resulted in a 2- and 3-fold decrease in the repair rate and in the number of infiltrating cells (P = 0.0008), respectively, when compared to the non-treated control during the scratch assay (**Fig. 4b** and **4c**).

Validation of the KREAP toolbox with a published dataset

The performance of the KREAP toolbox was evaluated in comparison with a previous published workflow in which we employed multiple software tools and a manual determination of the scratch boundaries [17]. Using the KREAP toolbox in the Galaxy platform, we processed a dataset from that study consisting of 214 image-series. Data processing was performed on a Windows 7 desktop with an Intel® Core™ i7-3970X processor with 4 cores at 3.50 GHz and 4 GB of RAM. Both KREAP and the original workflow use CellProfiler for automated image segmentation and feature extraction. However, in the original workflow, data visualization and enumeration of infiltrating cells over time was determined using the licensed FCS Express 4 Plus (De Novo Software, CA, USA) software tool. The location of the identified objects at the beginning of the assay was plotted in a scatterplot after which a rectangular gate was manually placed on the scratched area and a batch process was setup to record the number of infiltrating cells over time for each well [17]. FCS Express 4 Plus was replaced in the KREAP Image Analysis tool by an in-house developed and customized R script that automatically recognizes the boundaries of the scratch. To assess the performance of the KREAP Image Analysis tool in comparison with the manual determination of the scratched area used in our original study [17], the kinetic parameter values (i.e. μ_m and A) obtained with both workflows were compared by a Pearson correlation analysis. For both the μ_m and A parameters, the analysis identified a strong correlation between the values obtained in the previous study and those obtained with the KREAP toolbox with correlation values of 0.85 ($P < 0.0001$) and 0.83 ($P < 0.0001$), respectively (**Fig. 5**). These results illustrate the accuracy of the KREAP toolbox which eliminates user's manual data-handling, but also significantly reduces the time required for performing the analysis. For example, processing of a multi-well plate experiment consisting of 60 wells and 16 timepoints (960 images in total) with the original workflow would typically take around 3 to 4 hours for an experienced user to complete. In contrast, the KREAP toolbox can perform the complete analysis –from raw images to

quantification of re-epithelialization kinetics– in less than 30 minutes with the additional advantage that it does not require computer programming skills.

Identification of adverse effects on re-epithelialization

Although the modified Gompertz function is used to model positive sigmoidal growth curves, identification of adverse effects on re-epithelialization kinetics is possible through the inspection of the curves generated with the measured and modelled data points. In this example (**Fig. 6**), Ca9-22 cells were exposed to the periodontal pathogen, *Porphyromonas gingivalis,* which adversely affects re-epithelialization [17, 26, 27]. As indicated by the high repair rate value (μ_m = 7.9 cells/min), the gingival cells migrated rapidly into the scratched area shortly after exposure to this bacterium. However, after reaching a plateau at 150 min, exposure to this bacterium leads to induction of cell death and subsequently, a decline in the number of infiltrated cells over time, resulting in a low A parameter value and an unresolved wound. Modelling of the re-epithelialization curve, resulted in a poor goodness of fit (R^2 = 0.66) that could be easily recognized by the KREAP Data-Modelling flagging system, which highlights curves with R^2 values lower than 0.9 to be inspected by the user.

Robustness of the KREAP toolbox and final remarks

A key aspect of high-throughput microscopy research is to convert the raw images into quantitative and biologically comprehensive data. This step typically requires multiple software tools, programming skills or purchase of costly software to aid in the image processing and data analysis. The KREAP toolbox integrates multiple validated tools in Galaxy that enable automatic image segmentation, visualization and data analysis of high-throughput screenings using scratch assays. The implementation of the KREAP toolbox in Galaxy also provides an open-source web-based platform that allows scientists that lack advanced programming skills to perform the complete analysis starting with the raw images and ending with the quantified kinetics based on single-cell recognition. Moreover, the graphical user interface of Galaxy provides an easy-to-use

environment that organizes, displays, and saves the results of each experiment as part of the user's history.

To further demonstrate the robustness and versatility of the KREAP toolbox, we tested it with images that were generously provided by Dr. Ng and Dr. Brugge (Hardvard Medical School, Boston, MA, USA) and that were part from a collective migration study [28]. We show that the KREAP toolbox can be used to process images with vertical scratches by indicating in the index file that the images should be rotated by 90°. Furthermore, we were able to validate the performance of the "Image Analysis" tool using a different cell line (MCF10A) that expresses H2B-mCherry as a nuclear marker, with images in which only one edge of the scratch is visible in the field of view (**Supplementary Fig. S2**). The "Data Modelling" tool yielded an excellent fit (R^2 = 0.998) with a very low root-mean square error (RMSE = 12.93), indicating a high accuracy in the estimation of the parameter values describing re-epithelialization (**Supplementary Fig. S2**).

Taken together, the KREAP toolbox in Galaxy provides an "end to end" integrated analytical high-throughput screening platform that is useful for scientists who are interested in the discovery and mechanistic analysis of compounds that can modulate re-epithelialization kinetics.

Methods

Cell line

Gingival epithelial cells (Ca9-22) were purchased from the National Institute of Biomedical Innovation JCRB Cell Bank, Osaka (JCRB Cat# JCRB0625, RRID:CVCL_1102). Ca9-22 cells were cultured in Dulbecco's Modified Eagle Medium (DMEM) containing Glutamax (Gibco, Invitrogen, Paisley, UK), 10% fetal calf serum (FCS), 100 U/ml penicillin and 100 μg/ml streptomycin (Sigma-Aldrich, MO, USA). Cells were cultured at 37°C in a humidified atmosphere containing 5% CO_2 and passaged when a 70% confluency was reached.

Scratch assay and image acquisition

The experiment was carried out as described in [17]. Briefly, Ca9-22 cells were seeded in 96-well plates (BD Falcon™, Corning, NY, USA) at a density of 3.5×10^4 cells/well and incubated over night to obtain a confluent cell monolayer. The next day, cells were starved in FCS-free DMEM for 2 hours to decrease the basal cell proliferation. During the last 20 minutes of starvation, nuclei were stained with FCS-free DMEM containing 2 μg/ml Hoechst 33342. Following the starvation, equally sized scratches (0.3 x 2 mm) were introduced in the cell monolayers with the HTSScratcher (Peira, Antwerpen, BE). After washing the cells twice with phosphate buffered saline (PBS), the treatments (for details see [17]) were added into the wells in a randomized manner using three technical replicates. The positive control consisted of 4 ng/ml human transforming growth factor alpha (TGFα; R&D Systems, MN, USA); a combination of inhibitors of p38 (SB203580; Cell Signaling Technology, MA, USA) and MEK1/2 (U0126, Cell Signaling Technology) at a concentration of 10 μM each, served as negative control. FCS-free DMEM was used as non-treated control. The overall quality of each run of the 96-well based assay was assessed by calculation of the Z' factor, which establishes a dynamic range between the positive and negative control values [29]. Images were acquired using the BD Pathway 855 Bioimaging System (BD Biosciences, CA, USA) under controlled temperature and atmosphere (37°C and 5% CO_2). Fluorescent images were acquired using an excitation filter of 350 nm. The BD Pathway platform was programmed to acquire the same field of each well every 20 minutes for 5 hours using a 4x objective.

Availability and Requirements

- Project name: KREAP (Kinetic Re-Epithelialization Analysis Pipeline)
- Project home page: https://erasmusmc-bioinformatics.github.io/KREAP/
- Operating system: KREAP was developed in Linux and can be executed in Unix-based operating systems, Microsoft Windows or Mac OS X.
- Programming languages: Python, R programming language
- License: Freely available under the MIT open source license
- Any restriction to use as non-academic: none

- Virtual machine accessibility: via the KREAP homepage and GitHub repository

Availability of supporting data

The dataset supporting the results of this article is available at the KREAP homepage (Kinetic Re-Epithelialization Analysis Pipeline) and in the *GigaScience* GigaDB repository[30].

Competing interests

The authors declare that they have no competing interests.

Authors' contributions

MMFG, DHBvZ, PvB, MK and AS conceived the study and contributed to writing the first draft of the manuscript. MMFG performed the scratch assays and carried out the validation analyses. DHBvZ and AS developed and implemented the analyses workflow. All authors contributed to editing the final manuscript. All authors read and approved the final manuscript.

Acknowledgements

We thank Dr. Mei Rosa Ng and Prf dr Joan S. Brugge from the Department of Cell Biology at Harvard Medical School (Boston, Massachusetts, USA) for providing us with a previously published dataset to test the robustness of the KREAP toolbox.

References

1. Ridley AJ, Schwartz MA, Burridge K, Firtel RA, Ginsberg MH, Borisy G, et al. Cell Migration: Integrating Signals from Front to Back. Science. 2003;302(5651):1704-9. doi: 10.1126/science.1092053.
2. Friedl P, Gilmour D. Collective cell migration in morphogenesis, regeneration and cancer. Nat Rev Mol Cell Biol. 2009;10(7):445-57.
3. Liang CC, Park AY, Guan JL. In vitro scratch assay: a convenient and inexpensive method for analysis of cell migration in vitro. Nature protocols. 2007;2(2):329-33. doi: 10.1038/nprot.2007.30. PubMed PMID: 17406593.
4. Oudhoff MJ, Van Den Keijbus PAM, Kroeze KL, Nazmi K, Gibbs S, Bolscher JGM, et al. Histatins enhance wound closure with oral and non-oral cells. J Dent Res. 2009;88(9):846-50. doi: 10.1177/0022034509342951.
5. Mohammedsaeed W, Cruickshank S, McBain AJ, O'Neill CA. Lactobacillus rhamnosus GG Lysate Increases Re-Epithelialization of Keratinocyte Scratch Assays by Promoting Migration. Scientific Reports. 2015;5:16147. doi: 10.1038/srep16147. http://www.nature.com/articles/srep16147#supplementary-information.
6. Schäfer M, Werner S. Transcriptional Control of Wound Repair. Annual Review of Cell and Developmental Biology. 2007;23(1):69-92. doi: 10.1146/annurev.cellbio.23.090506.123609.
7. Yarrow JC, Perlman ZE, Westwood NJ, Mitchison TJ. A high-throughput cell migration assay using scratch wound healing, a comparison of image-based readout methods. BMC biotechnology. 2004;4:21. doi: 10.1186/1472-6750-4-21. PubMed PMID: 15357872; PubMed Central PMCID: PMC521074.

8. Zordan MD, Mill CP, Riese DJ, 2nd, Leary JF. A high throughput, interactive imaging, bright-field wound healing assay. Cytometry Part A : the journal of the International Society for Analytical Cytology. 2011;79(3):227-32. Epub 2011/11/03. doi: 10.1002/cyto.a.21029. PubMed PMID: 22045642; PubMed Central PMCID: PMC3306835.
9. Simpson KJ, Selfors LM, Bui J, Reynolds A, Leake D, Khvorova A, et al. Identification of genes that regulate epithelial cell migration using an siRNA screening approach. Nat Cell Biol. 2008;10(9):1027-38. doi: http://www.nature.com/ncb/journal/v10/n9/suppinfo/ncb1762_S1.html.
10. Lamprecht MR, Sabatini DM, Carpenter AE. CellProfiler™: Free, versatile software for automated biological image analysis. BioTechniques. 2007;42(1):71-5. doi: 10.2144/000112257.
11. Schindelin J, Rueden CT, Hiner MC, Eliceiri KW. The ImageJ ecosystem: An open platform for biomedical image analysis. Molecular Reproduction and Development. 2015;82(7-8):518-29. doi: 10.1002/mrd.22489.
12. Software DN. A Flow Cytometry Analysis Environment for Image Cytometry Data. Available from: https://www.denovosoftware.com/site/Image-Overview.shtml. 6 ed.
13. Geback T, Schulz MM, Koumoutsakos P, Detmar M. TScratch: a novel and simple software tool for automated analysis of monolayer wound healing assays. BioTechniques. 2009;46(4):265-74. Epub 2009/05/20. doi: 10.2144/000113083. PubMed PMID: 19450233.
14. Masuzzo P, Hulstaert N, Huyck L, Ampe C, Van Troys M, Martens L. CellMissy: a tool for management, storage and analysis of cell migration data produced in wound healing-like assays. Bioinformatics. 2013;29(20):2661-3. doi: 10.1093/bioinformatics/btt437. PubMed PMID: PMC3789541.
15. Nyegaard S, Christensen B, Rasmussen JT. An optimized method for accurate quantification of cell migration using human small intestine cells. Metabolic Engineering Communications. 2016;3:76-83. doi: https://doi.org/10.1016/j.meteno.2016.03.002.
16. Goecks J, Nekrutenko A, Taylor J. Galaxy: a comprehensive approach for supporting accessible, reproducible, and transparent computational research in the life sciences. Genome Biology. 2010;11(8):R86-R. doi: 10.1186/gb-2010-11-8-r86. PubMed PMID: PMC2945788.
17. Fernandez-Gutierrez MM, Roosjen PPJ, Ultee E, Agelink M, Vervoort JJM, Keijser B, et al. Streptococcus salivarius MS-oral-D6 promotes gingival re-epithelialization in vitro through a secreted serine protease. Scientific Reports. 2017;7(1):11100. doi: 10.1038/s41598-017-11446-z.
18. R Development Core Team. R: A Language and Environment for Statistical Computing. Vienna, Austria: R Foundation for Statistical Computing; 2011.
19. Lindblad J, Bengtsson E, editors. A comparison of methods for estimation of intensity nonuniformities in 2D and 3D microscope images of fluorescence stained cells. Proceedings of the 12th Scandinavian Conference on Image Analysis (SCIA); 2001.
20. Otsu N. A Threshold Selection Method from Gray-Level Histograms. IEEE Transactions on Systems, Man, and Cybernetics. 1979;9(1):62-6. doi: 10.1109/TSMC.1979.4310076.
21. Vincent L, Soille P. Watersheds in digital spaces: an efficient algorithm based on immersion simulations. IEEE Transactions on Pattern Analysis and Machine Intelligence. 1991;13(6):583-98. doi: 10.1109/34.87344.
22. Zwietering MH, Jongenburger I, Rombouts FM, van 't Riet K. Modeling of the Bacterial Growth Curve. Applied and Environmental Microbiology. 1990;56(6):1875-81.
23. Ebner R, Derynck R. Epidermal growth factor and transforming growth factor-alpha: differential intracellular routing and processing of ligand-receptor complexes. Cell Regulation. 1991;2(8):599-612. PubMed PMID: PMC361851.
24. Huang C, Jacobson K, Schaller MD. MAP kinases and cell migration. Journal of Cell Science. 2004;117(20):4619-28. doi: 10.1242/jcs.01481.
25. Mebratu Y, Tesfaigzi Y. How ERK1/2 activation controls cell proliferation and cell death: Is subcellular localization the answer? Cell Cycle. 2009;8(8):1168-75. doi: 10.4161/cc.8.8.8147.
26. Bhattacharya R, Xu F, Dong G, Li S, Tian C, Ponugoti B, et al. Effect of bacteria on the wound healing behavior of oral epithelial cells. PLoS ONE. 2014;9(2). doi: 10.1371/journal.pone.0089475.
27. Laheij AM, de Soet JJ, Veerman EC, Bolscher JG, van Loveren C. The influence of oral bacteria on epithelial cell migration in vitro. Mediators of inflammation. 2013;2013:154532. doi: 10.1155/2013/154532. PubMed PMID: 24288439; PubMed Central PMCID: PMC3832977.
28. Ng MR, Besser A, Danuser G, Brugge JS. Substrate stiffness regulates cadherin-dependent collective migration through myosin-II contractility. The Journal of Cell Biology. 2012;199(3):545.
29. Zhang J-H, Chung TDY, Oldenburg KR. A Simple Statistical Parameter for Use in Evaluation and Validation of High Throughput Screening Assays. Journal of Biomolecular Screening. 1999;4(2):67-73. doi: 10.1177/108705719900400206.

30. Fernandez-Gutierrez, M, M; van Zessen, D, B; Baarlen, P, V; Kleerebezem, M; Stubbs, A, P (2018): Supporting data for "KREAP: An automated Galaxy Platform to Quantify Re-Epithelialization Kinetics" GigaScience Database. http://dx.doi.org/10.5524/100472

Figure legends

Figure 1. KREAP workflow. The virtual machine contains the KREAP toolbox and uses the graphical user interface (GUI) provided by Galaxy, including HTML reporting. The KREAP toolbox consists of Image Analysis and Data-Modelling tools. Logos indicate the use of specialized (open source) software or programming environments in different stages of the data processing. Red parallelograms indicate input and green parallelograms indicate output. Python was used to integrate the non-Galaxy applications into Galaxy tools.

Figure 2. KREAP Image Analysis tool graphical output example. Image segmentation output can be easily compared with the raw images. Automatic recognition of scratch boundaries enables the enumeration of nuclei inside and outside of the scratched area over time.

Figure 3. KREAP Data-Modelling tool output example. Re-epithelialization kinetics are described by the estimation of the λ, μ_m and A parameter values. The parameter values, simulation data and re-epithelialization curves per replicate are provided in an HTML report and can be downloaded through the available links.

Figure 4. Exemplary results obtained with the KREAP toolbox. (a) Enumeration of single cells infiltrating the scratched area over time. Ca9-22 cells were treated with TGFα (4 ng/ml), a solution containing p38 and MEK1/2 inhibitors (10 μM each) or left untreated. (b) Comparison of the repair rate (μ_m parameter) obtained with the different treatments. (c) Comparison of the maximum numbers of infiltrating cells (A parameter) obtained with the different treatments. Significant differences from the non-treated control were assessed by a one-way ANOVA using a Dunnett's test for multiple comparisons (n = 3; *, $P < 0.05$; **, $P < 0.01$; ***, $P < 0.001$).

Figure 5. Correlation between the parameter values originated with a multi-software approach and the KREAP toolbox. (a) Repair rate (μ_m parameter, cells minute^{-1}) and (b) Maximum number of cells (A parameter, cells). The correlation between the parameter values was evaluated by Pearson correlation analysis

(n = 215) and for both cases a positive and significant correlation was found ($P < 0.0001$).

Figure 6. Identification of adverse effects on re-epithelialization. Adverse effects on re-epithelialization are characterized by a low R^2 value as a result of extensive cell death after reaching the plateau of the growth curve.

Figure 1 Click here to access/download;Figure;Figure 1.pdf

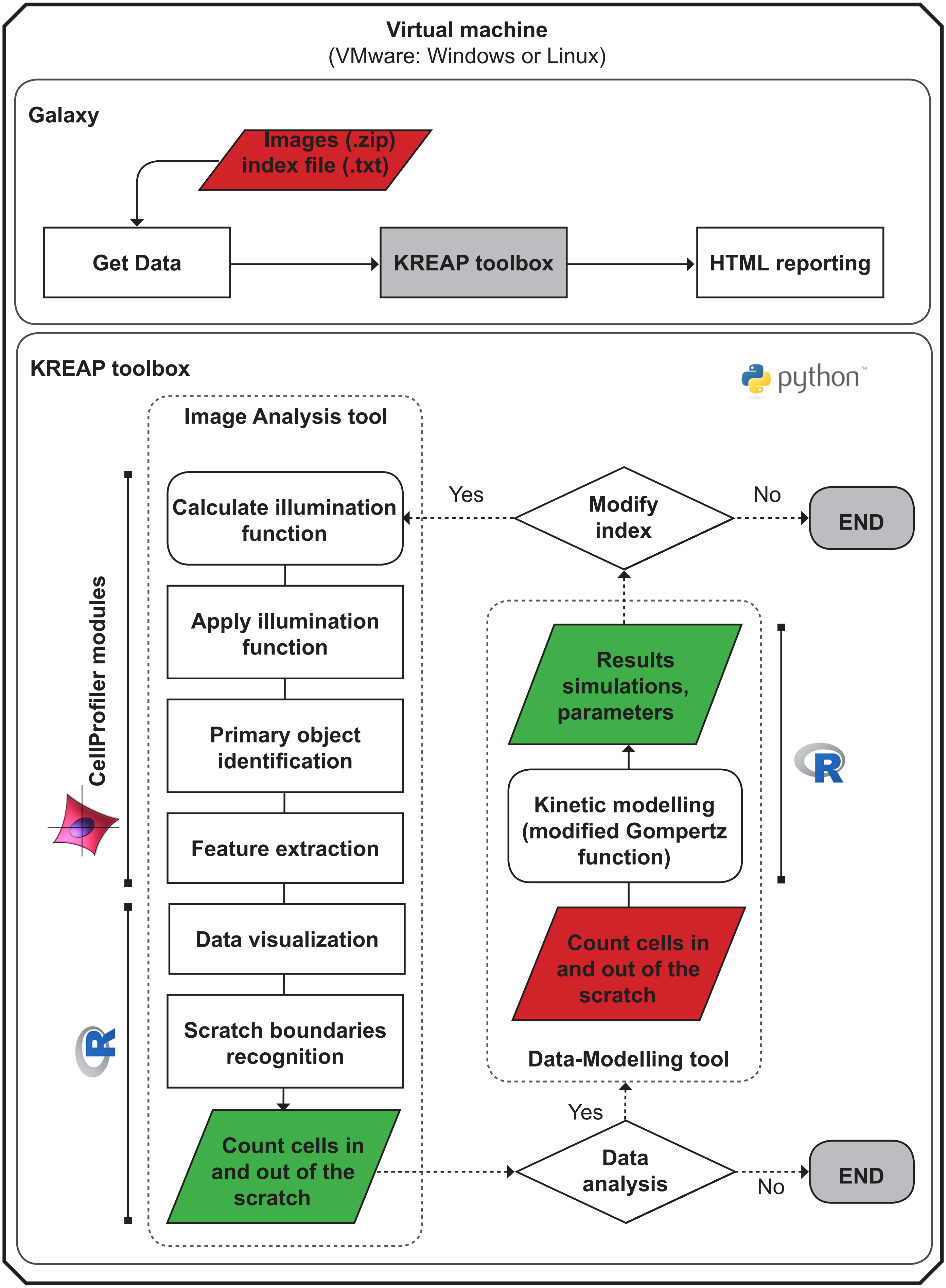

Figure 2

Click here to access/download;Figure;Figure 2.pdf

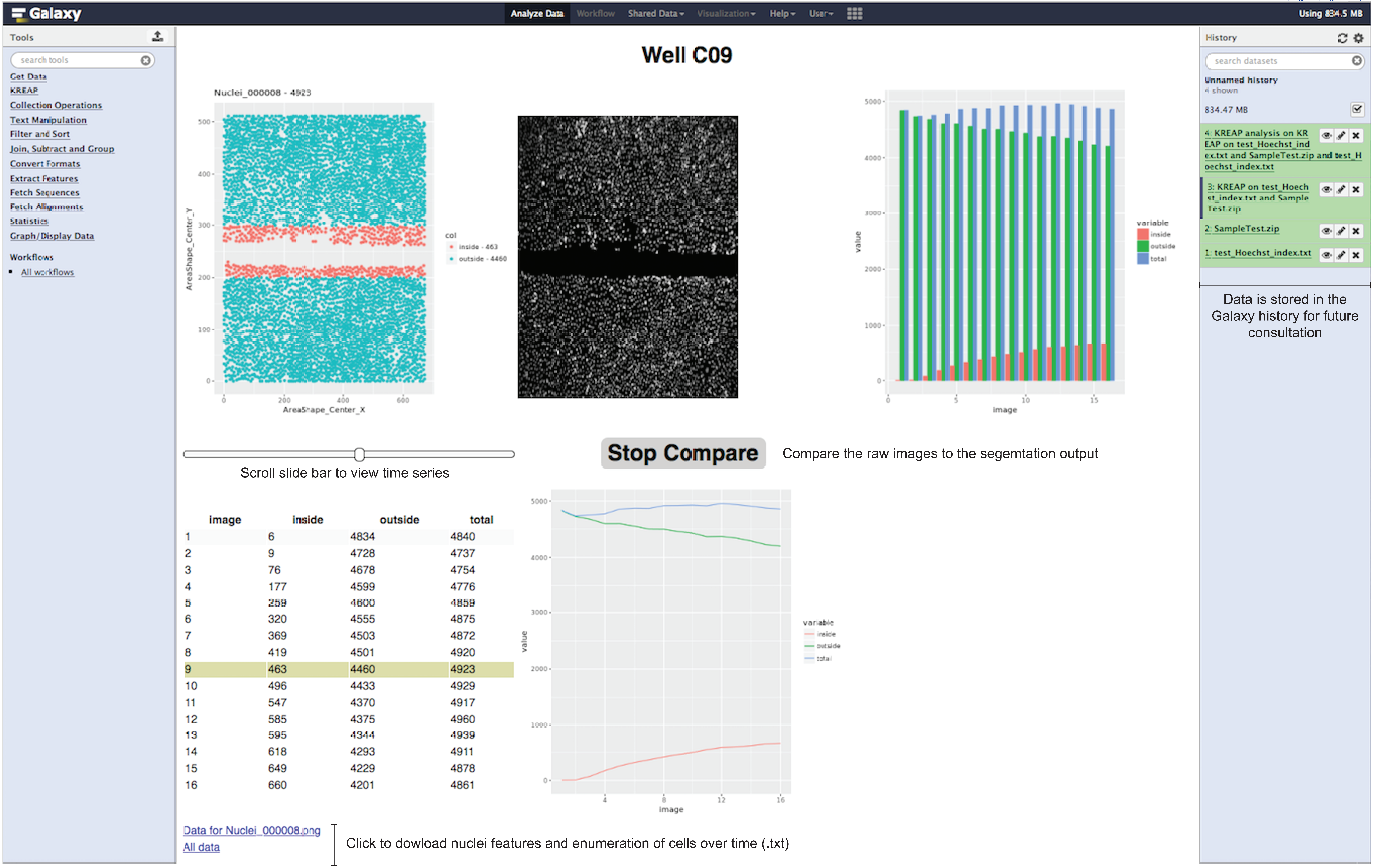

image	inside	outside	total
1	6	4834	4840
2	9	4728	4737
3	76	4678	4754
4	177	4599	4776
5	259	4600	4859
6	320	4555	4875
7	369	4503	4872
8	419	4501	4920
9	463	4460	4923
10	496	4433	4929
11	547	4370	4917
12	585	4375	4960
13	595	4344	4939
14	618	4293	4911
15	649	4229	4878
16	660	4201	4861

Figure 2

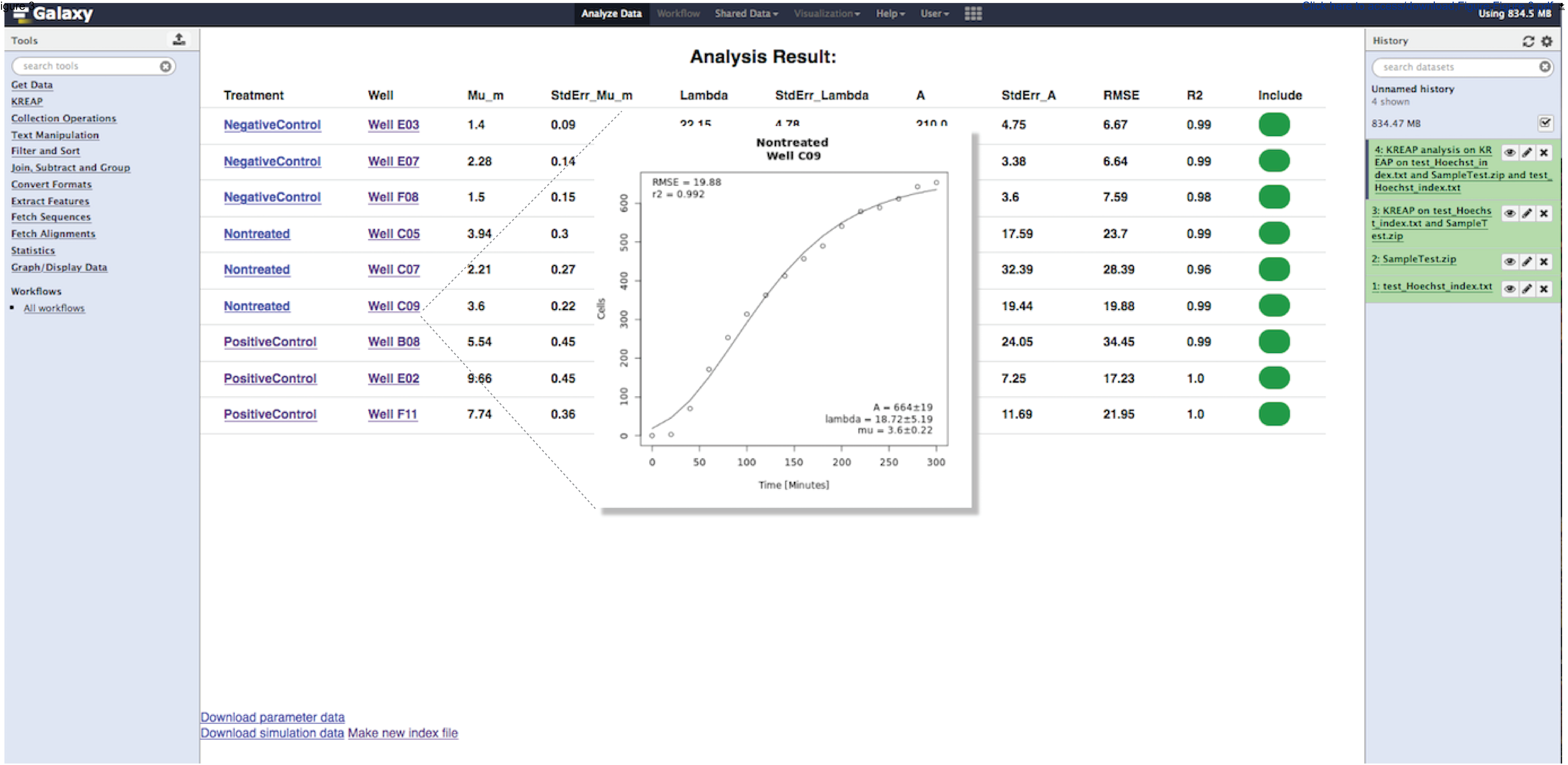
Galaxy
Analyze Data
Workflow
Shared Data
Visualization
Help
User
Using 834.5 MB
Tools
search tools
Get Data
KREAP
Collection Operations
Text Manipulation
Filter and Sort
Join, Subtract and Group
Convert Formats
Extract Features
Fetch Sequences
Fetch Alignments
Statistics
Graph/Display Data
Workflows
All workflows
Analysis Result:
Treatment Well Mu_m StdErr_Mu_m Lambda StdErr_Lambda A StdErr_A RMSE R2 Include
NegativeControl Well E03 1.4 0.09 4.75 6.67 0.99
NegativeControl Well E07 2.28 0.14 3.38 6.64 0.99
NegativeControl Well F08 1.5 0.15 3.6 7.59 0.98
Nontreated Well C05 3.94 0.3 17.59 23.7 0.99
Nontreated Well C07 2.21 0.27 32.39 28.39 0.96
Nontreated Well C09 3.6 0.22 19.44 19.88 0.99
PositiveControl Well B08 5.54 0.45 24.05 34.45 0.99
PositiveControl Well E02 9.66 0.45 7.25 17.23 1.0
PositiveControl Well F11 7.74 0.36 11.69 21.95 1.0
Nontreated
Well C09
RMSE = 19.88
r2 = 0.992
A = 664±19
lambda = 18.72±5.19
mu = 3.6±0.22
Cells
Time [Minutes]
Download parameter data
Download simulation data
Make new index file
History
search datasets
Unnamed history
4 shown
834.47 MB
4: KREAP analysis on KREAP on test_Hoechst_index.txt and SampleTest.zip and test_Hoechst_index.txt
3: KREAP on test_Hoechst_index.txt and SampleTest.zip
2: SampleTest.zip
1: test_Hoechst_index.txt

Figure 4

Click here to access/download;Figure;Figure 4.pdf

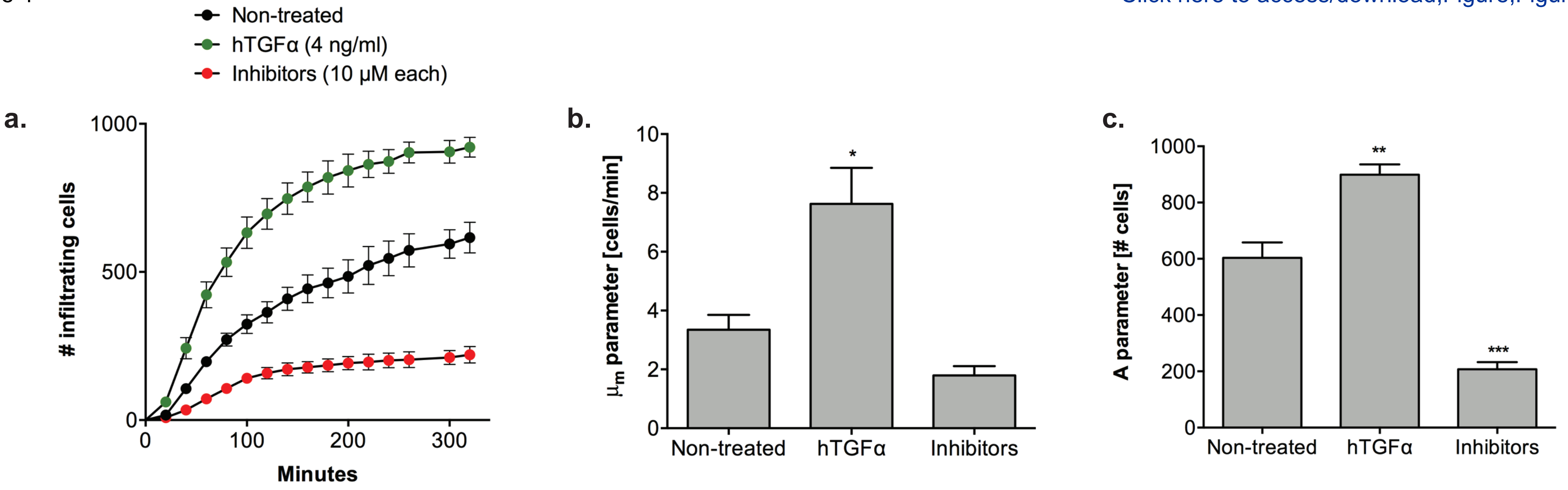

Figure 5

Click here to access/download;Figure;Figure 5.pdf

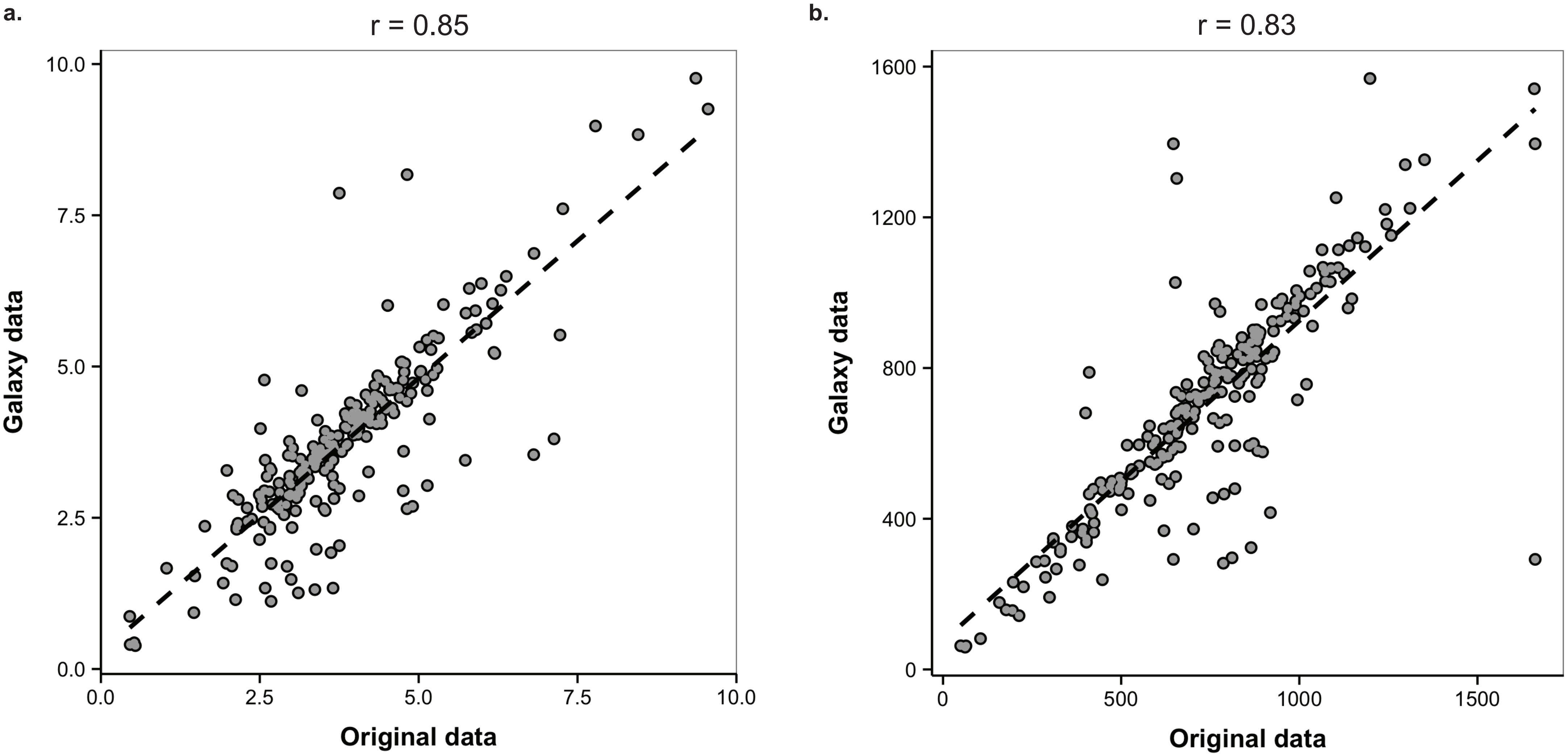

Figure 6

Click here to access/download;Figure;Figure 6.pdf

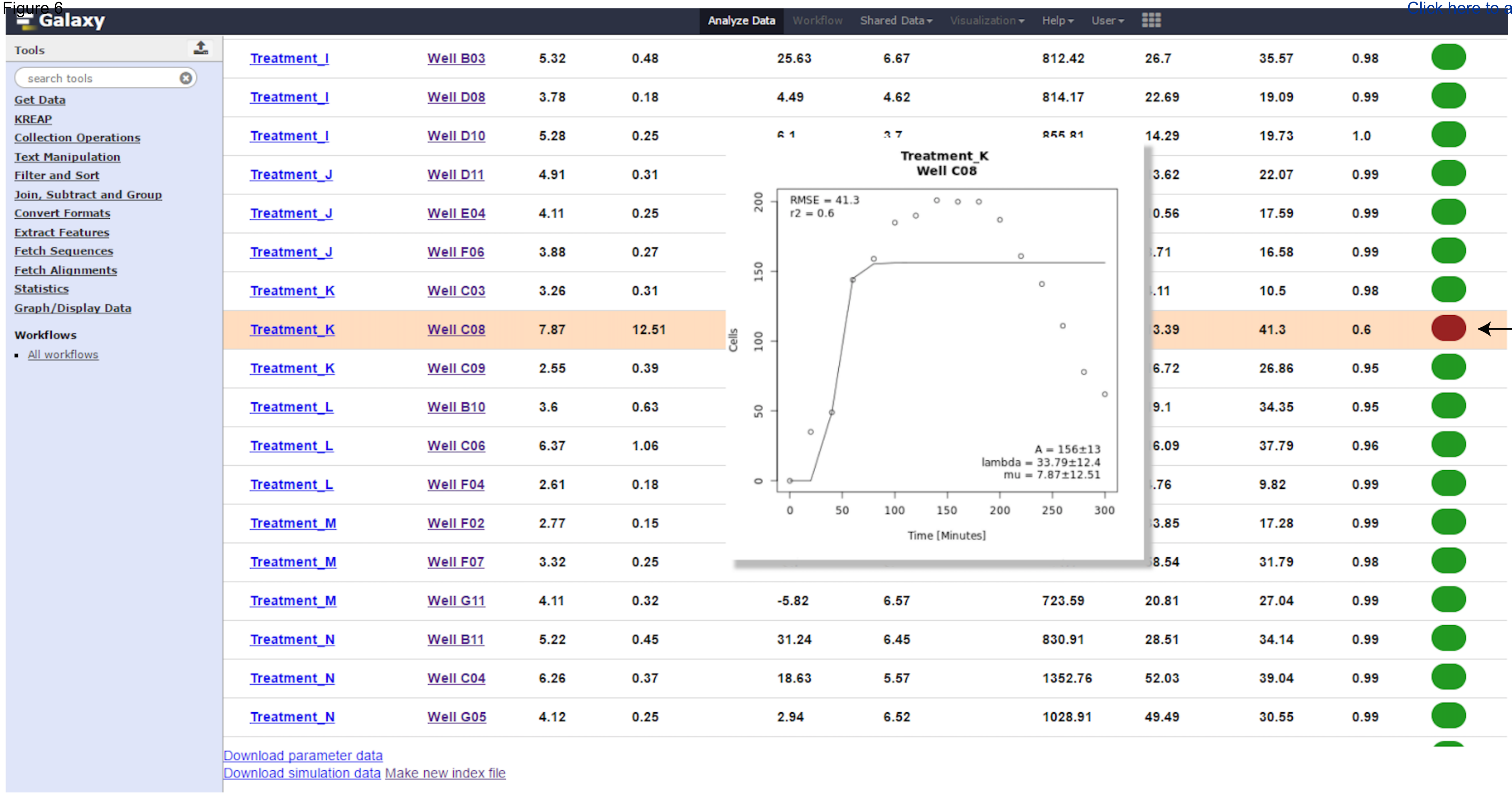

R²lower than 0.9 are flagged by the KREAP toolbox

Additional Manuscript

Click here to access/download
Supplementary Material
Additional Manuscript.pdf

Click here to access/download
Supplementary Material
Supplementary Information.pdf
