# Supplementary material for: KREAP: an automated Galaxy platform to quantify in vitro re-epithelialization kinetics
Source: Gigascience. 2018 Jun 28;7(7):giy078. doi: 10.1093/gigascience/giy078 (PMC6048990; doi:10.1093/gigascience/giy078)
Supplement: Reviewer_1_Original_Submission_Attachment_giga-science.pdf [file giy078_reviewer_1_original_submission_attachment_giga-science.pdf]

Review report of the Technical Note “KREAP: An automated Galaxy Platform to Quantify Re-Epithelialization Kinetics” by Fernandez-Gutierrez and Van Zessen et al.

In their Technical Note “KREAP: An automated Galaxy Platform to Quantify Re-Epithelialization Kinetics”, Fernandez-Gutierrez and Van Zessen et al. report an open web-based Galaxy service, termed KREAP, for high-content quantifying of wound healing assays. The authors use a method that was recently introduced by the group (ref. 16), that is based on (1) CellProfiler nuclei segmentation, (2) Re-implementation of modules that previously required a proprietary software. The main contribution of this Technical Note is making their workflow publically available and easy to use via an open-source web-based service that should enable high content studies, which is stated to be “of particular interest” to GigaScience (https://academic.oup.com/gigascience/pages/technical_note#Criteria). However, several critical concerns are preventing me from supporting the manuscript for publication, at least in its current form, as detailed below.

<u>Crucial concerns</u>:

KREAP is limited to live imaging of nuclei stained with a fluorescent markers. Vast majority of wound healing experiments are based on phase-contrast or other imaging modalities that do not require nuclei staining with the reasoning of simplifying the experiments and less inference with the cell model. The only reason I can see to require nuclei staining in this type of experiments is to enable single cell analysis. However, the parameters that KREAP extracts are not exploiting the single nuclei information – in principle, λ, μ_m, and A could be calculated using area measurements rather than cell counts. As I detail below, the authors have not convinced me that (1) KREAP measures that are based on nuclei staining are more informative in describing wound healing kinetics compared to other available measures designed for phase-contrast imaging; and (2) KREAP implementation is robust and can be easily applied to a variety of data-sets; These are crucial in arguing the usability and importance of KREAP.

- Previous methods for temporal quantification, and high content data management. It is true that "data analysis of scratch assays has been traditionally limited to the quantification of percentage of wound closure during the course of the experiment [3], neglecting the kinetic information inherent of the re-epithelialization process (e.g. repair rate) and hampering the possibility of single cell migration analysis.” (#60-63). However, in the last decade, quite a few measures and tools were proposed to quantify temporal (or spatio-temporal) dynamics in wound healing experiments and to manage high-content data. A few examples (there are many more):
    - Vitorino and Meyer (2008), “Modular control of endothelial sheet migration”, calculated multiple kinetic measures and via screening demonstrated that the different measures are modularly controlled by different molecular drivers.

- Defort et al. (2012), “Automated velocity mapping of migrating cell populations (AVeMap)”, provided a publically-available tool that extracted multiple kinetic measures, validated on 67 movies from the screen by Simpson et al. (ref 9 in the manuscript), and verified that they do not lose information in relation to single cell measurements.
- Zaritsky et al. (2017), “Diverse roles of guanine nucleotide exchange factors in regulating collective cell migration”, provided spatiotemporal measurements and demonstrated how to use them for high-content screening.
- Masuzzo et al. (2013), “CellMissy: a tool for management, storage and analysis of cell migration data produced in wound healing-like assays”, provided a open-source tool for wound healing data management.

The authors have entirely missed all this body of work. It is essential to acknowledge the relevant literature and discuss the benefits and limitations of the experimental setting and measures proposed.

- Is there an advantage using single nuclei data over phase-contrast? Can the authors demonstrate that additional information is encoded in the single nuclei information that cannot be quantified by segmenting cellular-vs-background at the pixel-level? I would argue that λ, μ_m, and A can be calculated without the single nuclei information directly from the phase-contrast segmentation. I find this concern critical in arguing for a more complex experimental setting. Perhaps the information encoded in the nuclei shape, that can be measured with this approach can provide this added information? This can be tested on the data from ref. 16.
- Relevance of the extracted parameters. What information / insight is gained by using λ, μ_m, A? I would expect μ_m to be highly correlative to the wound healing rate. It is not clear to me what biological insight can be gained from A (the authors mention that λ is only important for accurate estimation of μ_m). Can the authors correlate the different parameters to one another and to the overall wound healing rate? Can it be demonstrated what A has a biological interpretation that is independent of μ_m? Can you find modular experimental conditions for these two measures?
- Applicability to diverse data-sets. To demonstrate robustness of the method and broad usability, I would like to see the authors demonstrate their software usability on an additional dataset, preferably collected by another lab. If no such dataset is easily available than doesn’t it question the need for such a software?

<u>Essential corrections</u>:

- Data availability. It was not clear to me whether the full dataset presented in the authors’ previous manuscript (ref #16) is available. In ref #16 the authors state that "The datasets generated during the current study are available from the corresponding author on

reasonable request." In the current Technical Note, I could not find a clear statement whether the full dataset is publically available (and citable), and where can it be found.

- The project webpage https://erasmusmc-bioinformatics.github.io/KREAP/ is not linked to a GitHub page. I could not find it even with direct search in GitHub homepage for KREAP. Also, could not find the source code.
- Methods are not described in a clear and sufficient manner.
  - The description of the parameters λ, μ_m and A assumes that readers are familiar with the Gompertz function. Brief explicit description of the Gompertz function will make this clear.
  - #139-141: "Automatic identification of the scratch boundaries was programmed in R by finding the largest distance between cells and then expanding up and down looking for smaller gaps to avoid incorrect determination of the scratch boundaries." These steps are very vague, please be more explicit and explain systematically how this step is performed (I did not find a sufficient explanation in ref. 16).
  - From briefly reading ref. 16 I did not find direct and clear description regarding how the screening scores were calculated from the multiple replicates per condition and negative/positive controls.
- I find it hard to understand how to interpret the "performance value" measure, μ_m*A. Why is it a meaningful measure? What does it teach us about the wound healing kinetics more than μ_m alone?

<u>Minor suggestions</u>:

- Availability and Requirements:
  - "Operating system: Unix-based Operating Systems" – I think this is supposed to imply on what OS can KREAP be executed on, which is not limited to Unix.
  - License: replace "free" with the formal license, see here, https://opensource.org/licenses/category
- Giga Science focus on high-content datasets and analyses. Since the analyzed dataset includes 60 movies (each with 16 time points), it can be marginally considered as "large-scale" in the field. This would be another (not critical) argument for including another dataset in this Technical Note.
- The dataset used in this Technical Note is not well descried. Please describe the experiment, controls, and statistics. I am aware that these were (at least partially) described in ref. 16, but (I think that) these reporting standards are expected in GigaScience. Personally, I could not easily figure out how the controls were used together with the replicates to score hits in ref. 16.

- I do not see the point in Fig. 4 (#91-94). This is a trivial validation that the implementation replicates what was measured in ref. 16, especially since it is a paper by the same group. I would recommend excluding it or moving it to be a Supporting figure.
- I also think that Figure 5 would be better suited as a Supporting figure. I am in favor of focusing on the main aspects and leaving less important details for SI.
- The points discussed in “crucial concerns” should be discussed.

Disclaimer: In reviewing the manuscript I did not test the Galaxy web-service (due to time limitations). From looking at the figures it seem reasonably user friendly and include all the information reported in the Technical Note.
