# Supplementary material for: KREAP: an automated Galaxy platform to quantify in vitro re-epithelialization kinetics
Source: Gigascience. 2018 Jun 28;7(7):giy078. doi: 10.1093/gigascience/giy078 (PMC6048990; doi:10.1093/gigascience/giy078)
Supplement: Reviewer_1_Revision_1_Attachment_giga-science.pdf [file giy078_reviewer_1_revision_1_attachment_giga-science.pdf]

Review report of the Technical Note “KREAP: An automated Galaxy Platform to Quantify Re-Epithelialization Kinetics” by Fernandez-Gutierrez and Van Zessen et al.

I appreciate the efforts of the authors which improved the manuscript considerably. KREAP web-based implementation provides an automated and easy-to-use infrastructure for high-content analysis of *in-vitro* wound healing experiments, data organization and visualization. I am now also convinced that the approach of counting the cells’ nuclei could be more informative in cases were cells infiltrate the free space in a more individual manner. I am still concerned about several points.

Major issues:

- **Limitation to live imaging of nuclei stained with a fluorescent markers.** Following the authors’ rebuttal letter and revised MS (# 74-76) it is now clear to me that the major benefit of counting single nuclei is for the case of cells with weaker cell-cell adhesion that detach from the bulk and migrate individually to the open space. This is a pain to quantify that many biologists suffer from and will be appreciated. The authors use a cell line that seem to maintain cohesiveness. This point could be convincingly illustrated by applying KREAP to cells that are more individual (the authors mention the FHs74 cell line), as an additional example. This is not a necessary experiment, but could emphasize this advantage of the methodology.
    - I advice the authors to focus on this point and down-play the arguments on bacteria or viability, which are quite esoteric and not relevant for the vast majority of potential users.
    - I think there was a misunderstanding regarding my point on advantages over phase contrast microscopy. I was not eluding to counting / detecting single cells in phase contrast images, which is terribly hard. Rather I was proposing to follow the advancing monolayer edge over time, which is the standard measure for scratch experiments, or even using it to calculate the same parameters λ, μ_m, A. The authors can demonstrate the superiority of their approach over extracting the exact same parameters from the monolayer advancement rate with the experiment I proposed above (using less cohesive cells).
    - Demonstrating KREAP robustness. I think this was another point of confusion in communication. I am fully aware of the prevalence of scratch essays. However, the 3,575 pubmed results for "scratch AND assay" do not provide information on whether nuclei staining exists (and was used for the quantification). I was arguing that a second dataset could be retrieved from another lab’s previous publication to verify the applicability to diverse data-sets. And that the lack of access to such data (through contacting a senior author of such a paper) imply that researchers are not staining their cells when performing scratch assays. I still think that this could be an important (but not necessary) addition to this manuscript.

- **Measurements**.
  - λ, μ_m, A are not standard measures to quantify scratch assays and must be accompanied with their biological meaning. Users should have insight on how to interpret these measures. Please see if I got it right and revise the text accordingly.
    - The definition of λ, μ_m, and A is now clear but their biological interpretation is not clear to me. I do not find the following authors' respond helpful: "As we showed in ref. 19 (Fig. 2), the A parameter correlates to the maximum surface area (i.e. pixels) covered with cells (r = 0.68, P = 1.36 x 10-6). Hence, these parameters describe different aspects of the re-epithelialization kinetics and should therefore, not be correlated to each other.". This simply provides an example that A is correlated with the wound healing rate and nothing on different biological interpretation of these measures.
    - I am not convinced that these parameters encode the "initial cellular responses to a bioactive substance" (see below) and the "subsequent activation/inhibition of cell proliferation" (much of the area covered in these assays is usually explained by cell spreading rather then proliferation).
    - The authors' response that "in Fig. 6a we provide an example in which addition of a particular treatment initially induced rapid cell migration (represented by a high m value), but during the course of the experiment, migration of cells into the scratched area ceased, resulting in a low A parameter value." is not a convincing demonstration as in the text this effect relates to experimental failure that led to cell death.
    - I would argue that μ_m could be seen as a more accurate measure than the standard "wound healing rate" (how fast the free area is covered by cells). Because it corrects for the lag and stationary phases.
    - For large scratches, I expect A to be highly correlated with μ_m (at least until the cell density is dramatically reduced).
    - For a given condition I expect the stationary phase to start earlier for smaller scratches, as the density increases in the "wound" area. Does A encode any additional biological-relevant parameter beyond μ_m and the wound size?
    - Can the authors explain whether λ has a biological interpretation in the setting of a scratch assay? Could an example be provided were λ is systematically altered without affecting the other parameters? I am concerned that λ might encode a technical parameter of the experimental setting (perhaps "batch effect").

        - The authors should (1) better explain the biological interpretation of the parameters they extract; (2) provide data to support these explanations, this includes correlations between the different parameters and/or different experimental conditions that alter one parameter independently of the other (modularity). If the authors intend to argue that μ_m, and A encode "subsequent activation/inhibition of cell proliferation", this has to be shown by verifying how much of the increased area covered by the cells is explained by spreading and how much by proliferation. This is a critical point in my review.
    - My request to discuss pros and cons compared to alternative approaches was in the context of comparing the extracted measurements and not in the context of open/commercial software. I still believe it is necessary and important to put these measures in context with other available solutions in a discussion highlighting the cons and pros of the measures that extract via KREAP. One new manuscript that just came out and could be of interest to discuss (if you find it appropriate) is https://www.biorxiv.org/content/early/2018/01/15/248104.

Other corrections and suggestions:

- **Data availability**. GigaDB was built for the purpose of making GigaScience publication data publically available (https://gigascience.biomedcentral.com/articles/10.1186/2047-217X-1-11)
- Line #78: Scratch is an assay for migration, quantifying cell death and migration together is esoteric, and KREAP (as currently implement) donot provide the means to quantify it.
- Line #146: "the difference in intensity is used to separate merged objects into individual ones" – how? Please provide more details (it sounds like a watershed algorithm?)
- Line #157-159: Automatic identification of the scratch boundaries
    - The description "finds the largest empty area by measuring the frequency of cells" is not sufficiently detailed. How exactly is this area determined?
    - More importantly, this implementation assumes a very specific setting of the scratch assay – a vertical cell free area in the middle of the field of view. This limitation (e.g., the software is not suitable for circular scratches; images of horizontal scratches must be rotated; would it work for scratches were the other side is not in the field of view?) must be mentioned in the text and in the software user guides!
- Line #172: the parameters λ, μ_m, and A are mentioned but defined only later at line #79. I would suggest to present them first at the location they are defined, otherwise it could be cryptic and confusing.

- Lines #190-192: "Importantly, we have shown that the A parameter is correlated to the maximum surface area in pixels covered by cells". Why is this important? Isn't it trivial? I suggest to exclude this text.
- Line #193: Would be insightful to explicitly provide the physical/biological interpretation of the 'performance value' (μ_m*A), which is not obvious to me – is it a standard measure in a different assay (then please provide a reference or at least an explanation)? Also, this measure is not used throughout the manuscript – why mention it, then?
- Line #199:
- Line #203-205: I do not see the point in this.
- Line #213-217: Please report the fold change in addition to the p-value.
- Line #219- : Please describe the sub-dataset. How large is it? What was the criteria to select experiments from the full dataset?
- Line #245-247: "detrimental effects" are not defined in the main text (should be independent of the figure legend).
- Line 257-259: Please provide guidelines on how to determine if a given experiment is faulty (dead cells) or a strong effect caused by a perturbation? Especially when the software is not providing an image of the cells. Is there a way to decide based on the curves?
- Legend of Figure 4: no reference to panel (b)
- Figure 4a: The "analysis image" (right) does not fit in size to the other two and can not be compared
- Figure 1: The "modify index" is not explained in the figure legend nor the main text. Is it just switching to the next well?
- Figure 2: it is hard to compare the input and output due to the resizing – can this be fixed?

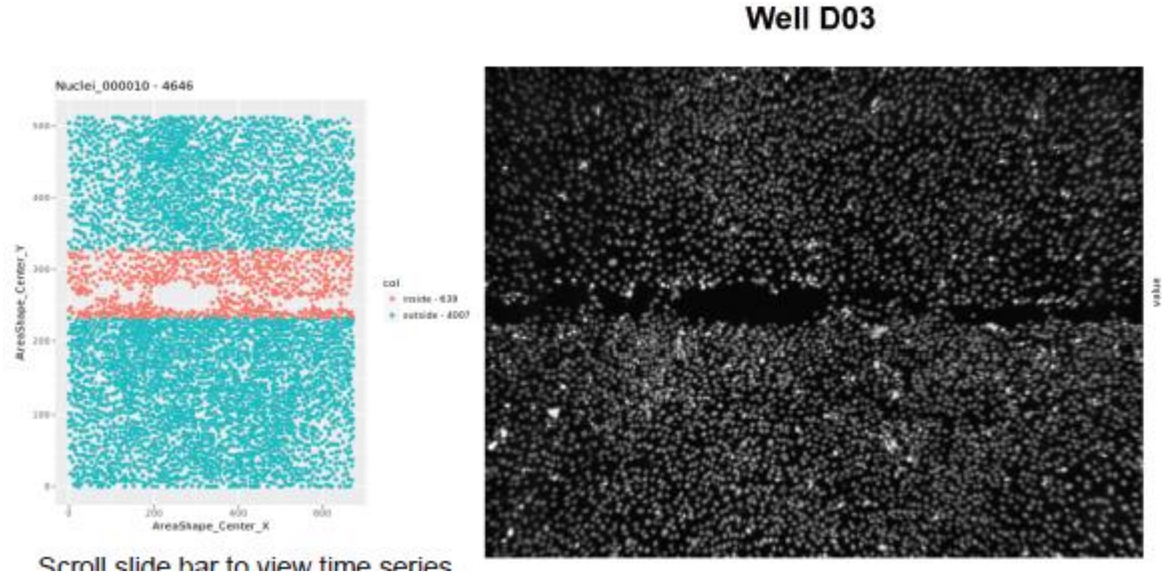

- Figure 3 legend: make the first sentence bold to fit the other legends.

- The link to the github repository is broken (I could not get to the point of entering the user name and password)

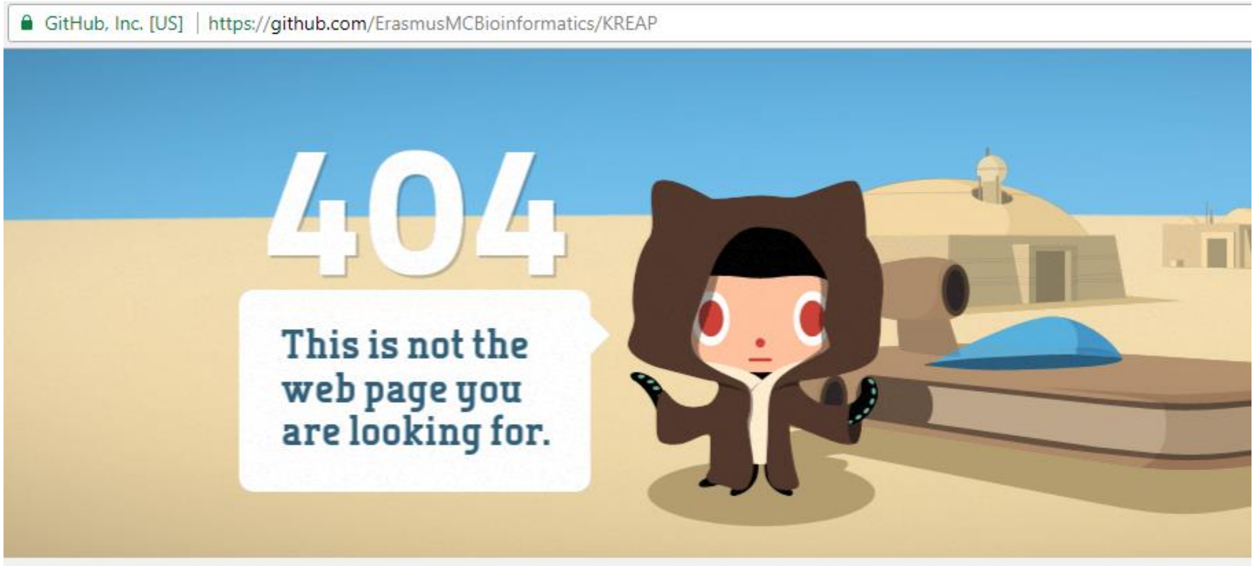

- Methods should include detailed description of the algorithmic pipeline. Some of the main text could be transferred (and elaborated) in the Methods.

Sincerely,

Assaf Zaritsky, UTSW
