# Supplementary material for: KREAP: an automated Galaxy platform to quantify in vitro re-epithelialization kinetics
Source: Gigascience. 2018 Jun 28;7(7):giy078. doi: 10.1093/gigascience/giy078 (PMC6048990; doi:10.1093/gigascience/giy078)
Supplement: Additional Files [file giy078_supplemental_files.zip › Supplementary Information.pdf]

Supplementary Information

KREAP: An automated Galaxy Platform to Quantify in vitro Re-Epithelialization Kinetics

Marcela M. Fernandez-Gutierrez[1,2$], David B.H. van Zessen[2$], Peter van Baarlen[2], Michiel Kleerebezem[1,2], Andrew P. Stubbs[3*]

[1]TI Food and Nutrition, Nieuwe Kanaal 9-A, 6709 PA, Wageningen, The Netherlands.

[2]Host-Microbe Interactomics, Animal Sciences Group, Wageningen University & Research, De Elst 1, 6708 WD, Wageningen, The Netherlands.

[3]Department of Bioinformatics, Erasmus University Medical Centre, Wytemaweg 80, 3015 CN, Rotterdam, The Netherlands.

$ - Both authors contributed equally

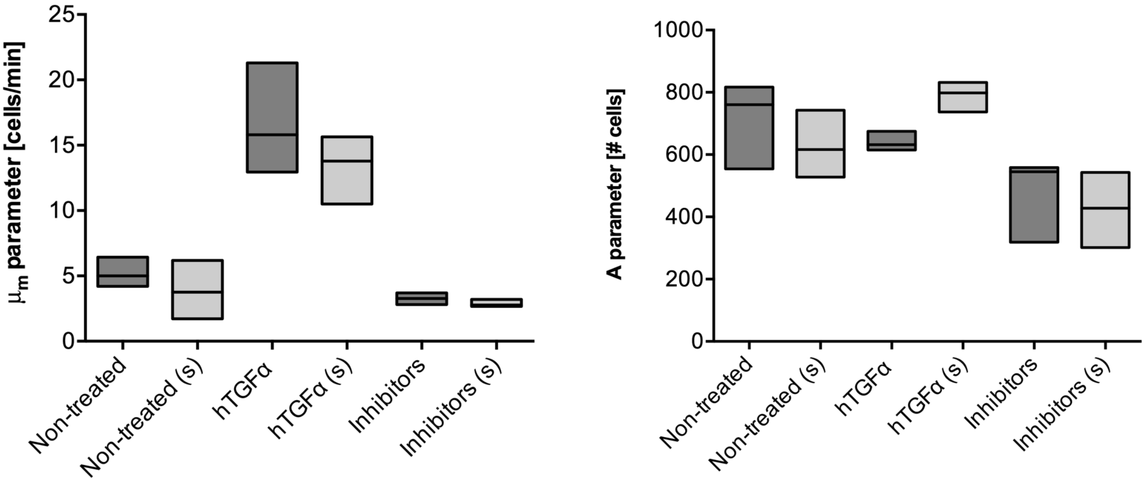

Figure S1. Comparison of the re-epithelialization kinetics obtained with fluorescently stained and unstained cells. The nucleus and cytoplasm of gingival epithelial cells (Ca9-22 cell line) were stained for 20 minutes with a solution containing 2 μg/ml Hoechst 33342 and 2 μM Cell Tracker™ Red CMTPX or left unstained. The parameter values describing re-epithelialization kinetics (μ_m and A) were calculated for the unstained cells using phase-contrast images. Nuclei counts were used for the enumeration of fluorescently stained cells, which are labelled with (s) in the bar plots. A Kruskal-Wallis test with Dunn's correction for multiple comparison did not find significant differences between the stained and unstained cells (n = 3).

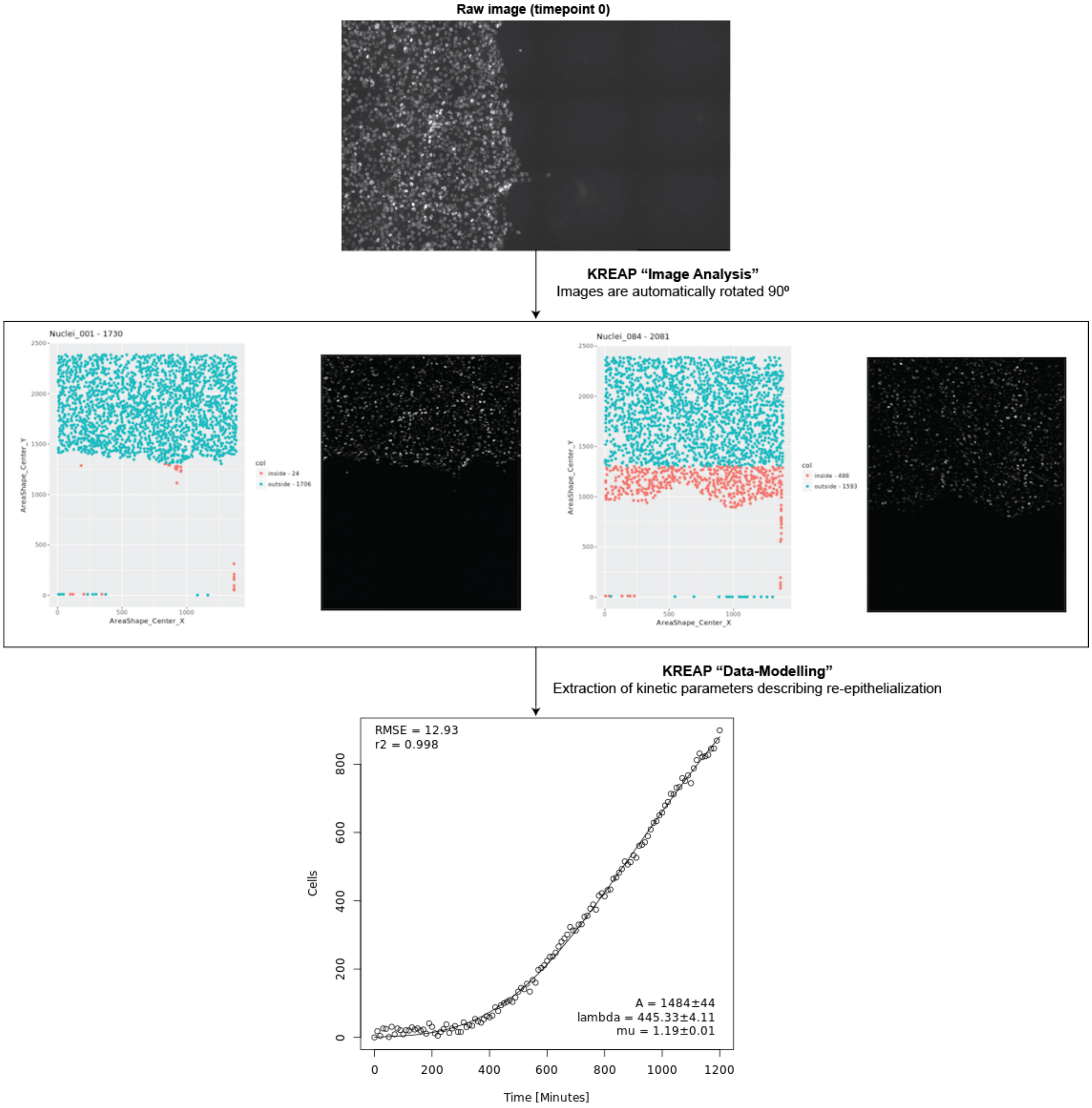

Figure S2. Validation of the KREAP toolbox with a published image-series. Image-series were obtained from a published study [1]. Briefly, MCF10A cells expressing H2B-mCherry as a nuclear marker were generated by infection with the pBabe-H2B-mCherry retroviral vector. Wound-healing assays were carried out on polyacrylamide gel substrates prepared on 12-well-glass-bottomed dishes. Fluorescent images were acquired with a 20x objective lens every 10 minutes using an inverted motorized microscope with a custom-built incubation chamber maintained at 37° and 5% CO_2 (Ti-E, Nikon) [1]. As indicated in the index file, the images were automatically rotated 90° by the KREAP Image Analysis tool and processed with the analysis pipeline to identify cells inside and outside the scratched area over time. The resulting re-epithelialization curve was successfully modelled by the KREAP Data-Modelling tool, extracting three biological relevant parameters describing re-epithelialization kinetics (λ, μ_m, and A). Notably, the calculated lambda value is not negligible (as compared to our results with the Ca9-22 cell line), indicating that in this cell line there is a delay in the initiation of re-epithelialization after the introduction of the scratch.

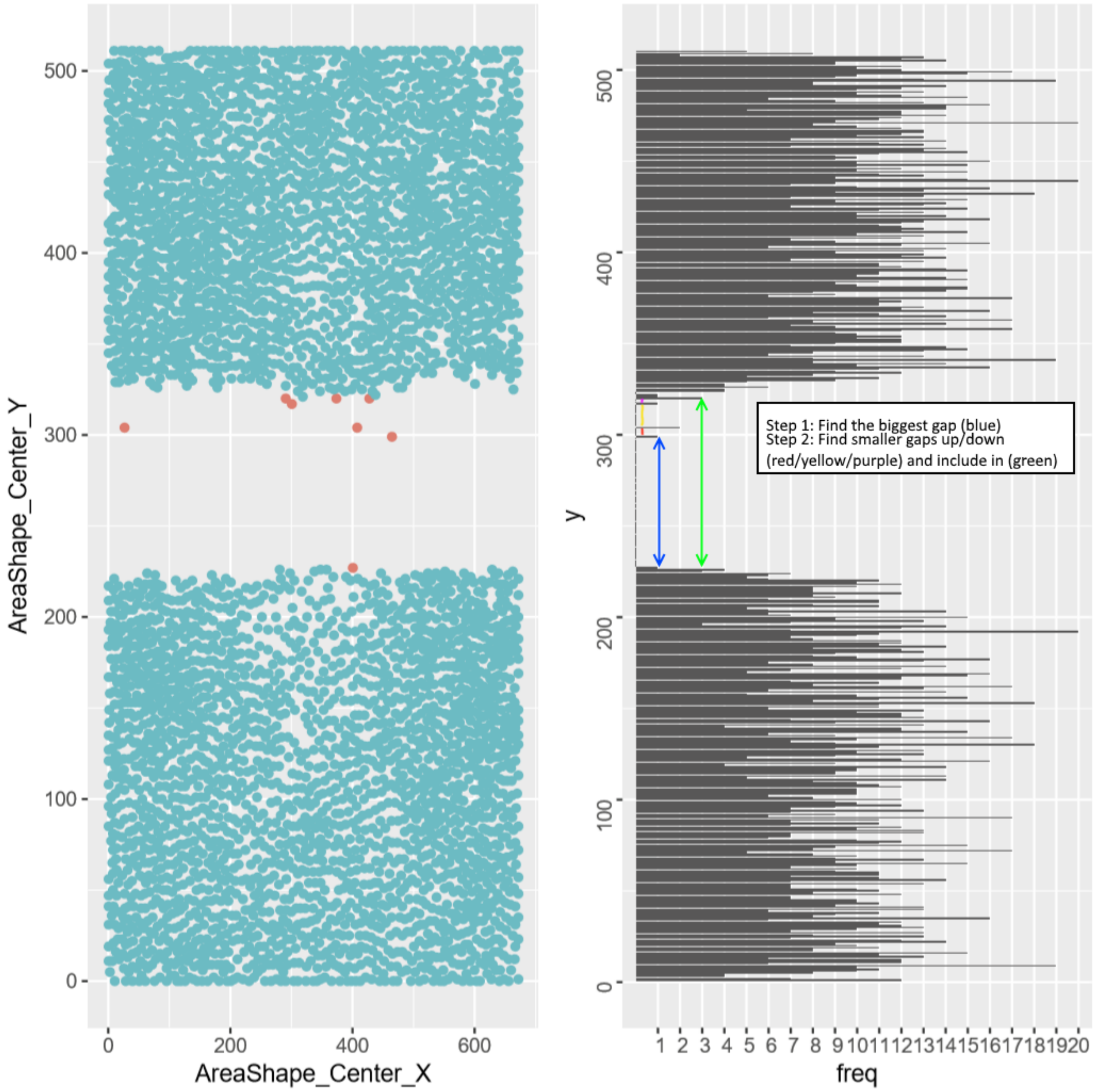

Figure S3. Schematic view of the automatic recognition of the scratch boundaries performed by KREAP. KREAP detects the largest cell-free area in each well by measuring the cell frequency on the Y axis at the beginning of the assay. To avoid underestimation of the scratch size that could result if single cells are left within the scratched area, the algorithm searches for smaller gaps up- and downwards of the largest cell-free area and adds them up resulting in the final identification of the scratch boundaries.

Reference

1. Ng MR, Besser A, Danuser G, Brugge JS. Substrate stiffness regulates cadherin-dependent collective migration through myosin-II contractility. The Journal of Cell Biology. 2012;199(3):545.
