# Supplementary material for: KREAP: an automated Galaxy platform to quantify in vitro re-epithelialization kinetics
Source: Gigascience. 2018 Jun 28;7(7):giy078. doi: 10.1093/gigascience/giy078 (PMC6048990; doi:10.1093/gigascience/giy078)
Supplement: Response_to_Reviewer_Comments_Original_Submission.pdf [file giy078_response_to_reviewer_comments_original_submission.pdf]

Author's Response To Reviewer Comments

Close

Dear Scott Edmunds,

Below the point-point response to the referees on the technical note GIGA-D-17-00209 entitled "KREAP: An automated Galaxy Platform to Quantify Re-Epithelialization Kinetics". Furthermore, we thank the reviewers for their valuable time and useful contribution.

Answers to Reviewer #1

Critical concerns:
1. KREAP is limited to live imaging of nuclei stained with fluorescent markers. Vast majority of wound healing experiments are based on phase-contrast or other imaging modalities that do not require nuclei staining with the reasoning of simplifying the experiments and less inference with the cell model. The only reason I can see to require nuclei staining in this type of experiments is to enable single cell analysis. However, the parameters λ, μm and A could be calculated using the area measurements rather than cell counts.

Author response: Although image analysis could be performed with high quality phase-contrast images (see Additional file 1), we highly recommend the use of fluorescent labels for the reasons that we mention below. In summary, the use of fluorescent dyes increases the contrast between the cell or cellular organelle of interest and the background, thus improving accuracy of counting (or tracking) cells and/or organelles. Additionally, the use of specific fluorescent dyes allows the determination of viable, dead and apoptotic cell numbers as well as the number of proliferating cells.

(1) Nowadays, the use fluorescent microscopy in combination with live-compatible and fixable dyes is becoming a standard technique in most laboratories. For example, Vitorino and Meyer (2018), "Modular control of endothelial sheet migration", used 10 g/ml wheat germ agglutinin conjugated to AlexaFluor594 for 10 minutes before scraping the cells. This dye is used to label cell membranes of living cells and therefore, get a clear delimitation of the cell boundaries for automated image analysis.
(2) The use of live-compatible dyes involves a simple and fast staining step in which you add the labelling solution into each well and wait from 10-30 minutes. After the incubation time, the scratches can be introduced into the stained cell monolayers. Importantly, we have tested the influence of Hoechst 33342 and other live-compatible dyes such as CellTracker™ Red CMTPX in comparison with non-labelled cells and we showed that the fluorescent dyes do not interfere with the normal physiology of the cells (see Additional File 1).
(3) When evaluating the effect of certain bacteria on re-epithelialization, it is highly recommended to use florescent dyes because many bacteria tend to co-aggregate, interfering with the accuracy of the image analysis if carried out using bright-field images (see Fig. 4a, lines 201-205). Hence, applying fluorescent dyes to label specific cell organelles, such as the nuclei or the cell membrane, removes such artefacts and improves the accuracy of the results.
(4) Fluorescent dyes in scratch assays are not only used for single cell tracking, but also to

detect apoptotic cells or live/dead cells. For example, Nyegaard et al. (2016), "An optimized method for accurate quantification of cell migration using human small intestine cells", used SYTO-24 and propidium iodide to determine live versus dead cell counts (also see lines 76-78 for more references). This provides the opportunity to accurately determine total cell numbers and numbers of live and dead cells within the same microscopy view in one go.
(5) KREAP image analysis was designed to identify single cells and thereby, quantify the number of cells infiltrating into the scratched area over time. Fluorescent stains contribute greatly to accurately identify single cells, and nuclei are excellent markers for cells that do not contain segmented or multi-lobed nuclei such as epithelial cells.

In principle, the modified Gompertz function used by KREAP to model re-epithelialization kinetics could be used with area coverage measurements. However, we argue that single cell counting in combination with an automatic determination of the scratch boundaries is a more accurate method to quantify re-epithelialization kinetics. As shown by Nyegaard et al. (2016), "An optimized method for accurate quantification of cell migration using human small intestine cells (Fig. 2)", poorly adherent cell lines (e.g. FHs-74 int cells) detach and migrate in an uneven pattern making final wound closure determination more challenging.

2. The authors have not convinced me that, KREAP measures that are based on nuclei staining are more informative in describing wound healing kinetics compared to other available measures designed for phase-contrast imaging

Author response: The image analysis performed by KREAP can in principle be also conducted with bright-field images when using high-quality, high contrast (see Additional File 1). However, the use of fluorescent dyes is highly recommended (see point #1 above) to improve the accuracy of the results obtained by removing possible artefacts present in the raw bright-field images and provide additional possibilities such as determining the ratios of viable/dead cells in the same field / sample.

3. The authors have not convinced me that, KREAP implementation is robust and can be easily applied to a variety of data-sets. These are crucial in arguing the usability and importance of KREAP.

Author response: In the revised version of the manuscript we included a section with anticipated results (lines 199-218) with an exemplary dataset. In addition, the KREAP toolbox was used to re-analysed a sub-dataset (total of 215 image series) from a previous study in which we used a fundamentally different analysis pipeline that involved the manual determination of the scratch boundaries. A Pearson correlation analysis confirmed the association between the results obtained with KREAP and those obtained in the previous study (lines 219-244, Fig. 5).

4. In the past decade, quite a few measures and tools were proposed to quantify temporal (or spatio-temporal) dynamics in wound healing experiments and to manage high-content data. The authors entirely missed all this body of work. It is essential to acknowledge the relevant literature and discuss the benefits and limitations of the experimental setting and measures proposed.

Author response: In the revised version of the manuscript, we expanded the background information on current tools available for high-throughput image analysis and in specific, high-throughput scratch assays and provide an overview of their features (lines 61-76). We also recognize that other studies such as the ones carried out by Vitorino and Meyer (2008) and Zaritsky et al. (2017) have provided spatio-temporal measurements of high-throughput scratch assays. However, these studies used commercial software or in-house developed analysis pipelines that are not freely available for other scientists.

We want to stress here that the added value of the KREAP procedure is that: (1) it provides an open-source, high-throughput analysis platform; (2) it provides a fully automated data analysis workflow in Galaxy that provides a user friendly interface, including comprehensive graphical outputs, experimental history and data storage; and (3) it is designed to recognize single cells and applies a nonlinear least squares regression to invert the modified Gompertz function and thereby, estimate the values of three independent biologically relevant parameters that describe different phases of the re-epithelialization process (see point #6).

5. Is there an advantage using single nuclei data over phase-contrast? Can the authors demonstrate that additional information is encoded in the single nuclei information that cannot be quantified by segmenting cellular-vs-background at a pixel-level? I would argue that the λ, μm and A can be calculated without the single nuclei information directly from the phase-contrast segmentation. I find this concern critical in arguing a more complex experimental setting. Perhaps the information encoded in the nuclei shape, that can be measure with this approach can provide this added information? This can be tested on the data from ref. 16.

Author response: In principle, automated cell recognition can be executed on high-quality, phase-contrast images or fluorescently stained cells. However, the accuracy of the image segmentation is higher when using fluorescently labelled cell organelles, and it offers additional possibilities to explore the same sample, for instance by quantifying ratios of live and dead cells. As explained in point #1, the addition of live-compatible fluorescent dyes does not require a complex experimental setup and in most laboratories is considered a standard technique. Furthermore, the enumeration of single cells over time results in a sigmodial curve that can be modelled by inverting the modified Gompertz function (see point #6).

6. Relevance of the extracted parameters. What information / insight is gained by using λ, μm and A? I would expect μm to be highly correlative to the wound healing rate. It is not clear to me what biological insight can be gained from A (the authors mention that the is only important for accurate estimation of μm). Can the authors correlate the different parameters to one another and to the overall wound healing rate? Can it be demonstrated that A has a biological interpretation that is independent of μm? Can you find modular experimental conditions for these two measures?

Author response: The modified Gompertz function was originally developed to model bacterial growth curves and is described by Zwietering et. al. (1990), "Modeling of the Bacterial Growth Curve". These characteristic sigmodial growth curves consist of a lag phase, an exponential phase, and a stationary phase. As we described in ref. 19 of the revised manuscript, when we plot the number of epithelial cells that migrate into the scratch area over time, we consistently

obtained sigmodial growth curves. As explained in lines 178-192 of the revised manuscript, λ represents the lag time or the amount of time (in minutes) before the cells start migrating into the scratched area. The μm parameter is an estimation of the repair rate (cells/min-1), and the A parameter is the calculation of the maximum number of cells that entered into the scratched area. As we showed in ref. 19 (Fig. 2), the A parameter correlates to the maximum surface area (i.e. pixels) covered with cells (r = 0.68, P = 1.36 x 10-6). Hence, these parameters describe different aspects of the re-epithelialization kinetics and should therefore, not be correlated to each other. These are biologically relevant parameters reflect the initial cellular responses to a bioactive substance and subsequent activation/inhibition of cell proliferation. These are insights that are relevant to clinical research, infectious diseases, cancer and toxicology, to name a few.

We are not completely sure what the reviewer means with "finding modular experimental conditions", but in Fig. 6a we provide an example in which addition of a particular treatment initially induced rapid cell migration (represented by a high m value), but during the course of the experiment, migration of cells into the scratched area ceased, resulting in a low A parameter value.

7. Applicability to diverse data-sets. To demonstrate robustness of the method and broad usability, I would like to see the authors demonstrate their software usability on an additional dataset, preferably collected by another lab. If no such dataset is easily available, then doesn't it question the need for such software?

Author response: A search on PubMed using the key words "scratch AND assay" resulted in a total of 3,575 search items, which exemplifies the usability of such a tool for the scientific community. However, due to the size of image datasets, publication reporting on scratch assays do not provide access to the raw images that could be used to test the usability of KREAP. Nonetheless the performance of KREAP was validated with a total of 215 image series obtained in a previous study (see point #3).

Essential corrections:

8. Data availability. It was not clear to me whether the full dataset presented in the author's previous manuscript (ref. 16) is available. In ref. 16 the authors state that "The datasets generated during the current study are available from the corresponding author on reasonable request". In the current Technical Note, I could not find a clear statement whether the full dataset is publicly available (and citable), and where it can be found.

Author response: The dataset in ref. 16 (ref. 19 of the revised manuscript) is available upon request, which is related to the large size of these datasets (approximately 15 GB). This is probably the reason why other publications reporting on scratch assays do not provide access to raw data or are limited to one image series. Analogously, in the present manuscript we provide exemplary image dataset as stated in lines 317-319: "The dataset supporting the results of this article is available at the KREAP homepage (Kinetic Re-Epithelialization Analysis Pipeline)". If GigaScience is willing to support storage and availability of the complete datasets as supplementary data, we are happy to upload the files.

9. The project webpage https://erasmusmc-bioinformatics.github.io/KREAP/ is not linked to a GitHub page. I could not find even with direct search in GitHub homepage for KREAP. Also, could not find the source code.

Author response: The authors regret the confusion. The supporting data is available in the KREAP project homepage. Also, the code will be made available when the paper is accepted and until then the authors will provide the reviewers and anonymous account and password to login to access the code for KREAP. The source code is available for reviewers using the reviewer login details supplied by the authors at: https://github.com/ErasmusMC-Bioinformatics/KREAP (username: erasmusmc-review; password: erasmusmcreview123). This login will be replaced by open access if the paper is accepted for publication.

10. Methods are not described in clear and sufficient manner.

a. The description of the λ, μm and A assumes that readers are familiar with the Gompertz function. Brief explicit description of the Gompertz function will make it more clear.

Author response: We expanded the explanation of the modified Gompertz function in lines 168-192 to have a more clear overview of each of the parameters estimated by the model as well as their biological explanation.

b. # 139-141: "Automatic identification of the scratch boundaries was programmed in R by finding the largest distance between cells and then expanding up and down looking for smaller gaps to avoid incorrect determination of the scratch boundaries". These steps are very vague, please be more explicit and explain systematically how this step is performed (I did not find sufficient explanation in ref. 16).

Author response: In the revised version of the manuscript we change this statement to make it less vague and more explicit (lines 157-159).

c. From briefly reading ref. 16 I did not find direct and clear description regarding how the screening scores were calculated from the multiple replicates per condition and negative/positive controls.

Author response: Ref. 16 (ref. 19 of the revised manuscript) reports on much more than the initial screening scores and describes how negative and positive scores were identified. However, in the current manuscript we provide a section with anticipated results and their interpretation (Fig. 4, lines 199-218) as well as the methods used to execute the experiment (lines 281-307).

11. I find hard to understand how to interpret the "performance value" measure, μm*A. Why is it a meaningful measure? What does it teach us about the wound healing kinetics more than m alone?

Author response: The μm and A represent independent biological relevant parameters. The combination of them in a single value (μm*A) provides a simple overall assessment of re-

epithelialization performance under a certain treatment. See also answer to point #6 and lines 192-198.

Minor suggestions:

12. Availability and requirements:

d. "Operating system": Unix-based Operating Systems" - I think this is supposed to imply on what OS can KREAP be executed on, which is not limited to Unix.

Author response: The authors regret the confusion and clarified this point in the revised version of the manuscript by stating that the source code/algorithms were developed in Linux, whereas the VM can be executed using Microsoft Windows, Linux or Mac OS X (lines 311-312).

e. License: replace "free" with formal license, see here, https://opensource.org/license/category

Author response: We thank the reviewer for his remark. KREAP is freely available under the MIT open-source license as stated in line 314.

13. GigaScience focus on high-content datasets and analyses. Since the analysed dataset includes 60 movies (each with 16 timepoints), it can be marginally considered as "large scale" in the field. This would be another (not critical) argument for including another dataset in this Technical Note.

Author response: We regret we were not sufficiently clear in the previous version of the manuscript. As explained in the revised manuscript, the 60 image-series served as an example to indicate the computational time needed (lines 238-244) to obtain the quantitative results using KREAP. For validation of the KREAP performance, we re-analysed a total of 215 image-series, thereby, including additional datasets (also see point #3) from a previous study.

14. The dataset used in this Technical Note is not well described. Please describe the experiment, controls, and statistics. I am aware that these were (at least partially) described in ref. 16, but (I think that) these reporting standards are expected in GigaScience. Personally, I could not easily figure out how the controls were used together with the replicates to score hits in ref. 16.

Author response: In our opinion, the present manuscript does not intend to repeat the analyses as they were presented in ref. 16 (ref. 19 of the revised manuscript), but to provide the tools needed to quantify re-epithelialization kinetics. In the current manuscript we provide a section with anticipated results and their interpretation (Fig. 4, lines 199-218) as well as the methods used to execute the experiment (lines 289-307). We would like to emphasise here that the KREAP tool provides result feedback to enable recognition of potential problem samples or image-series that require the user's attention.

15. I do not see the point of Fig. 4 (#91-94). This is a trivial validation that the implementation replicates what was measured in ref. 16, especially since it is a paper by the same group. I

would recommend excluding it or moving it to be a Supporting figure.

Author response: The workflows use fundamentally different procedures to determine the wound boundaries (lines 220-223). The original workflow used in ref. 16 (ref. 19 of the revised manuscript) depends on the manual definition of a rectangular wound for each well at the beginning of the measurements. KREAP automatically defines the wound boundaries by actual recognition of cells at the boundaries which is more accurate and less subjective than manual handling. Therefore, we consider Fig. 5 in the revised manuscript highly relevant for the verification of the accuracy of the KREAP tool and propose to maintain the figure in the revised version of the manuscript.

16. I also think that Figure 5 would be better suited as Supporting figure. I am in favour of focusing on the main aspects and leaving less important details for SI.

Author response: In our opinion, it is very important to point users of KREAP to potential issues in data analysis and interpretation that they might not be familiar with. As an additional service to users, we implemented automated flagging of such issues to ensure that these are not missed by the user. Figure 5 (Fig. 6 in the revised manuscript) illustrates examples of the troubleshooting output, which we prefer to maintain as a figure in the main manuscript.

Answers to Reviewer #2

1. I would make a discretionary revision recommendation that instead of shipping the .vmdk virtual drive it would be beneficial to utilize the .ova (Open Virtual Appliance) format for simpler, more standard distribution. A docker version would probably be well-received, too, as it's rapidly becoming the standard way to ship services like this.

Author response: We have added a converted OVA to download. We agree with the reviewer that a docker would probably be well received. We will create a docker if there is sufficient demand from the research community to access KREAP using this format.

2. It would be nice for these tools to ultimately be added to the Galaxy Tool Shed for non-VM distribution, though it looks like the dependencies might be cumbersome to deal with and I understand choices made here.

Author response: The dependencies were indeed the reason why we did not add the tools to the Tool Shed. We considered adding the tools to the Tool Shed and let the server maintainer install the dependencies, but it would have been a very "non Galaxy" thing to do.

3. It'd be more convenient to add an additional tool in Galaxy that can consume a collection to generate this file, or perhaps modify the initial image analysis tool itself to support collections, in addition to the pre-bundled zip format.

Author response: We looked at collections, but we decided against using them because we would not be able to add the metadata (i.e. time, pixel_block_size, etc) KREAP needs without making the tool interface much more complicated. The ZIP approach allowed us to limit this

complexity to an Excel sheet and organizing and zipping a directory, things most scientists are already familiar with.

Close
