# Supplementary material for: KREAP: an automated Galaxy platform to quantify in vitro re-epithelialization kinetics
Source: Gigascience. 2018 Jun 28;7(7):giy078. doi: 10.1093/gigascience/giy078 (PMC6048990; doi:10.1093/gigascience/giy078)
Supplement: Response_to_Reviewer_Comments_Revision_1.pdf [file giy078_response_to_reviewer_comments_revision_1.pdf]

# Author's Response To Reviewer Comments

Close

Dear Scott Edmunds,

Below we present our point-to-point response to the referee on the technical note GIGA-D-17-00209 entitled "KREAP: An automated Galaxy Platform to Quantify in vitro Re-Epithelialization Kinetics". Once again, we thank the reviewers for their time and critical input.

Major issues:
1. Limitation to live imaging of nuclei stained with fluorescent markers.
1.1. I advise the authors to focus on this point (that the major benefit of counting single nuclei is for the case of cells with weaker cell-cell adhesion that detach from the bulk and migrate individually) and down-play the arguments on bacteria or viability, which are quite esoteric and not relevant for the vast majority of potential users.

Author response: As recommended by the reviewer, the argument on bacteria or cell viability is now removed from the revised manuscript. However, we believe that there is increased interest on exploring the influence of bacteria on wound repair and therefore, the application of the method described in our manuscript (using fluorescent markers) could benefit not only the users using cell lines with weak cell-cell adhesions, but also users with an interest on performing high-throughput screenings with viable bacteria or other substances that may aggregate. To illustrate this point, we provide four examples of studies in which scratch assays were used to test the influence of bacteria on wound repair:
i. Mohammedsaeed, W., Cruickshank, S., McBain, A. J. & O'Neill, C. A. Lactobacillus rhamnosus GG Lysate Increases Re-Epithelialization of Keratinocyte Scratch Assays by Promoting Migration. Scientific Reports 5, 16147 (2015).
ii. Fernandez-Gutierrez, M.M.; Roosjen, P.P.J.; Ultee, E.; Agelink, M.; Vervoort, J.J.M.; Keijser, B.; Wells, J.M.; Kleerebezem, M. Streptococcus salivarius MS-oral-D6 promotes gingival re-epithelialization in vitro through a secreted protein. Scientific Reports 7(1):11100, (2017).
iii. Laheij, A. M., de Soet, J. J., Veerman, E. C., Bolscher, J. G. & van Loveren, C. The influence of oral bacteria on epithelial cell migration in vitro. Mediators of inflammation 2013, 154532, doi:10.1155/2013/154532 (2013).
iv. Bhattacharya, R. et al. Effect of bacteria on the wound healing behavior of oral epithelial cells. PLoS ONE 9, doi:10.1371/journal.pone.0089475 (2014).

1.2 I was not eluding to counting/detecting single cells in phase contrast images, which is terribly hard. Rather, I was proposing to follow the advancing monolayer edge over time, which is the standard measure for scratch experiments, or even using it to calculate the same parameters λ, µm and A. The authors can demonstrate the superiority of their approach over extracting the exact same parameters from the monolayer advancement rate with the experiment I proposed above (using less cohesive cells).

Author response: This point was addressed in the previous rebuttal where we explained the reasons why we think that the use of live-compatible fluorescent labels in combination with single-cell identification can provide a more accurate determination of wound healing kinetics than the classical

measurement of following the monolayer edges over time. We agree with the reviewer that testing the performance of KREAP with a less cohesive cell line would have strengthen our manuscript but unfortunately, we do not have such a cell line in-house.

1.3 Demonstrating KREAP robustness. (…) I was arguing that a second data-sets could be retrieved from another lab's previous publication to verify the applicability to diverse datasets. And that the lack of access to such data (through contacting a senior author of such a paper) imply that researchers are not staining their cells when performing scratch assays. I still think that this could be an important (but not necessary) addition to this manuscript.

Author response: In the revised manuscript, we proved the usability of our pipeline with an external image-series (lines 276-285, Supplementary Fig. S2). We demonstrate that the KREAP toolbox can be used to process images with a vertical scratch provided that you indicate in the index file the degrees needed to rotate the images to obtain a horizontal scratch. Furthermore, we were also able to validate the performance of our segmentation pipeline with a different cell line (MCF10A) expressing H2B-mCherry as a nuclear marker. Finally, we could validate the performance of the automatic identification of the scratch boundaries even when only one side of the scratch is visible obtaining excellent fits of the modified Gompertz model. The image-series were provided by Mei Rosa Ng, the main author of the publication entitled: "Substrate stiffness regulates cadherin-dependent collective migration through myosin-II contractibility" published on 2012 in the Journal of Cell Biology.

2 Measurements.
2.1 The definition of λ, µm and A is now clear, but their biological interpretation is not clear to me. I do not find the following authors' response helpful: "As we showed in ref. 19 (Fig. 2), the A parameter correlates with the maximum surface area (i.e. pixels) covered with cells (r = 0.68, P = 1.36 x 10^-6). Hence these parameters describe different aspects of the re-epithelialization kinetics and should therefore, not be correlated to each other". This simply provides an example that A is correlated with the wound healing rate and nothing on different biological interpretation of these measures.

Author response: We apologize if we were not clear enough in our previous response. What we meant was that the λ, µm and A parameters describe different phases of the growth curve and therefore, we do not see how correlating the parameters to one another will provide more biological insight (it may actually confuse the interpretation; see also our answer under point 2.3). The µm parameter is an indicator of the wound healing rate, whereas the A parameter gives an indication of the 'status' of the wound (i.e. wound closure. To this end, the A parameter is correlated to the classical measurement of scratch assays that employ monolayer advancement (see point 1.2) which enables the calculation of the maximum wound closure achieved under a particular treatment after certain period of time. To test this, we normalized the A parameter values to that of the non-treated control and performed a Spearman correlation (n = 42) with the values obtained from calculating the relative wound closure as follows: 1 − [(# pixels in the scratch at time x)/(# pixels in the scratch at time 0) * 100] (also expressed relative to the non-treated control). The results showed a highly significant positive correlation between the two measurements (r = 0.68, P = 1.36 x 10^-6), providing evidence that the A parameter provides a 'snapshot' of the status of wound closure. This point was also clarified in the revised version of the manuscript in lines 193-205.

2.2 I am not convinced that these parameters encode the "initial cellular responses to a bioactive substance" (see below) and the "subsequent activation/inhibition of cell proliferation" (much of the

area covered in theses assays is usually explained by cell spreading rather than proliferation)

Author response: We agree with the reviewer that the area covered in a scratch assay can be explained by several processes including cell spreading, but area coverage is also influenced by enhancement of proliferation by the treatment. Depending on the agent, the treatment may accelerate proliferation (e.g. treatment with TGF-alpha or another growth factor) or inhibit proliferation (e.g. treatment with pathogenic Porphyromonas gingivalis or chemical inhibitors of proliferation). Since it is not always possible to exclude either spreading or proliferation a priori (and this may depend on the cell line used), we feel we should keep both explanatory mechanisms included.

2.3 The authors' response that in Fig. 6a we provide an example in which addition of a particular treatment initially induced rapid cell migration (represented by the high μm value), but during the course of the experiment, migration of cells into the scratched area ceased, resulting in a low A parameter value" is not demonstration as in the text this effect relates to experimental failure that led to cell death.

Author response: We would like to clarify that the values obtained with the treatment in Fig. 6a are not due to experimental failure. On the contrary, this was a biologically relevant result providing evidence that this particular bacterial preparation causes an initial stimulatory response in the cells which was reflected by a high migration rate (μm parameter). Over time, cell migration ceased, leading to an unresolved wound and therefore, a low A value. This is an example in which the μm and A parameters are negatively correlated. Hence, a high μm value does not necessary imply a high A value.

2.4 I would argue that the μm could be seen as a more accurate measure than the standard "wound healing rate" (how fast the free area is covered by cells) because it corrects for the lag and stationary phase. For large scratches, I expect A to be highly correlated with μm (at least until the cell density is dramatically reduced). For a given condition, I expect the stationary phase to start earlier for smaller scratches, as the density increases in the "wound" area. Does A encode any additional biological-relevant parameter beyond μm and the wound size?

Author response: As explained before in points 2.1 and 2.3, the A parameter provides a snapshot of the status of the wound and therefore is a separate measurement from the μm parameter. Application of the modified Gompertz model requires standardization of the wound size to minimize its variation (see Ref. 17 of the revised manuscript for more information on this). We included this remark in our revised manuscript on lines # 116-123. The wound size could be optimized using dedicated scratching tools such as the HTScratcher (Peira, BE) or defined cell-free gap inserts (ibidi, DE), among others. This eliminates variation in the wound size as a possible confounding factor.

2.5 Can the authors explain whether λ has a biological interpretation in the setting of a scratch assay? Could an example be provided were λ is systematically altered without affecting the other parameters? I am concerned that λ might encode a technical parameter of the experimental setting (perhaps "batch effect").

Author response: As explained in our manuscript (lines # 196-201) and in our previous study (Ref. 17 of the revised manuscript) the λ parameter (expressed in minutes) was consistently characterized by small to negative values, indicating that the migration process was initiated before the imaging acquisition started. Therefore, we concluded that in the cell lines tested, the λ parameter has a limited

biological meaning, but its calculation is essential to obtain an accurate approximation of the µm and A parameters.

2.6 The authors should (1) better explain the biological interpretation of the parameters they extract; (2) provide data to support these explanations, this includes correlations between the different parameters and/or different experimental conditions that alter one parameter independently of the other (modularity). If the authors intend to argue that the µm and A encode" subsequent activation/inhibition of cell proliferation", this has to be shown by verifying how much of the increased area covered by the cells is explained by spreading and how much by proliferation. This is a critical point in my review.

Author response: (1) We hope that we provided the reviewer with a clearer explanation of the biological interpretation of the parameters we extract in points 2.1, 2.4, and 2.5 of this rebuttal. (2) Please refer to point 2.3 for an example on modularity. We regret the misunderstanding in the previous rebuttal, but we do not intend to argue that the µm and A parameters encode for subsequent activation/inhibition of cell proliferation, rather we interpret these parameters as useful to describe re-epithelialization kinetics, which encompasses the processes of cell migration (cell spreading) and/or proliferation.

2.7 My request to discuss pros and cons compared to alternative approaches was in the context of comparing the extracted measurements and not in the context of open/commercial software. I still believe it is necessary and important to put these measures in context with other available solutions in a discussion highlighting the cons and pros of the measures that extract via KREAP.

Author response: As mentioned in our manuscript (lines # 58-68) there are different solutions for quantification of wound-healing assays. However, KREAP is specifically designed for high-throughput screenings and has the advantage over the other solutions that it is an open-source platform in Galaxy that provides quantitative re-epithelialization measurements using a novel modelling approach based on the quantification of single-cell infiltration.

Other corrections and suggestions:
1. Data availability. GigaDB was built for the purpose of making GigaScience publication data publicly available.

Author response: As we outlined before, we will address this point once our manuscript is accepted for publication. At the moment, our data is available in our homepage and in the Github repository.

2. Line # 78: Scratch is an assay for migration, quantifying cell death and migration together is esoteric and KREAP (as currently implement) do not provide the means to quantify it.

Author response: We agree with the reviewer and decided to remove that statement from the revised manuscript.

3. Line # 146: "the difference in intensity is used to separate merged objects into individual ones" – how? Please provide more details (it sounds like a watershed algorithm?)

Author response: As indicated by the reviewer, merged objects are separated by a watershed

algorithm. This was also specified in the revised version of the manuscript (lines # 155-156).

4. Line # 157 -159: Automatic identification of the scratch boundaries. The description "finds the largest empty area by measuring the frequency of cells" is not sufficiently detailed. How exactly is the area determined? More importantly, this implementation assumes a very specific setting of the scratch assay – a vertical cell free area in the middle of the field of view. This limitation (e.g., the software is not suitable for circular scratches; images of horizontal scratches must be rotated; would it work for scratches were the other side is not in the field of view?) must be mentioned in the text and in the software user guides!

Author response: We apologize to the reviewer for not providing a clear explanation. Here we include a diagram (also added as Supplementary Fig. S3 and explained in the revised manuscript lines 167-173) to make this point clear.

The software first searches for the largest cell-free area (main scratch), indicated by the blue arrow. Then, it searches for smaller gaps up and down of the main scratch (represented in red, yellow, and purple). These smaller gaps are then added up to the main scratch resulting in the identification of the scratch boundaries (represented with the green arrow).

In the revised version of KREAP, we also address the second point of the reviewer: we provide an option to rotate (clockwise) images directly in the index file by indicating how many degrees would be required to obtain a horizontal cell-free area. In this way, KREAP is able to process vertical and horizontal scratches (See Supplementary Fig. S2). In addition, we show that KREAP is suitable to process images with scratches in which only one side of the scratch is in the field of view (see Supplementary Fig. S2). We have not tested if KREAP is suitable for circular scratches. These points are now mentioned in the main text of the revised manuscript (lines 123-127) and are updated in the documentation of the software and in our homepage (https://erasmusmc-bioinformatics.github.io/KREAP/).

5. Line # 172: the parameters $\lambda$, $\mu m$ and A are mentioned but defined only later at line # 179. I would suggest to present them first at the location they are defined, otherwise it could be cryptic and confusing.

Author response: We appreciate that the reviewer pointed this out and we adjusted our manuscript accordingly to make it clearer.

6. Lines # 190-192: "Importantly, we have shown that the A parameter is correlated to the maximum surface area in pixels covered by cells". Why is this important? Isn't it trivial? I suggest to exclude this text.

Author response: As suggested by the reviewer, we decided to leave this statement out of the revised manuscript.

7. Line # 193: Would be insightful to explicitly provide the physical/biological interpretation of the 'performance value' ($\mu m$*A), which is not obvious to me – is it a standard measure in a different assay (then please provide a reference or at least an explanation)? Also, this measure is not used throughout the manuscript – why mention it then?

Author response: We agreed with the reviewer that as we do not refer to this measurement anywhere in our manuscript, we decided to leave it out of the revised version.

8. Line # 203-205: I do not see the point in this.

Author response: We removed lines 203-205 from the main text as well as panel (a) in Fig. 4.

9. Line # 213-217: Please report the fold change in addition to the p-value.

Author response: As indicated by the reviewer, we now provide the fold change in addition to the p-value (lines # 219-226).

10. Line # 219: Please describe the sub-dataset. How large is it? What was the criteria to select experiments from the full dataset?

Author response: We apologize for the confusion, but we actually used a complete dataset from the published study that consisted of a total of 214 image-series. This point was also adjusted in the revised manuscript in line # 227 and line # 230-231.

11. Line # 245-247: "detrimental effects" are not defined in the main text (should be independent of the figure legend).

Author response: In the revised manuscript, we defined the identification of adverse effects in the main text as well as in the legend (lines 253-264).

12. Line # 257-259: Please provide guidelines on how to determine if a given experiment is faulty (dead cells) or a strong effect caused by a perturbation? Especially when the software is not providing an image of the cells. Is there a way to decide based on the curves?

Author response: The software provides the raw images, segmentation output, and re-epithelialization curves (when successfully modelled) of each well. In addition, we implemented a "flagging" system in the software that helps the user to identify strong inhibitory or detrimental effects caused by a particular treatment. These flags or warnings are described in lines 262-264.

13. Legend on Figure 4: no reference to panel (b)

Author response: We appreciate the reviewer for pointing this out. We adjusted this point accordingly in the revised manuscript.

14. The "analysis image (right) does not fit in size to the other two and cannot be compared.

Author response: The raw images are re-scaled in the revised version of KREAP to match the segmentation output.

15. Figure 1: The "modify index" is not explained in the figure legend nor the main text. Is it just switching to the next well?

Author response: The modify index can be used to exclude a specific well from the analysis (e.g. when there is a technical error). This function is explained in the revised manuscript in lines 139-141.

16. Figure 2: It is hard to compare the input and output due to resizing – can this be fixed?

Author response: The raw images are re-scaled in the revised version of KREAP to match the segmentation output.

17. Figure 3 legend: make the first sentence bold to fit the other legends.

Author response: We thank the reviewer for this remark and changed the first sentence to bold to match the other legends.

18. The link to the github repository is broken (I could not get to the point of entering the username and password)

Author response: We apologise for the confusion, but the link of the Github repository is working. You need to provide the username (erasmusmc-review) and the password (erasmusmcreview123) on the top of the page (see red square below).

19. Methods should include detailed description of the algorithmic pipeline. Some of the main text could be transferred (and elaborated) in the Methods.

Author response: The algorithms can be found in the Github.

Close
