# Supplementary material for: KREAP: an automated Galaxy platform to quantify in vitro re-epithelialization kinetics
Source: Gigascience. 2018 Jun 28;7(7):giy078. doi: 10.1093/gigascience/giy078 (PMC6048990; doi:10.1093/gigascience/giy078)
Supplement: Reviewer_2_Report_(Original_Submission).pdf [file giy078_reviewer_2_report_(original_submission).pdf]

Reviewer Report

Title: **KREAP: An automated Galaxy Platform to Quantify in vitro Re-Epithelialization Kinetics**

Version: **Original Submission** **Date:** 11/28/2017

Reviewer name: **Dannon Baker**

Reviewer Comments to Author:

This is an informative article presenting KREAP, a Kinetic Re-Epithelialization Analysis Pipeline for use in Galaxy. The pipeline consists of several separate, complex tools wrapped into two comprehensive Galaxy tools, one for image analysis and one for data modeling, making it very easy to use. A virtual machine is provided encapsulating a Galaxy instance with the tools pre-installed, and the authors have made sample data available for demonstrating the pipeline's utility.The provided virtual machine was easy to configure and launch, and Galaxy starts automatically and is immediately available for use. Here I would make a discretionary revision recommendation that instead of shipping the .vmdk virtual drive it would be beneficial to utilize the .ova (Open Virtual Appliance) format for simpler, more standard distribution. A docker version would probably be well-received, too, as it's rapidly becoming the standard way to ship services like this. Lastly, it would be nice for these tools to ultimately be added to the Galaxy Tool Shed for non-VM distribution, though it looks like the dependencies might be cumbersome to deal with and I understand the distribution choices made here.The tool interfaces in Galaxy are straightforward and easy to use, especially considering the additional tutorial materials available. The tools worked well for me, with no errors. The only minor discretionary recommendation I have here is related to the somewhat cumbersome manifest file. It'd be more convenient, I think, to add an additional tool in Galaxy that can consume a collection to generate this file, or perhaps modify the initial image analysis tool itself to support collections, in addition to the pre-bundled zip format.In my opinion the work presented here is a solid, easy to use addition to the Galaxy ecosystem (not to mention the larger analysis space) in an area that is currently underserved and sometimes complicated to navigate. attached.

Level of Interest

Please indicate how interesting you found the manuscript: An article of importance in its field

Quality of Written English

Please indicate the quality of language in the manuscript: Acceptable

Declaration of Competing Interests

Please complete a declaration of competing interests, considering the following questions:

- Have you in the past five years received reimbursements, fees, funding, or salary from an organisation that may in any way gain or lose financially from the publication of this manuscript, either now or in the future?
- Do you hold any stocks or shares in an organisation that may in any way gain or lose financially from the publication of this manuscript, either now or in the future?

- Do you hold or are you currently applying for any patents relating to the content of the manuscript?
- Have you received reimbursements, fees, funding, or salary from an organization that holds or has applied for patents relating to the content of the manuscript?
- Do you have any other financial competing interests?
- Do you have any non-financial competing interests in relation to this paper?

If you can answer no to all of the above, write 'I declare that I have no competing interests' below. If your reply is yes to any, please give details below.

I declare that I have no competing interests

I agree to the open peer review policy of the journal. I understand that my name will be included on my report to the authors and, if the manuscript is accepted for publication, my named report including any attachments I upload will be posted on the website along with the authors' responses. I agree for my report to be made available under an Open Access Creative Commons CC-BY license (http://creativecommons.org/licenses/by/4.0/). I understand that any comments which I do not wish to be included in my named report can be included as confidential comments to the editors, which will not be published.

I agree to the open peer review policy of the journal

To further support our reviewers, we have joined with Publons, where you can gain additional credit to further highlight your hard work (see: https://publons.com/journal/530/gigascience). On publication of this paper, your review will be automatically added to Publons, you can then choose whether or not to claim your Publons credit. I understand this statement. Yes
